# Supplementary material for: Macrophage-induced enteric neurodegeneration leads to motility impairment during gut inflammation
Source: EMBO Mol Med. 2025 Jan 6;17(2):301–35. doi: 10.1038/s44321-024-00189-w (PMC11822118; doi:10.1038/s44321-024-00189-w)
Supplement: Supplementary file 1 — Appendix [file 44321_2024_189_MOESM1_ESM.pdf]

Title: **Macrophage-induced enteric neurodegeneration leads to motility impairment during gut inflammation**

Running Title: **Enteric Neurodegeneration after Surgery**

Mona Breßer[1]*, Kevin D. Siemens[1]*, Linda Schneider[1], Jonah E.Lunnebach[1], Patrick Leven[1], Tim R.Glowka[1], Kristin Oberländer[2], Elena De Domenico[3], Joachim L. Schultze[3,4,5], Joachim Schmidt[6], Jörg C.Kalff[1], Anja Schneider[2], Sven Wehner[1], Reiner Schneider[1]

[1] University Hospital Bonn, Department of Surgery, Bonn, Germany.

[2] German Center for Neurodegenerative Diseases (DZNE), Bonn, Germany; University of Bonn Medical Center, Dept. of Neurodegenerative Disease and Geriatric Psychiatry/Psychiatry, Bonn, Germany.

[3] Deutsches Zentrum für Neurodegenerative Erkrankungen (DZNE). PRECISE Platform for Genomics and Epigenomics at DZNE and University of Bonn, Bonn, Germany

[4] Systems Medicine, Deutsches Zentrum für Neurodegenerative Erkrankungen (DZNE), Bonn, Germany

[5] Genomics and Immunoregulation, Life & Medical Sciences (LIMES) Institute, University of Bonn, Bonn, Germany

[6] University Hospital Bonn, Department of General, Thoracic and Vascular Surgery, Bonn, Germany.

*****These authors contributed equally to the work.

Correspondence:

Dr. Reiner Schneider, University of Bonn, Department of Surgery, Venusberg-Campus 1, 53127 Bonn, Germany, phone: +49-228-287-13672, Reiner.Schneider@ukbonn.de

Keywords: Enteric Neurons/ Neuroimmune Interaction/ Postoperative ileus/ Synaptic Damage

Table of Content:

Page 2-6: Appendix Table S1-3

Page7-14: Appendix Figures and according legends 1-4

Appendix Tables

Appendix **Table S1:** Antibodies used in the study.

Host	Target	Fluorophore	Clone	Supplier	Art. No.	Application
**Human**	ANNA1	---		Mayo Clinic		IHC
**Goat**	ChAT	---	Polyclonal	Merck	AB144P	IHC
**Rabbit**	HA (C29F4)	---	Monoclonal	Cell signaling	3724S	IHC
**c**	cFos	---	Polyclonal	Santa Cruz	sc52	IHC
**Rabbit**	Ki67	---	Monoclonal	Abcam	ab16667	IHC
**Chicken**	GFP	---	Polyclonal	Novus biologicals	NB100-1614	IHC
**Chicken**	Synapsin 1/2	---	Polyclonal	Synaptic System	106006	IHC
**Rabbit**	Synapsin 1/2	---	Polyclonal	Synaptic System	106002	IHC
**Mouse**	Synapsin 1/2/3	---	Monoclonal	Biolegend	853701	IHC
**Mouse**	Tubb3	---	Monoclonal	Biolegend	801202	IHC
**Mouse**	PSD95	---	Monoclonal	Biolegend	BLD-810401	IHC
**Guinea Pig**	PSD95	---	Monoclonal	Synaptic System	124308	IHC
**Mouse**	PSD95	---	Monoclonal	Synaptic System	124011	IHC
**Rabbit**	Cleaved-Caspase 3	---	Monoclonal	Cell Signaling	cs 9664	IHC
**Rabbit**	Iba1	---	Monoclonal	Abcam	178847	IHC
**Rat**	CD68	---	Monoclonal	Bio Rad	MCA1957	IHC
**Rat**	MHC II	---	Monoclonal	Biolegend	107602	IHC
**Donkey**	Rabbit	Alexa647	Polyclonal	Dianova	711–606–152	IHC
**Donkey**	Rabbit	Alexa488	Polyclonal	Dianova	711-095-152	IHC
**Donkey**	Rabbit	Cy-3	Polyclonal	Dianova	711-165-152	IHC
**Donkey**	Goat	Cy-3	Polyclonal	Dianova	705-165-147	IHC
**Donkey**	Chicken	FITC	Polyclonal	Jackson	703-095-155	IHC
**Donkey**	Rat	Alexa488	Monoclonal	Invitrogen	A21208	IHC
**Donkey**	mouse	Cy-3	Polyclonal	Dianova	711-165-141	IHC
**Donkey**	Human	DyLight488	Polyclonal	Invitrogen	SA5-10126	IHC
**Donkey**	Human	DyLight650	Polyclonal	Invitrogen	SA5-10129	IHC
**Donkey**	Guinea Pig	CF633	Polyclonal	SIGMA	SAB4600129	IHC
**---**	---	Hoechst	---	Invitrogen	H3570	IHC+FACS
**Mouse**	CD45	Pacific Blue	30-F11	Biolegend	103126	FACS
**Mouse**	Ly6C	APC	HK1.4	eBioScience	17-5932-82	FACS

**Mouse**	MHCII	Alexa647	M5/114.15.2	Biolegend	107617	FACS
**Mouse**	CD11c	PE	N418	Biolegend	117307	FACS
**Mouse**	CD16/32	PE/Cy7	93	Biolegend	101317	FACS
**Mouse**	CD36	PE	HM36	Biolegend	102605	FACS

Appendix **Table S1.** Information on all antibodies used for IHC and FACS in the study.

Appendix Table S2: Human subjects and GI surgical specimens for analysis of neurodegeneration

#	Patient ID	Sex	Age	Start Time of procedure	Collection time points	Previous illness	Medications
**1**	160331_hME	male	85	*08:50*	*10:50 and 12:16*	none	Ursofalk (gallstone reducer)<br>Simavastin (Chlosterol reducer)<br>**Pantozol**<br>**Clexane** (coagulation inhibitor)
**2**	180220_hME	male	80	*8:30*	*12:20 and 13:10*	benign hyperplasia of the prostate gland<br>**art. hypertension**<br>cardiac arrhythmia<br>duodenal ulcer	**Pantazol**<br>Propafenone HCl (cardiac arrhythmia)<br>**Alna**<br>Valsartan/<br>Metohexal (antihypertensive drugs)
**3**	181029_hME	female	73	*07:45*	*10:30 and 11:15*	COPD<br>Myxoma<br>**Diabetis mellitus Type II**<br>**hypertension**<br>hyperlipidemia	Berotec (COPD)<br>Inuvair (COPD)<br>Spiriva (COPD)<br>**clexane**<br>Bisoprolol (blood pressure reducer)<br>**Ramipril**<br>HCT (diuretic drug)<br>Marcumar<br>**Atorvastatin**
**4**	190506_hME	male	76	*8:55*	*12:12 and 13:00*	Ulcera Duodeni<br>reflux esophagitis II°<br>sigma diverticulosis<br>**Diabetis mellitus Type II**	ASS<br>**Atorvastatin** (cholesterol reducer)<br>Gabapentin (antiepilepthic)<br>Amitriptilin (antidepressant)
**5**	190808_hME	female	70	*10:00*	*13:40 and 18:20*	**pul./art. hypertension**<br>mitral-/tricuspidal regurgitation<br>chron. pancreatitis<br>sec. **Diabetis mellitus Type 3c**<br>kidney insufficiency (dialysis)<br>vein thrombosis	Marcumar (antikoagulans)<br>**Atorvastatin**<br>**Pantozol**<br>Bisopropol (ß-blocker)<br>Torem (urine excretion)<br>Amitriptylin
**6**	191017_hME	male	75	*9:11*	*13:38 and 15:22*	silicosis<br>gastritis	**Pantozol**<br>Eliquis (coagulation inhibitor)<br>**Alna**
**7**	191023_hME	male	57	*9:11*	*11:20 and 14:10*	cholestasis<br>acute pancreatitis<br>NSTEMI (myocardial infarction)<br>**art. hypertension**<br>obesity<br>**Diabetis mellitus Type II**<br>hyperlipidemia	Levemir (insulin)<br>ASS<br>Amlodipin<br>Metformin (antidiabetic)<br>**Pantozol**<br>Rekawan (potassium donor)<br>Simvastin (cholesteol reducer)
**8**	201210_hME	female	60	*9:35*	*11:25 and 14:00*	Pantoprazol<br>Bisoprolol<br>Ramipril	**Unacid**

						Levothyroxin<br>HCT	
**9**	211216_hME	female	69	*8:55*	*10:22 and 12:42*	liver cirrhosis Child Pugh A<br>diabetis mellitus II<br>hypothyreosis	**Insulin Lispro**<br>**Lisinopril**<br>**Levothyroxin**<br>**Fluoxetin**<br>**Clexane**<br>**Pantoprazol**<br>**Torasemid**<br>**Colecalciferol**<br>**Doxazosin**<br>**Metformin**
**10**	210208_hME	male	60	*9:07*	*11:10 and 12:56*	chron. Pancreatitis<br>thrush esophagitis<br>gastritis type C<br>colonic polyps<br>adrenal adenomas<br>sigma diverticulitis<br>steatosis hepatis	**Sertralin**<br>**Metformin**<br>**Ferro Sanol**<br>**Zoplicon**<br>**Pantoprazol**<br>**Candesartan**<br>**Insulin Toujeo**
**11**	210412_hME	male	61	*9:03*	*10:55 and 12:53*	aneurysm (A. hep. Communis)<br>hemophilia A<br>chron. Hepatitis C<br>liver transplantation	Everolimus<br>Tacrolimus<br>Tamsulosin<br>Enalapril<br>Ursofalk<br>Pantoprazol
**12**	220405_hME	female	80	*9:19*	*10:55 and 12:42*	Mamma carcinoma	Tamoxifen<br>Clexane<br>Pantoprazol<br>L-Thyroxin
**13**	220411_hME	female	72	*9:08*	*10:35 and 12:15*	art. Hypertension<br>diabetis mellitus II<br>mamma carcinoma	Pantoprazol<br>Levothyroxin<br>Dapagliflozin<br>Candesartan<br>Bisoprolol<br>Insulin glargin<br>Metformin<br>Kalinor P
**14**	230202_hME	female	64	*9:06*	*10:56 and 12:48*	peripheral arterial disease<br>constriction of inner carotis<br>art. hypertension	ASS<br>Allopurinol (uric acid reducer)<br>Bisohexal/Candesartanicilex etil (blood pressure reducer)<br>**Pantozol**
**15**	231129_hME	female	59	*10:02*	*11:22 and 13:42*	koronare Eingefäßerkrankung<br>latente Hyperthyreose<br>Struma multinodosa<br>Arthrose<br>Polyneuropathie	Simvastatin<br>Amlodipin

Appendix Table S2: Information on 15 patients who underwent pylorus-preserving pancreaticoduodenectomy and gave written consent to procure jejunal tissue for histological and transcriptional studies. The tissue was used for histology, RNA isolation, and gene expression analysis. The study was conducted under protocols permitted by the ethical committee of the University of Bonn, Germany.

Appendix **Table S3.** Primer pairs for qPCR analysis.

Gene	Forward primer	Reverse primer
18S	GTAACCCGTTGAACCCCATT	CCATCCAATCGGTAGTAGCG
mChAT	TCATTAATTTCCGCCGTCTC	AGTCCCGGTTGGTGGAGTC
mSyn1	ATTCTCTGTGGACATGGAAGTT	AATGACCAAACTTCGGTAGTCT
mArg1	TTGGGTGGATGCTCACACTG	TTGCCCATGCAG ATTCCC
mEgr1	GAGCGAACAACCCTATGAGC	GGCCAGTATAGGTGATGGGA
mCcl2	CCCAATGAGTAGGCTGGAGA	GCTGAAGACCTTAGGGCAGA
mIl6	AAGTCGGAGGCTTAATTACACATGT	CCATTGCACAACTCTTTTCTCATT
mBcl-xL	GTCCCGCCTCTTCACCTTTCAG	GATTCTGGTGTTTCCCCGTTGG
mBcl2	AACATCCCAGCTTCACATAACCCC	GCGACCCCAGTTTACTCCATCC
mCx3cr1	CCTGTTATTTGGGCGACATT	ACGCCCAGACTAATGGTGAC
mDlg4	ACCAGAAGAGTATAGCCGATTCG	GGTCTTGTCGTAGTCAAACAGG
m Snap25	CAACTGGAACGCATTGAGGAA	GGCCACTACTCCATCCTGATTAT

Appendix **Table S3.** Information on all qPCR primer pairs used in the study.

Appendix Figures

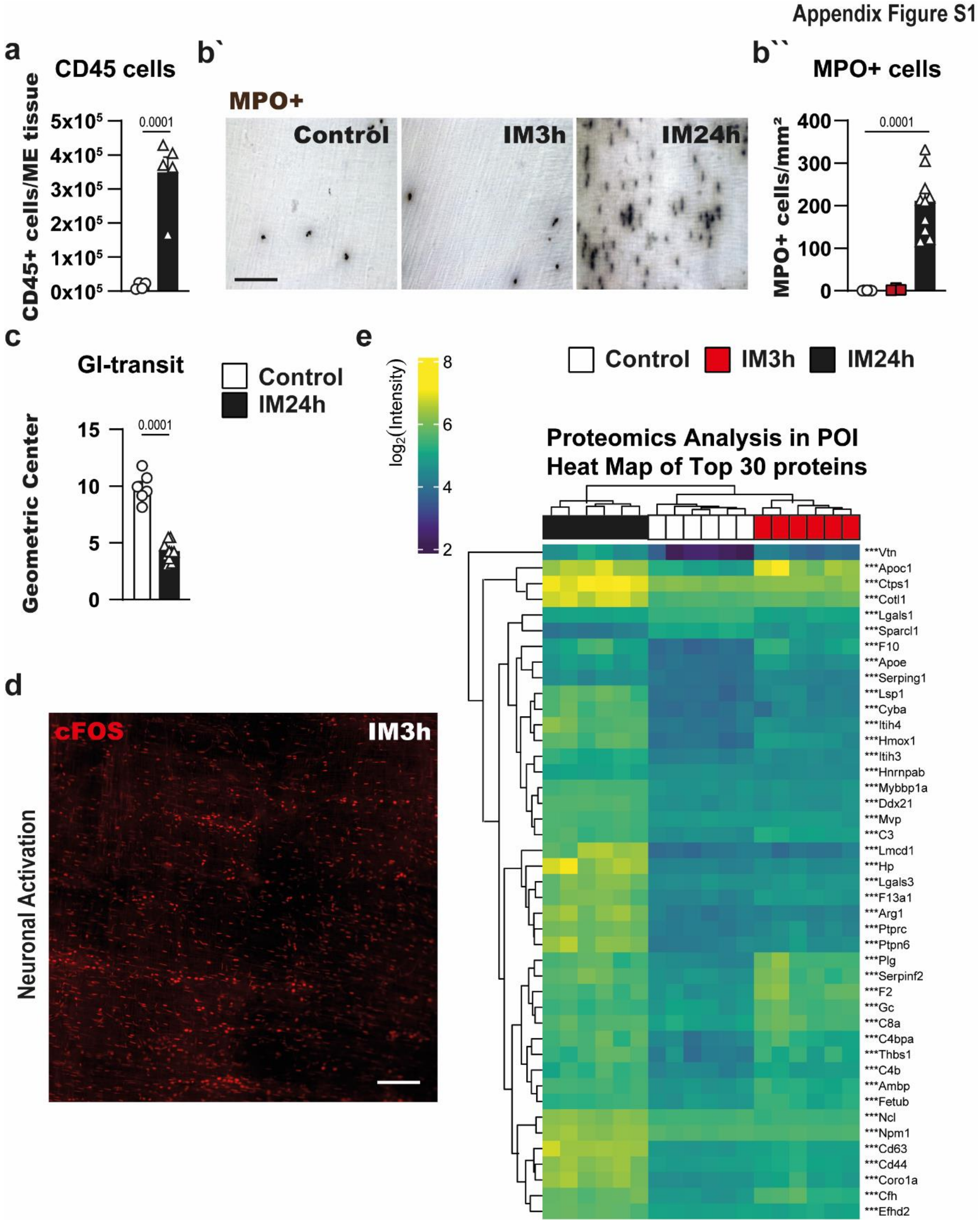

Appendix Figure S1: Intestinal manipulation and inflammation activate enteric neurons.

(**a**) FACS analysis of CD45$^+$ cells in the *muscularis externa* (*ME*) of control and IM animals shows strong induction of immune cell infiltration in POI. Bar graphs show the mean CD45$^+$ cell number normalized to the *ME* tissue weight. n = 4(control), 5 (IM24h).

(**b´**) Immunohistological analysis of activated (MPO$^+$, black) leukocytes in animals 3h, 24h post IM and controls. Scale bar 100 µm. (**b´´**) Quantification of MPO$^+$ cells per mm² jejunum *ME* tissue in control and IM animals. At IM24h, the disease peak, immune cell infiltrate is present in the *ME* represented by clusters of MPO$^+$ cells. Bar graphs show the mean MPO$^+$ cell number normalized to the *ME* area. n = 7 (control), 6 (IM3h), 10 (IM24h).

(**c**) Gastrointestinal (GI) transit analysis with FITC-dextran in animals 24h post-IM and control. IM24h mice show a delay in GI transit time. n = 6 (control), 10 (IM24h).

(**d**) Immunohistochemistry analysis of activated cells (cFOS$^+$, red) 3 hours post intestinal manipulation (IM). Many double-positive cells were detected in an overview image of jejunum *ME* tissue at IM3h. Scale bar 500 µm.

(**e**) Mass spectrometry analysis of control and POI mice. Heat map of the top 30 proteins regulated in the *ME* during POI shows differences between all 3 time points. Bar graphs show the fold gene induction normalized to control mice.

Statistical analysis is based on Fisher's exact *t*-test (e), Student´s *t*-test (a and c), and one-way ANOVA (b). Standard deviations are presented as SEM.

Appendix Figure S2

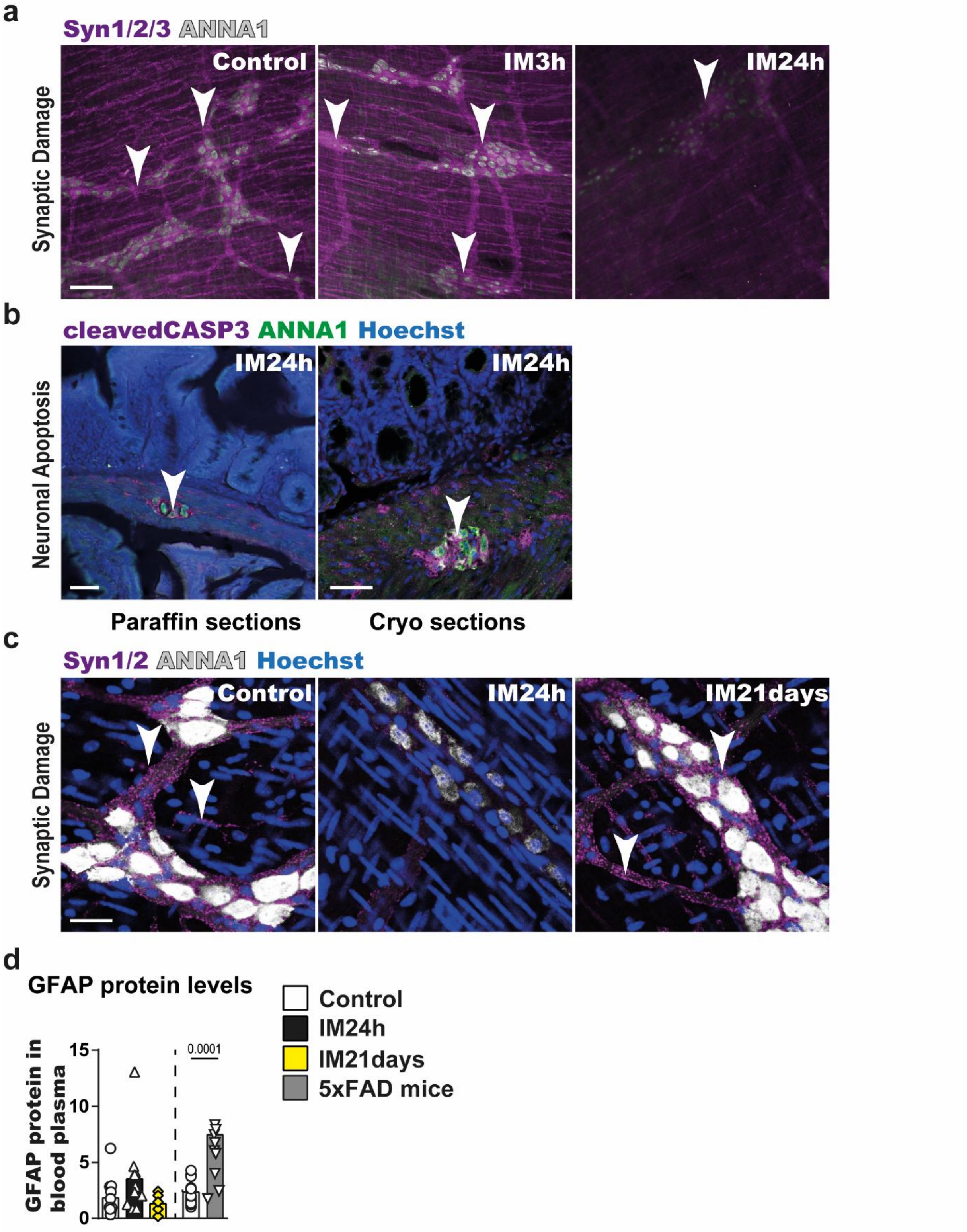

Appendix Figure S2: Intestinal manipulation and inflammation induce enteric neurodegeneration

(**a**) Immunohistochemistry analysis of enteric neurons (myenteric plexus, ANNA1$^+$, grey) and synaptic structures (SYN1/2/3, violet) 3h and 24h post IM and in control. At disease peak, IM24h, neurodegeneration is characterized by a weak expression of synaptic proteins, e.g., synapsins (white arrowheads). Scale bar 50 µm.

(**b**) Immunohistochemistry analysis of apoptotic (cleaved-CASP3$^+$, red) enteric neurons (myenteric plexus, ANNA1$^+$, grey) 24 hours post IM in cryo and paraffin sections. Double-positive cells (white arrowheads) were detected in the ganglia of the jejunum. Scale bar 50 µm.

(**c**) Immunohistochemistry analysis of enteric neurons (ANNA1$^+$, grey) and synaptic structures (SYNAPSIN 1/2/3, violet) 24h and 21 days post IM and in control. At disease peak, IM24h, strong neurodegeneration is characterized by a weak expression of synaptic proteins, e.g., SYNAPSINS (white arrowheads). Scale bar 50 µm.

(**d**) Blood Plasma levels of neurofilament light chain (NfL) in POI, control and 5xFAD (Alzheimer´s) mice. Plasma samples were analyzed with a SIMOA assay (Neurology 4-Plex E Advantage Kit) using the *Quanterix* system. The protein amounts are significantly increased in 5xFAD animals. Bar graphs show protein levels of blood plasma in pg/ml. n = 13 (control1), 15 (control2), 11 (IM24h), 6 (IM21days).

Statistical analysis is based on one-way ANOVA (d). Standard deviations are presented as SEM.

Appendix Figure S3

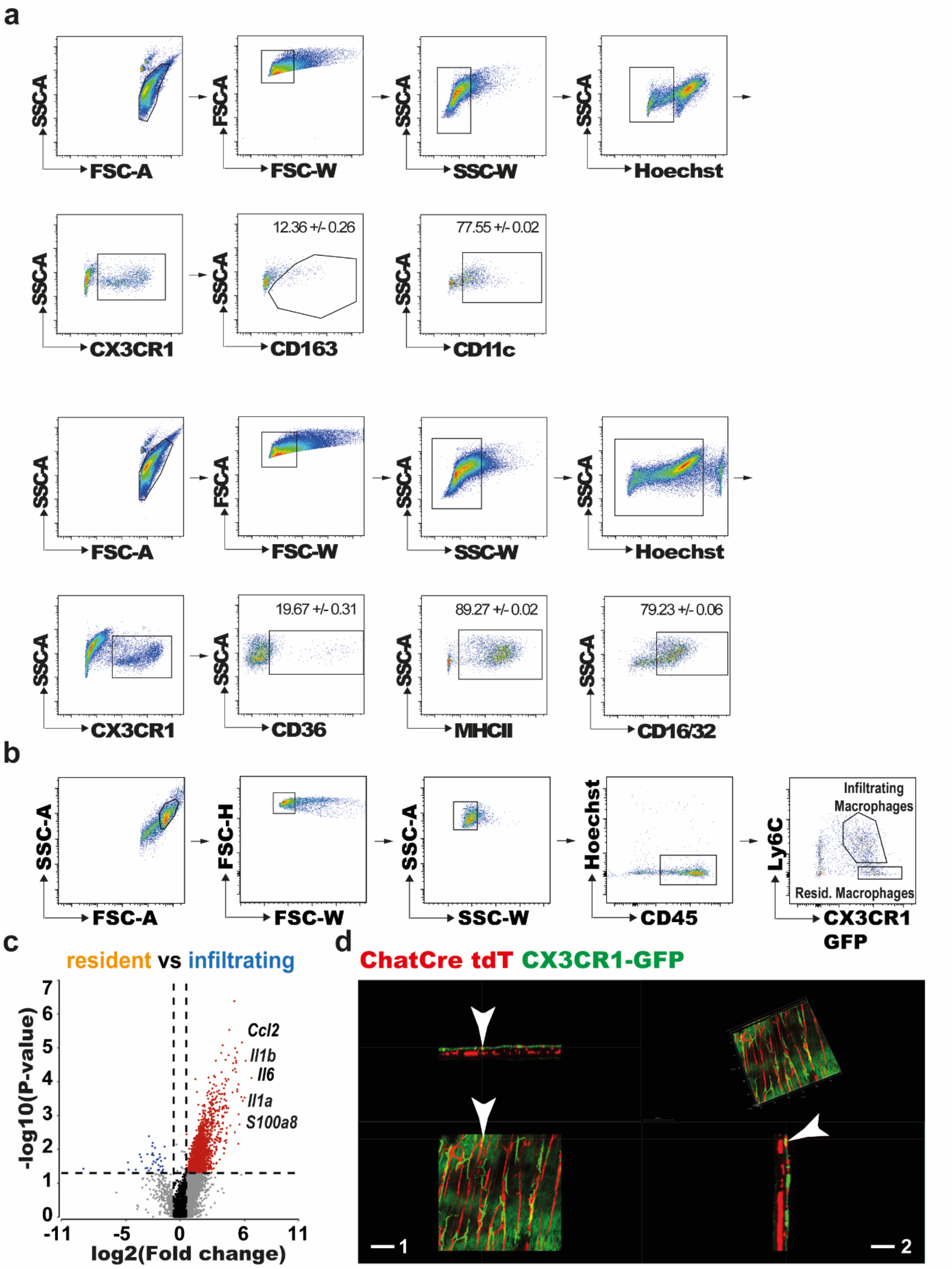

Appendix Figure S3: Inflammatory macrophages are causing enteric neurodegeneration.

(**a**) Gating strategy of the FACS analysis to characterize resident macrophages from the *ME* samples of Cx3cr1$^{GFP/+}$ mice with several inflammatory markers as indicated.

(**b**) Gating strategy of the FACS analysis to sort infiltrating and resident macrophages from the *ME* samples of Cx3cr1$^{GFP/+}$ mice at IM3h, IM24h, and control conditions.

(**c**) Volcano plot showing significantly changed genes between resident and infiltrating monocyte-derived macrophages at the IM24h time point. The plot depicts 2357 up- and 41 downregulated genes with a fold change ≥1.5.

(**d**) Immunohistochemistry analysis of enteric neurons (myenteric plexus, Chat-Cre-tdTomato, red) and resident macrophages (CX3CR1-GFP^{+}, green) 24 hours post-IM. CX3CR1-GFP^{+} macrophage phagocyte red tdT structures (white arrowheads). Scale bar 1: 10 µm and Scale bar 2: 100 µm.

Statistical analysis is based on Fisher's exact *t*-test (c).

Appendix Figure S4

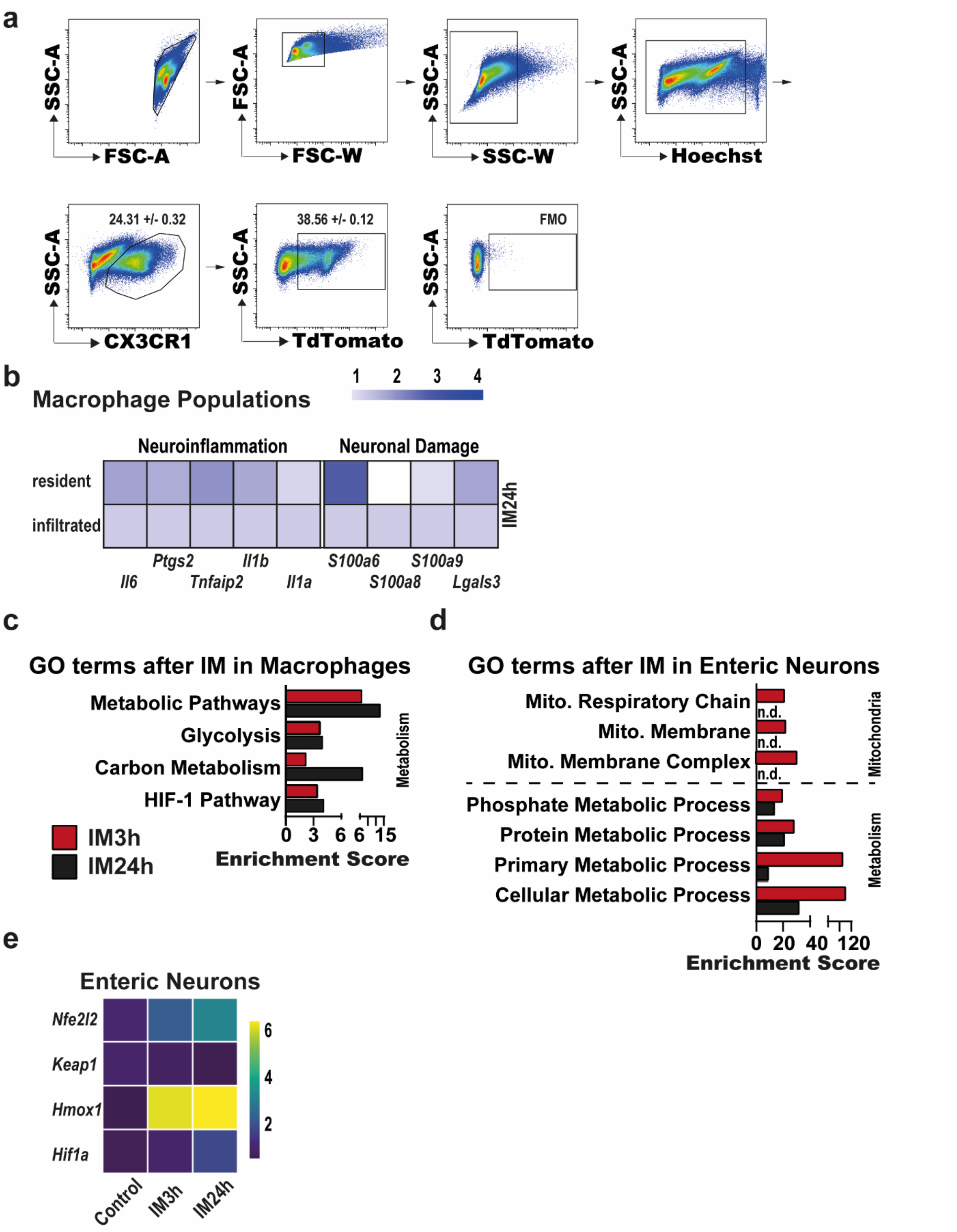

Appendix Figure S4: Inflammatory resident macrophages are causing metabolic stress.

(**a**) Gating strategy of the FACS analysis to quantify macrophages that phagocyte neuronal structures from the *ME* samples of Cx3cr1$^{GFP/+}$ Chat-Cre- Ai14(tdTomato) mice.

(**b**)The heat map of genes shows significantly changed genes connected to neuronal damage and neuroinflammation in resident macrophages compared to infiltrating macrophages at IM24h. Resident macrophages displayed a stronger expression of factors related to neurodegeneration. Heat map values were generated by normalization of the gene expression to infiltrating macrophages.

(**c**) GO analysis of significantly changed genes ($p < 0.05$) shows strong induction of GO terms connected to metabolic processes, such as glycolysis and the HIF-1 pathway.

(**d**) Gene ontology analysis of significantly changed genes ($p < 0.05$) showing strong induction of GO terms connected to metabolic and mitochondrial changes in enteric neurons during POI. At IM3h, gene clusters for metabolic processes were significantly enriched but diminished (metabolic) or even absent (mitochondrial) at IM24h.

(**e**) The heat map of genes connected to metabolic stress significantly changed in enteric neurons of IM3h and control animals. IM induced the expression of classical redox stress genes in enteric neurons. Heat map values were generated by normalization to the expression in enteric neurons of control mice.

Statistical analysis is based on Fisher's exact *t*-test (b-e).
