# Supplementary figures and images for: Macrophage-induced enteric neurodegeneration leads to motility impairment during gut inflammation
Source: EMBO Mol Med. 2025 Jan 6;17(2):301–35. doi: 10.1038/s44321-024-00189-w (PMC11822118; doi:10.1038/s44321-024-00189-w)

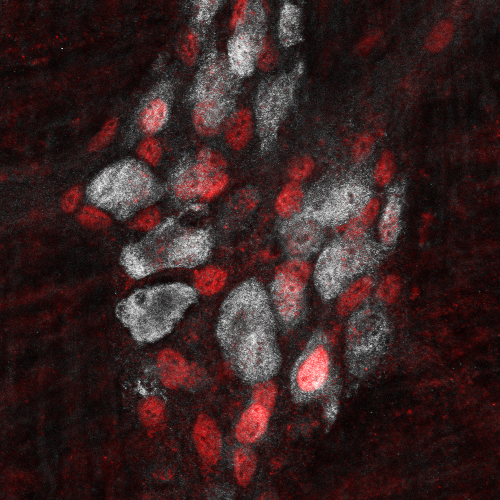

Supplement: Supplementary file 3 — Source data Fig. 1 [file 44321_2024_189_MOESM3_ESM.zip › 1/1c/PSD95_FOS_Anna1.lif_Series007_ch02_overlay_Figure1c_Cut_IM3h.tif]

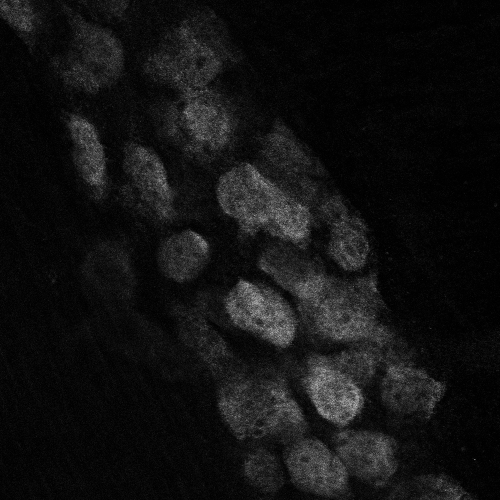

Supplement: Supplementary file 3 — Source data Fig. 1 [file 44321_2024_189_MOESM3_ESM.zip › 1/1c/PSD95_FOS_Anna1.lif_Series008_ch02_overlay_Control_Cut_1c.tif]

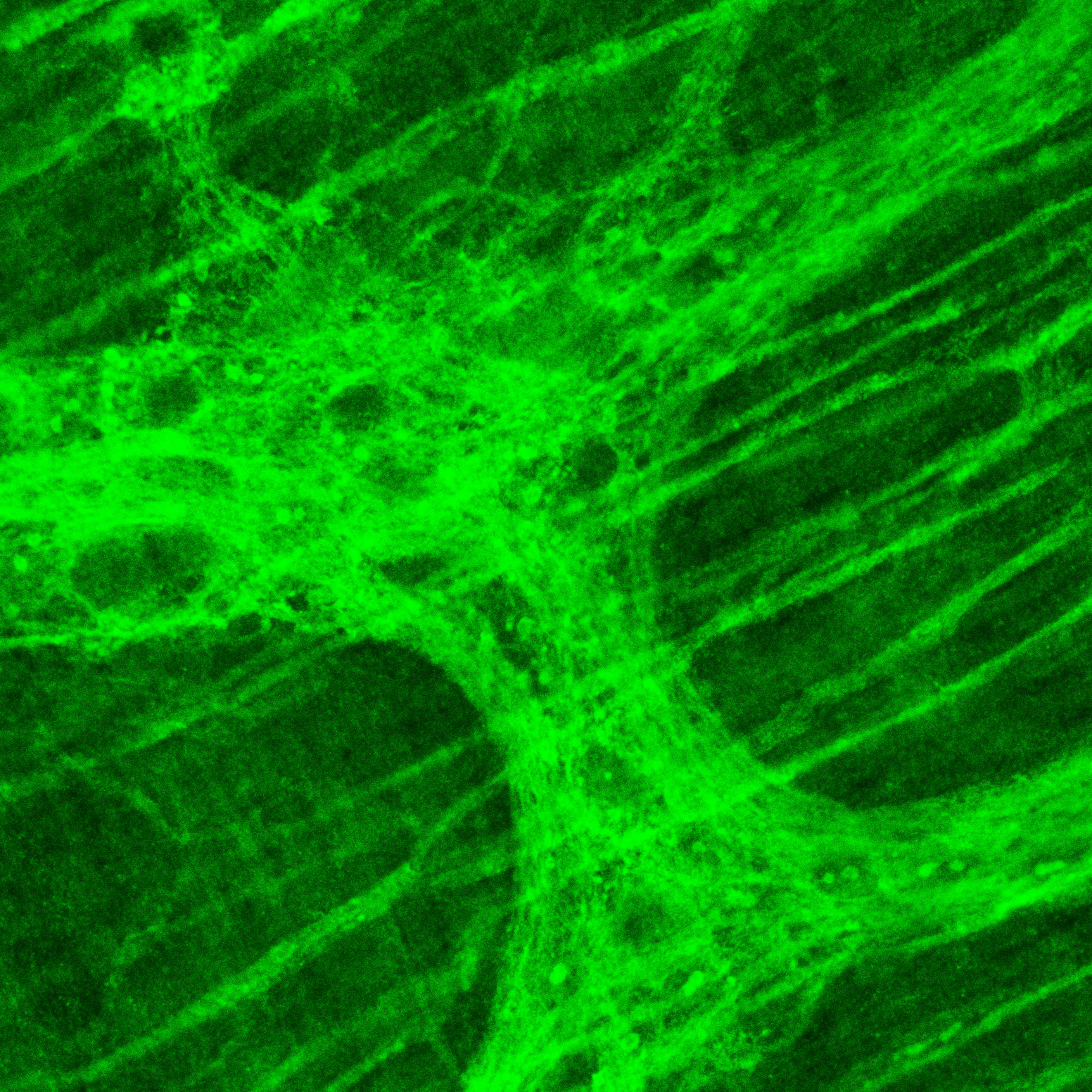

Supplement: Supplementary file 4 — Source data Fig. 2 [file 44321_2024_189_MOESM4_ESM.zip › 2/2a/2a_Control_green2.jpg]

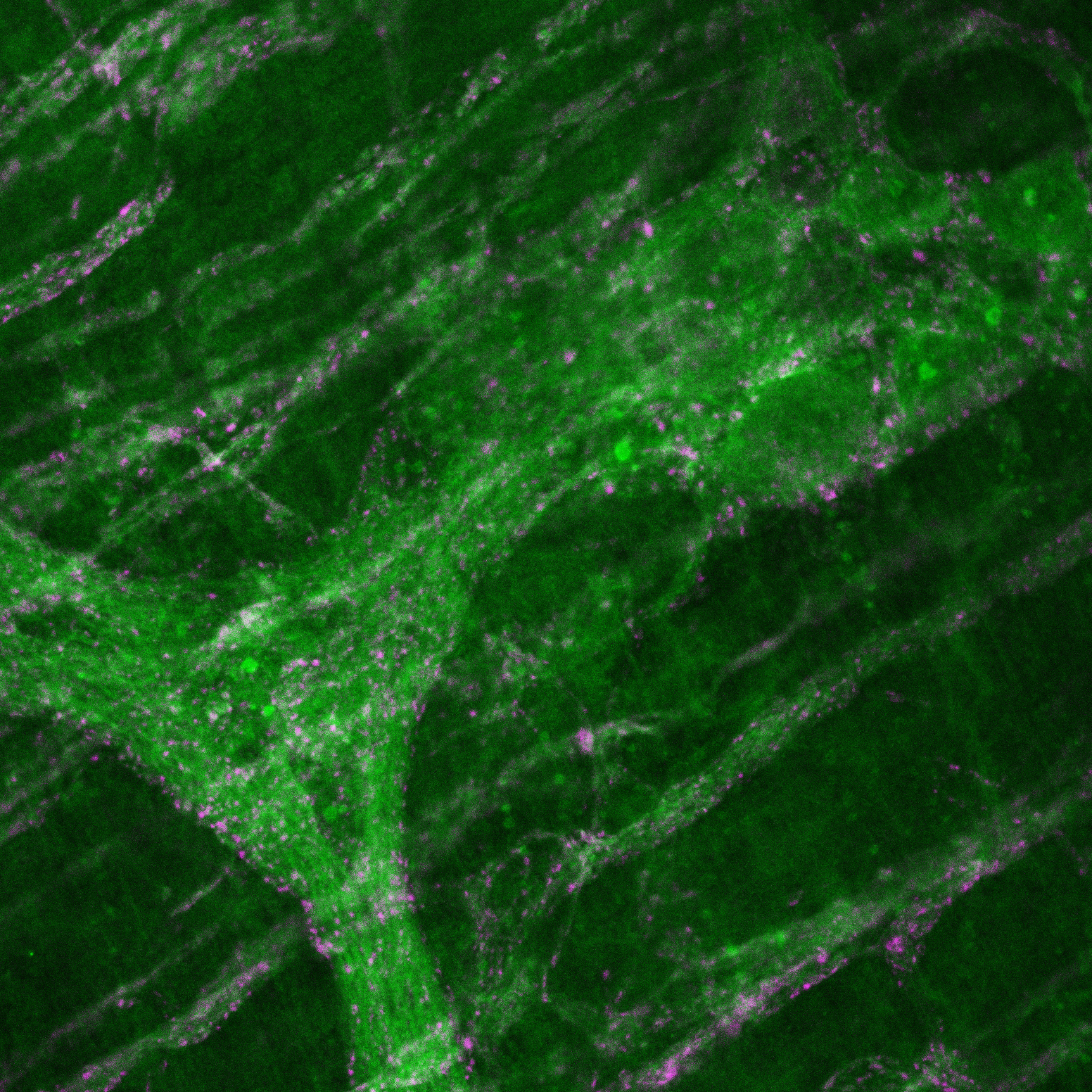

Supplement: Supplementary file 4 — Source data Fig. 2 [file 44321_2024_189_MOESM4_ESM.zip › 2/2a/2a_IM24h_merge.jpg]

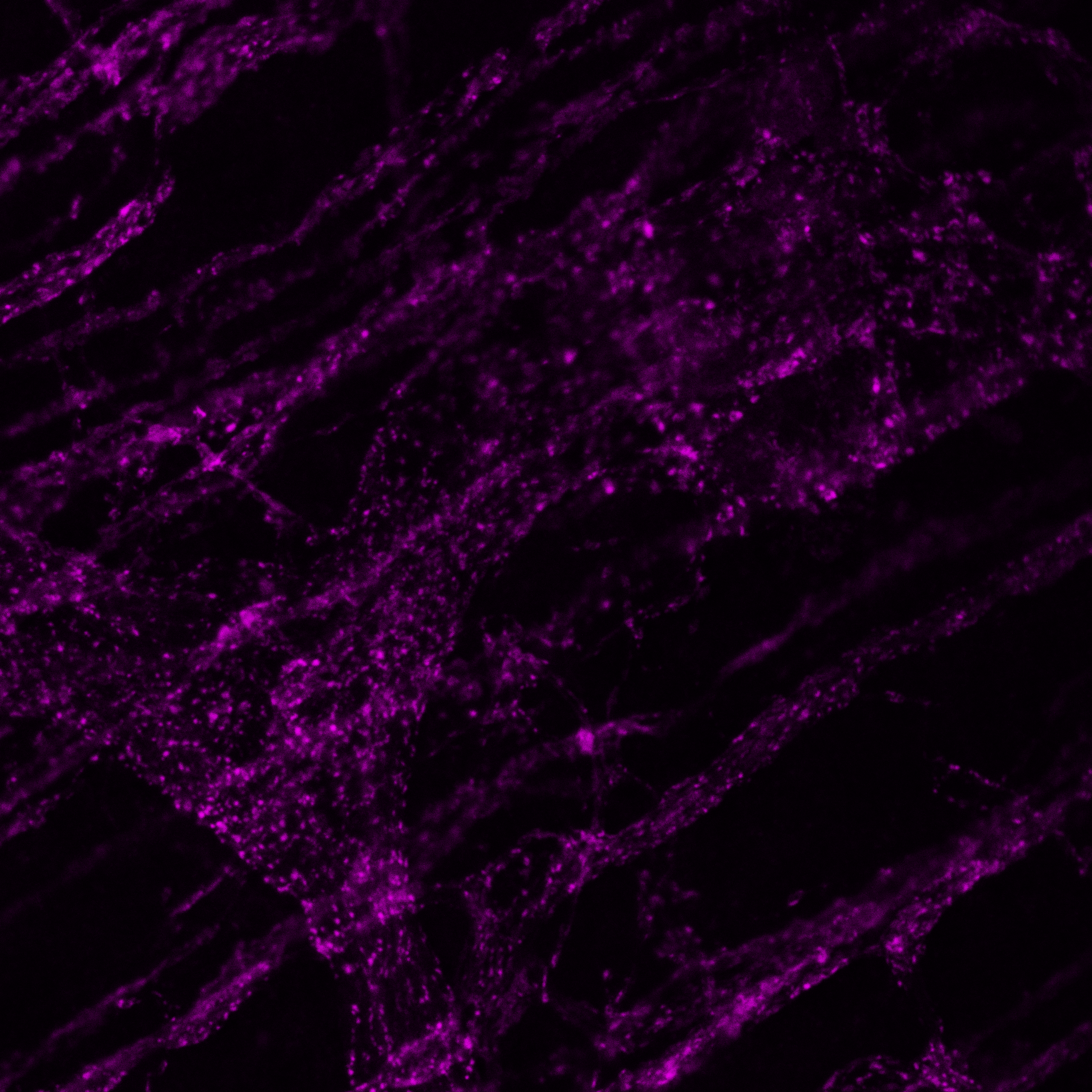

Supplement: Supplementary file 4 — Source data Fig. 2 [file 44321_2024_189_MOESM4_ESM.zip › 2/2a/2a_IM24h_violet2.jpg]

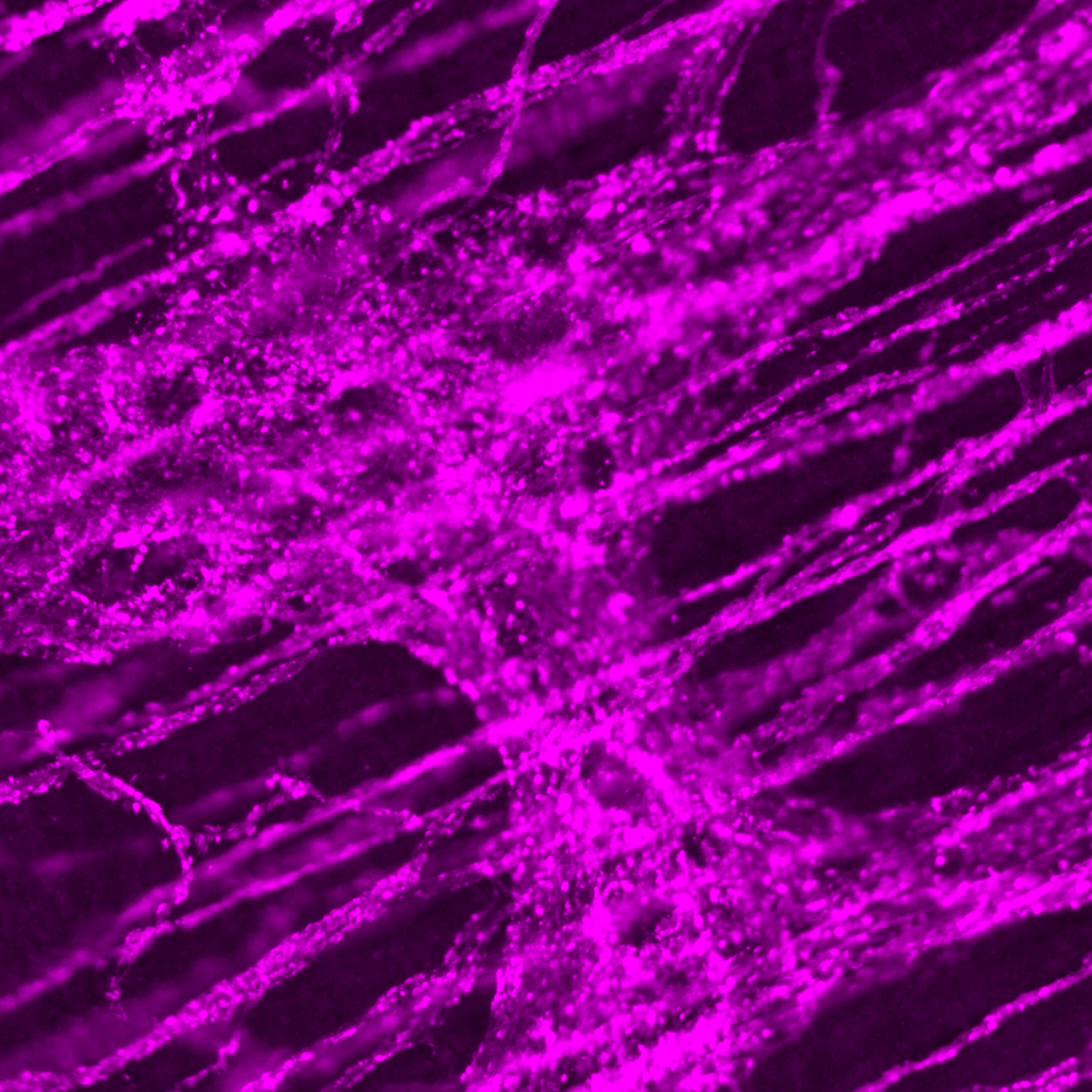

Supplement: Supplementary file 4 — Source data Fig. 2 [file 44321_2024_189_MOESM4_ESM.zip › 2/2a/2a_Control_violet2.jpg]

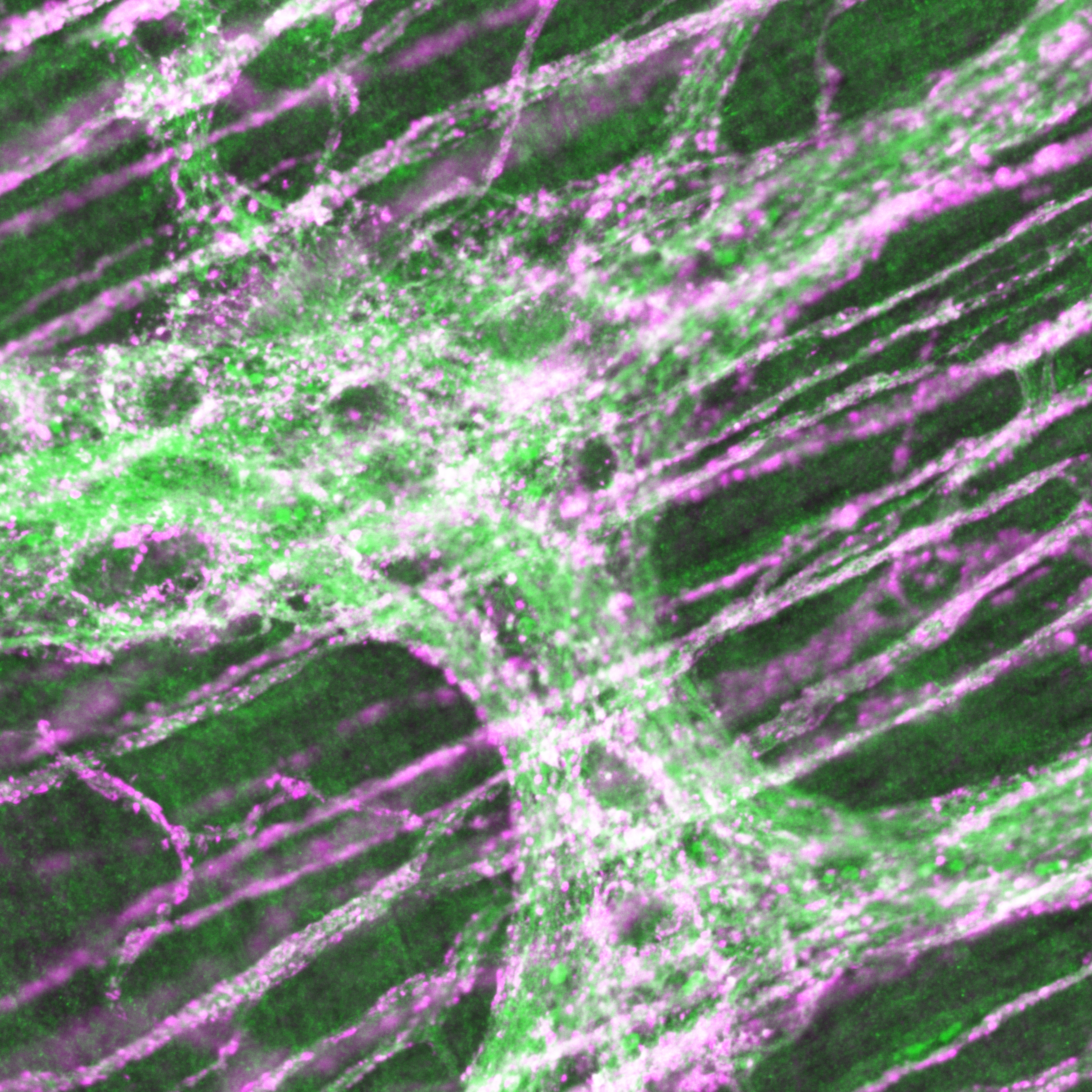

Supplement: Supplementary file 4 — Source data Fig. 2 [file 44321_2024_189_MOESM4_ESM.zip › 2/2a/2a_Control_merge.jpg]

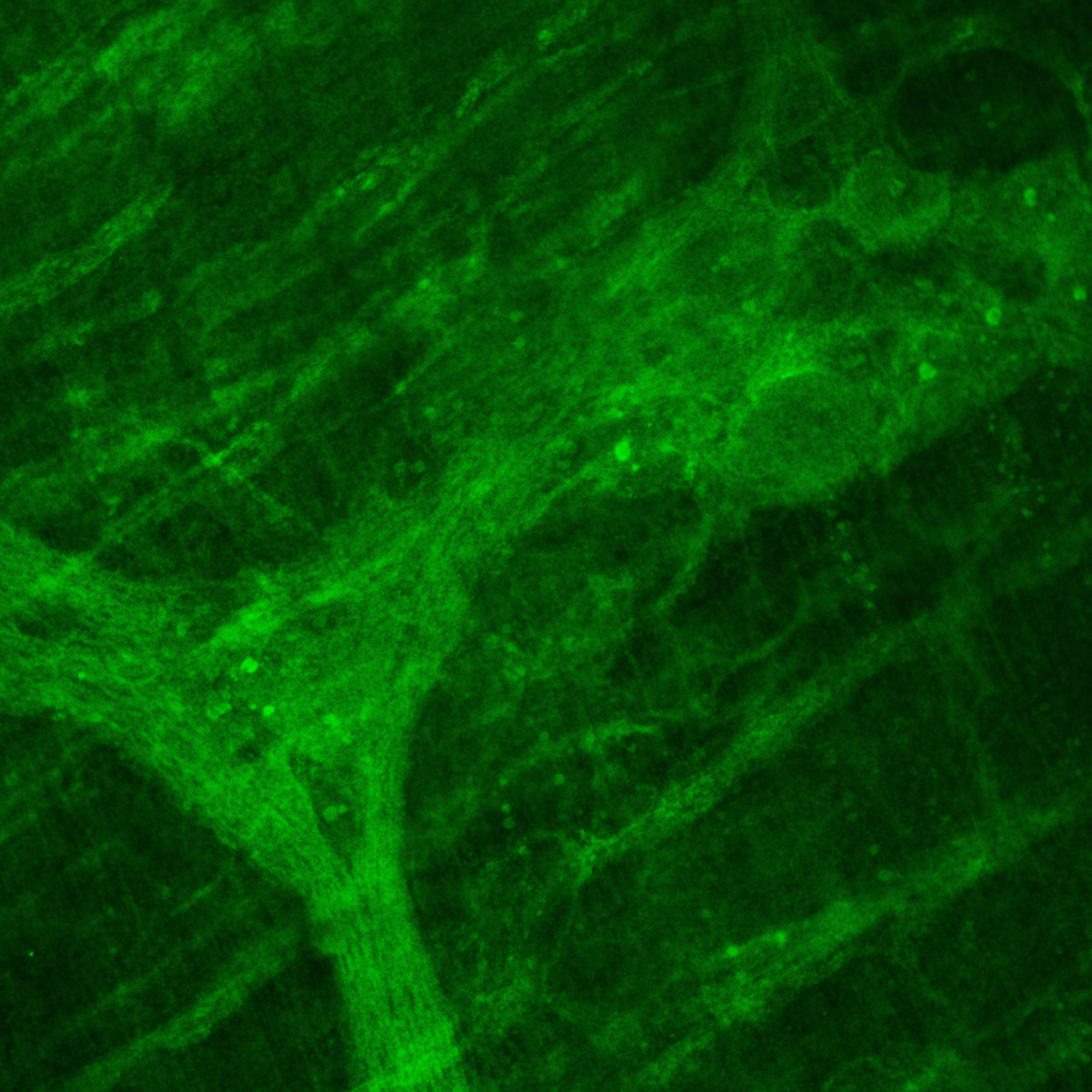

Supplement: Supplementary file 4 — Source data Fig. 2 [file 44321_2024_189_MOESM4_ESM.zip › 2/2a/2a_IM24h_green2.jpg]

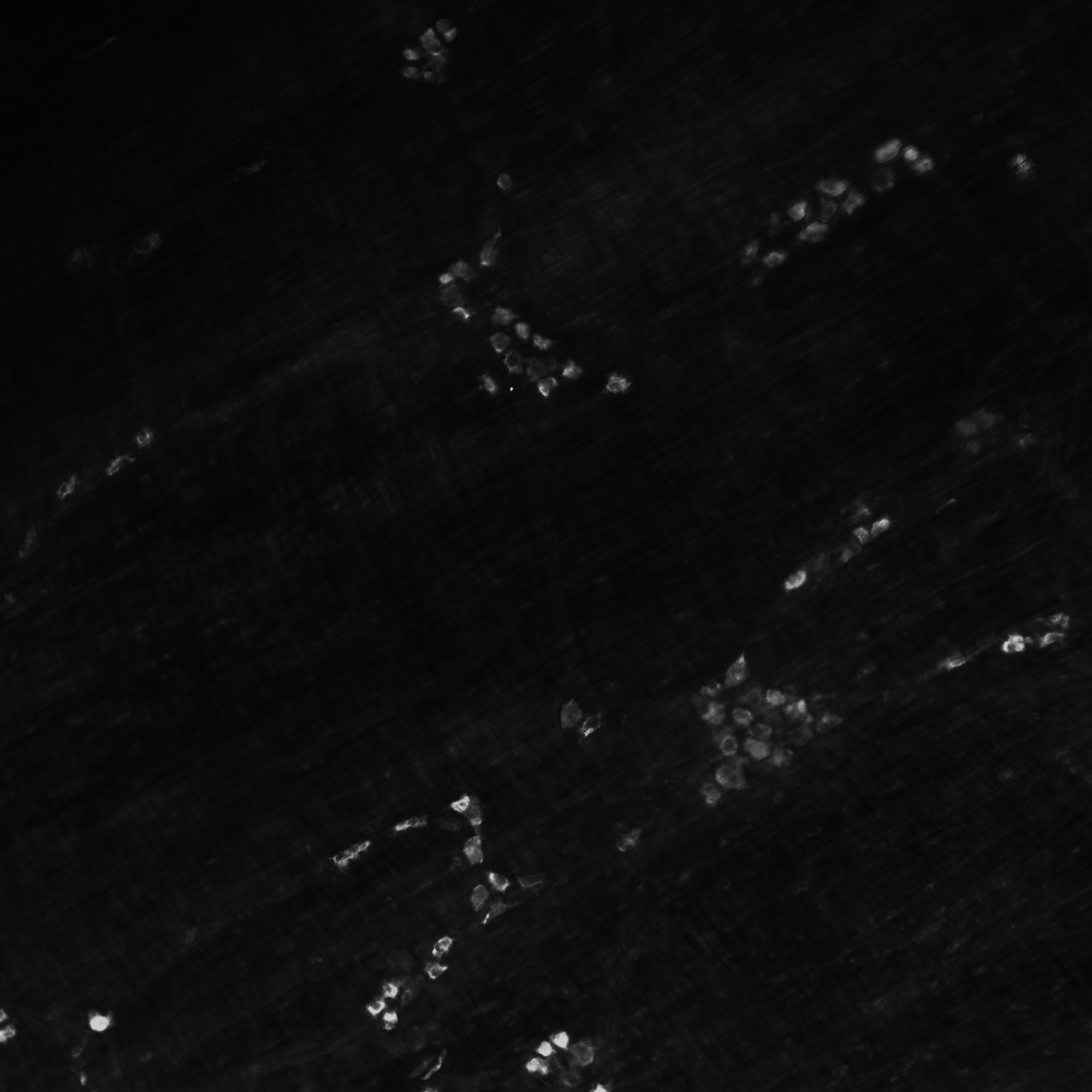

Supplement: Supplementary file 4 — Source data Fig. 2 [file 44321_2024_189_MOESM4_ESM.zip › 2/2d/IM24h.tif]

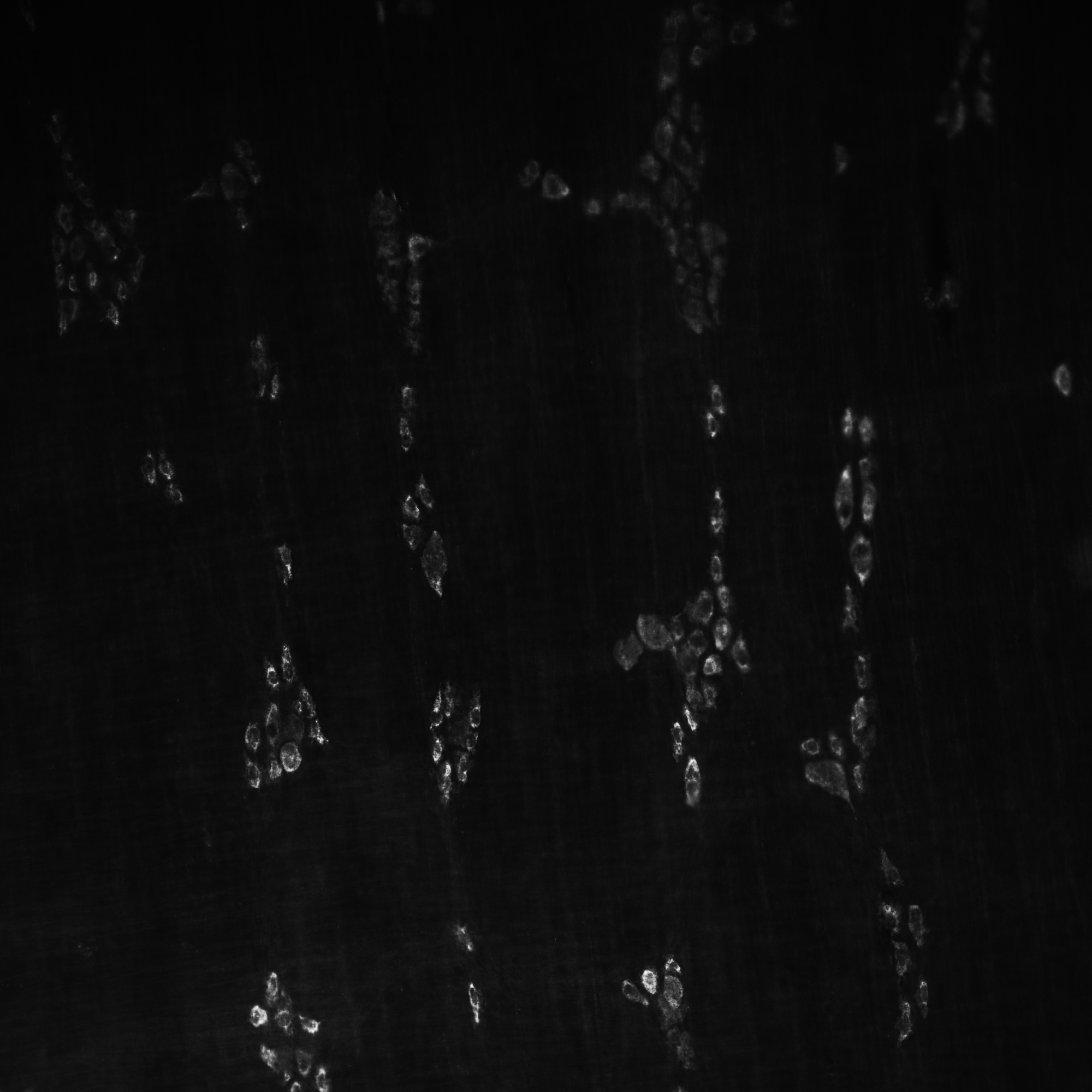

Supplement: Supplementary file 4 — Source data Fig. 2 [file 44321_2024_189_MOESM4_ESM.zip › 2/2d/Control.tif]

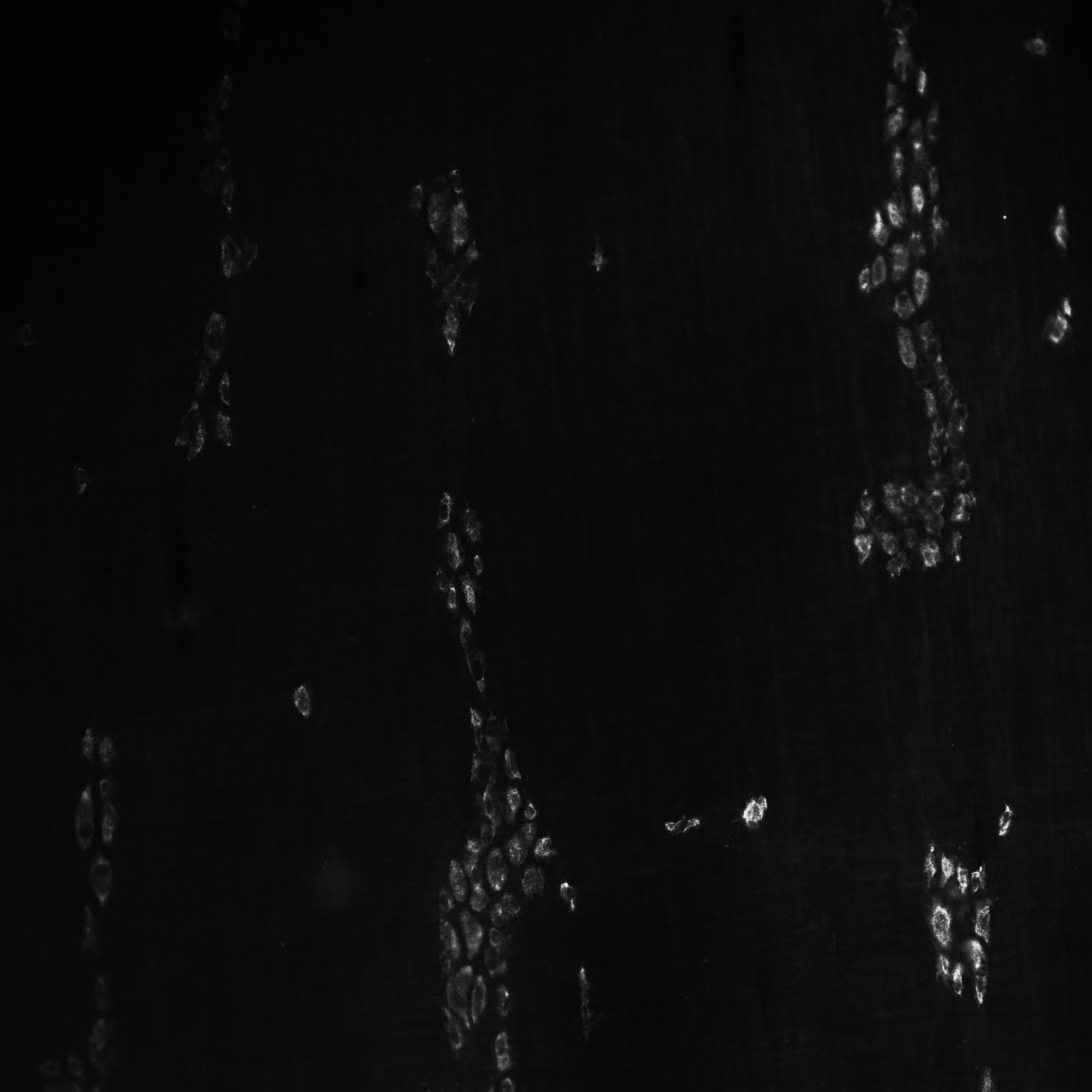

Supplement: Supplementary file 4 — Source data Fig. 2 [file 44321_2024_189_MOESM4_ESM.zip › 2/2d/IM3h.tif]

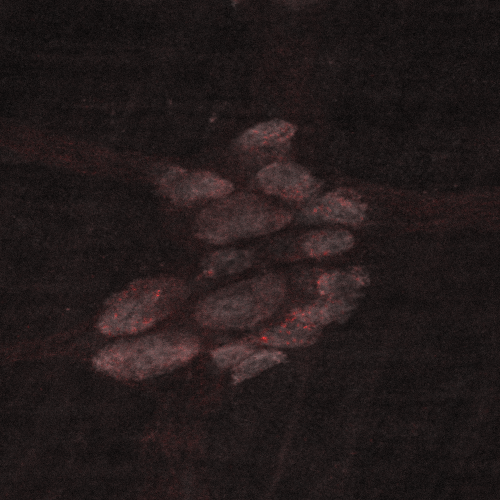

Supplement: Supplementary file 4 — Source data Fig. 2 [file 44321_2024_189_MOESM4_ESM.zip › 2/2e/Caspase3_Anna1_Control.tif]

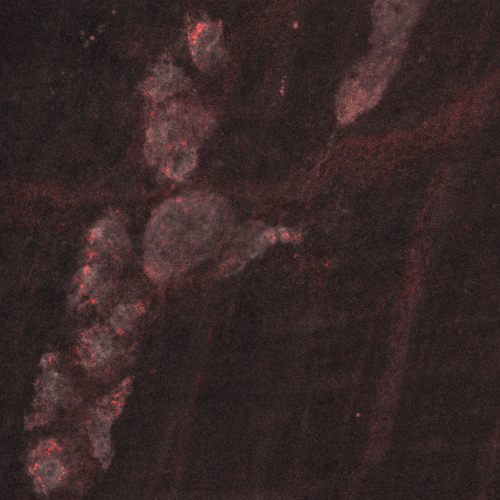

Supplement: Supplementary file 4 — Source data Fig. 2 [file 44321_2024_189_MOESM4_ESM.zip › 2/2e/Caspase3_Anna1_IM24h.tif]

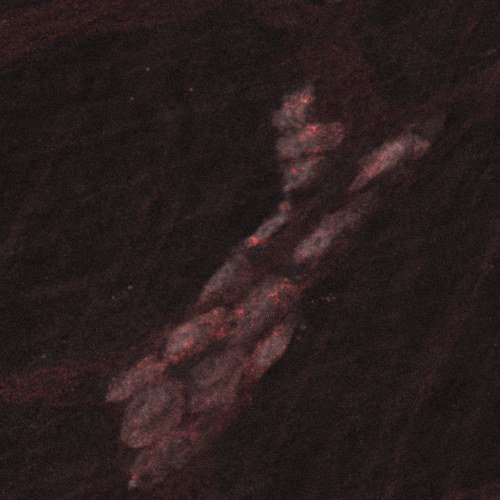

Supplement: Supplementary file 4 — Source data Fig. 2 [file 44321_2024_189_MOESM4_ESM.zip › 2/2e/Caspase3_Anna1_IM3h.tif]

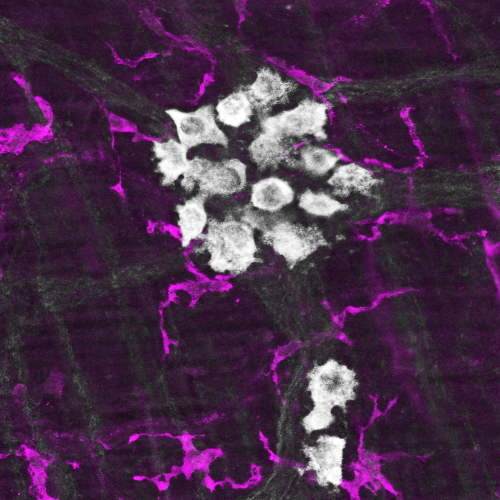

Supplement: Supplementary file 6 — Source data Fig. 4 [file 44321_2024_189_MOESM6_ESM.zip › 4/4c/IM24h.tif]

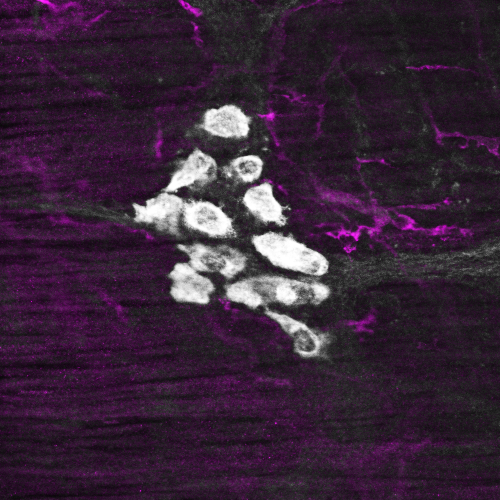

Supplement: Supplementary file 6 — Source data Fig. 4 [file 44321_2024_189_MOESM6_ESM.zip › 4/4c/Control.tif]

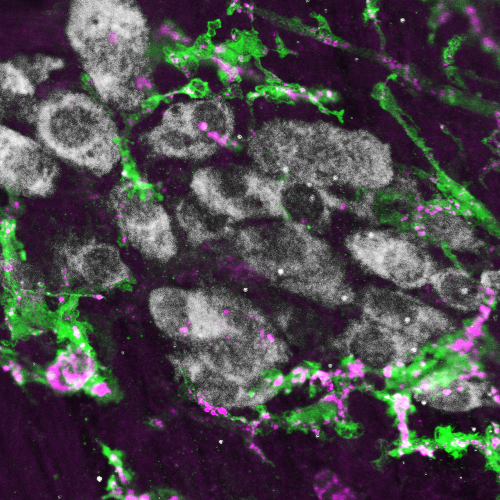

Supplement: Supplementary file 6 — Source data Fig. 4 [file 44321_2024_189_MOESM6_ESM.zip › 4/4d/Control.tif]

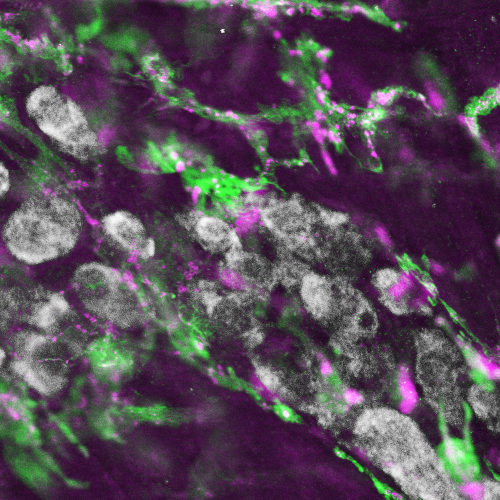

Supplement: Supplementary file 6 — Source data Fig. 4 [file 44321_2024_189_MOESM6_ESM.zip › 4/4d/IM3h.tif]

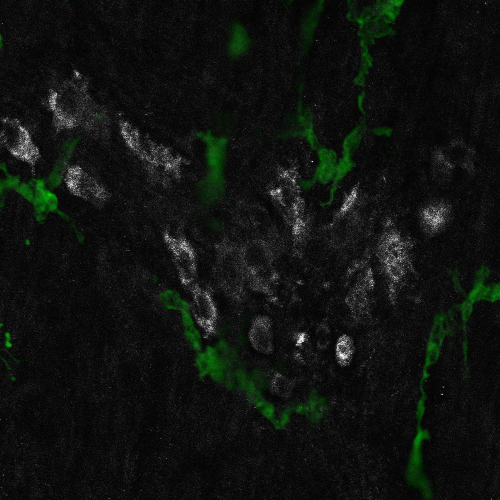

Supplement: Supplementary file 6 — Source data Fig. 4 [file 44321_2024_189_MOESM6_ESM.zip › 4/4a/IM24h_Cut.tif]

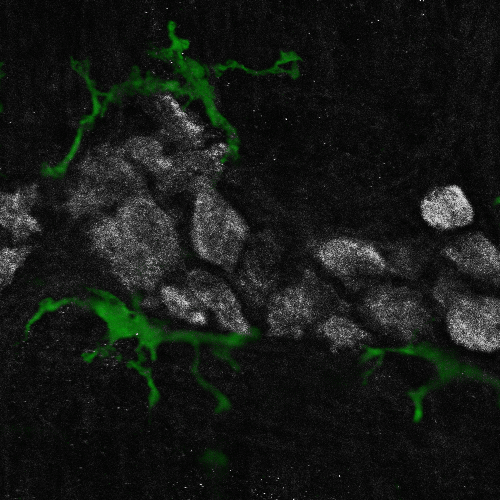

Supplement: Supplementary file 6 — Source data Fig. 4 [file 44321_2024_189_MOESM6_ESM.zip › 4/4a/Control_Cut.tif]

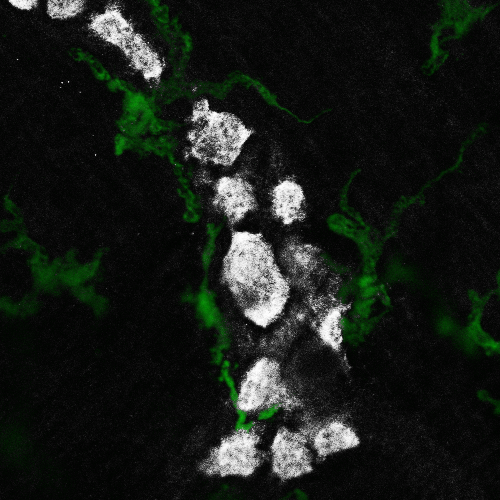

Supplement: Supplementary file 6 — Source data Fig. 4 [file 44321_2024_189_MOESM6_ESM.zip › 4/4a/IM3h_2_CUT.tif]

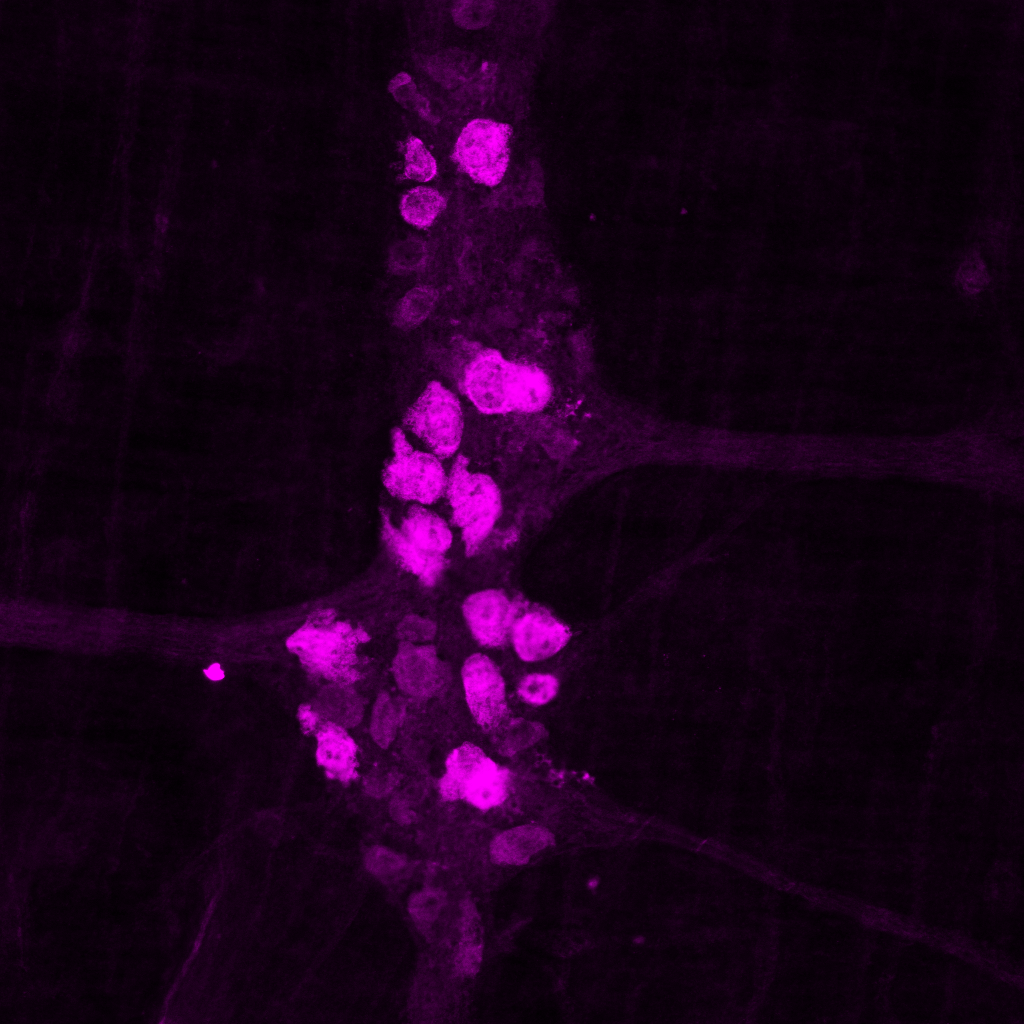

Supplement: Supplementary file 7 — Source data Fig. 5 [file 44321_2024_189_MOESM7_ESM.zip › 5/5f/CD115_IM24h.tif]

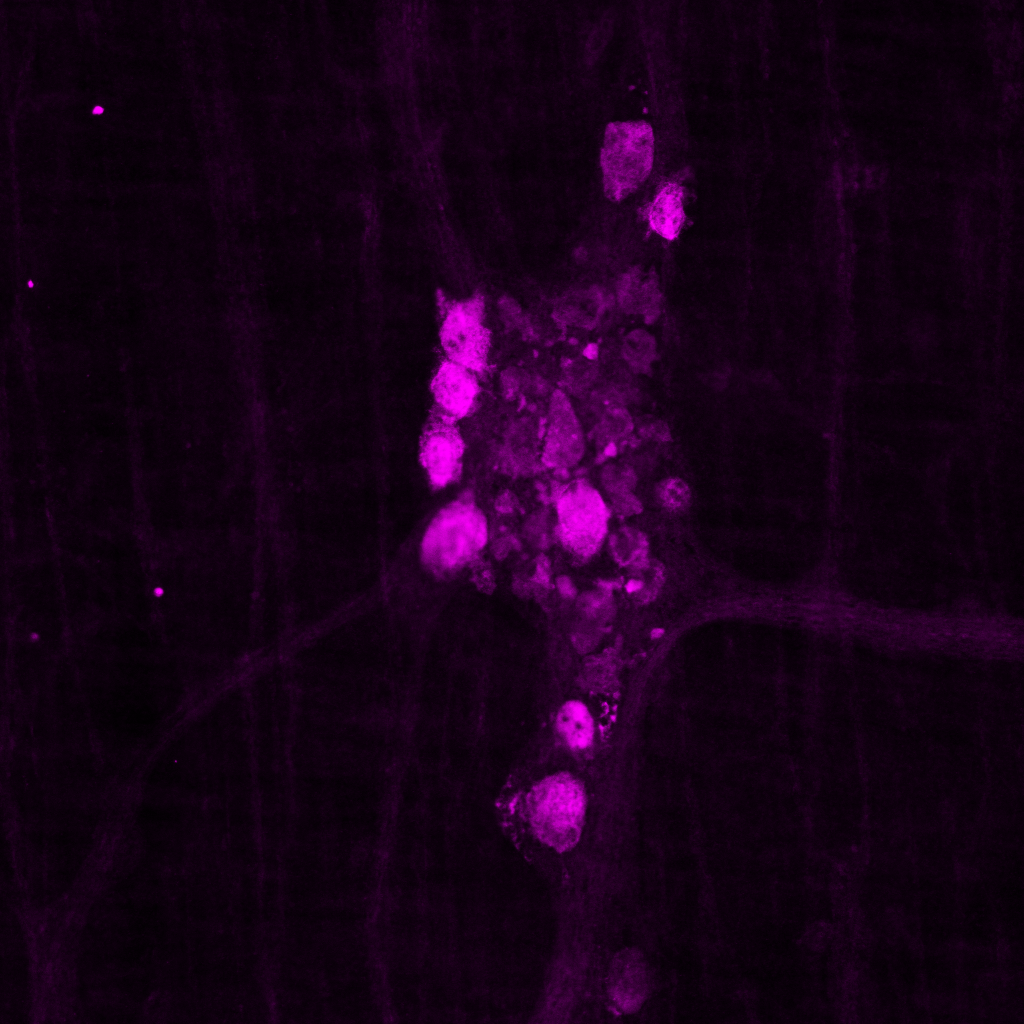

Supplement: Supplementary file 7 — Source data Fig. 5 [file 44321_2024_189_MOESM7_ESM.zip › 5/5f/IgG_IM24h.tif]

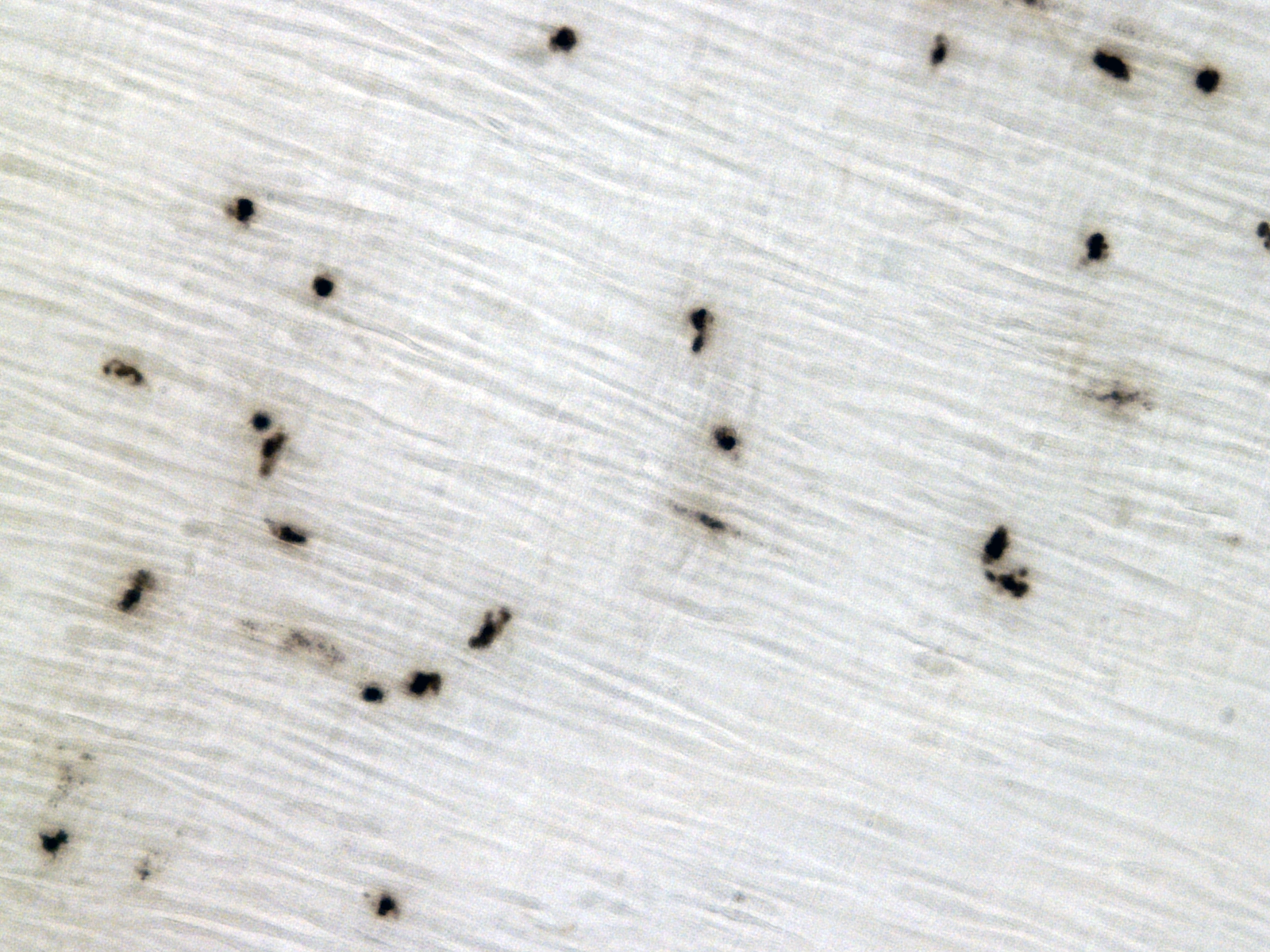

Supplement: Supplementary file 7 — Source data Fig. 5 [file 44321_2024_189_MOESM7_ESM.zip › 5/5d/Depl48h_IM24h_2.tif]

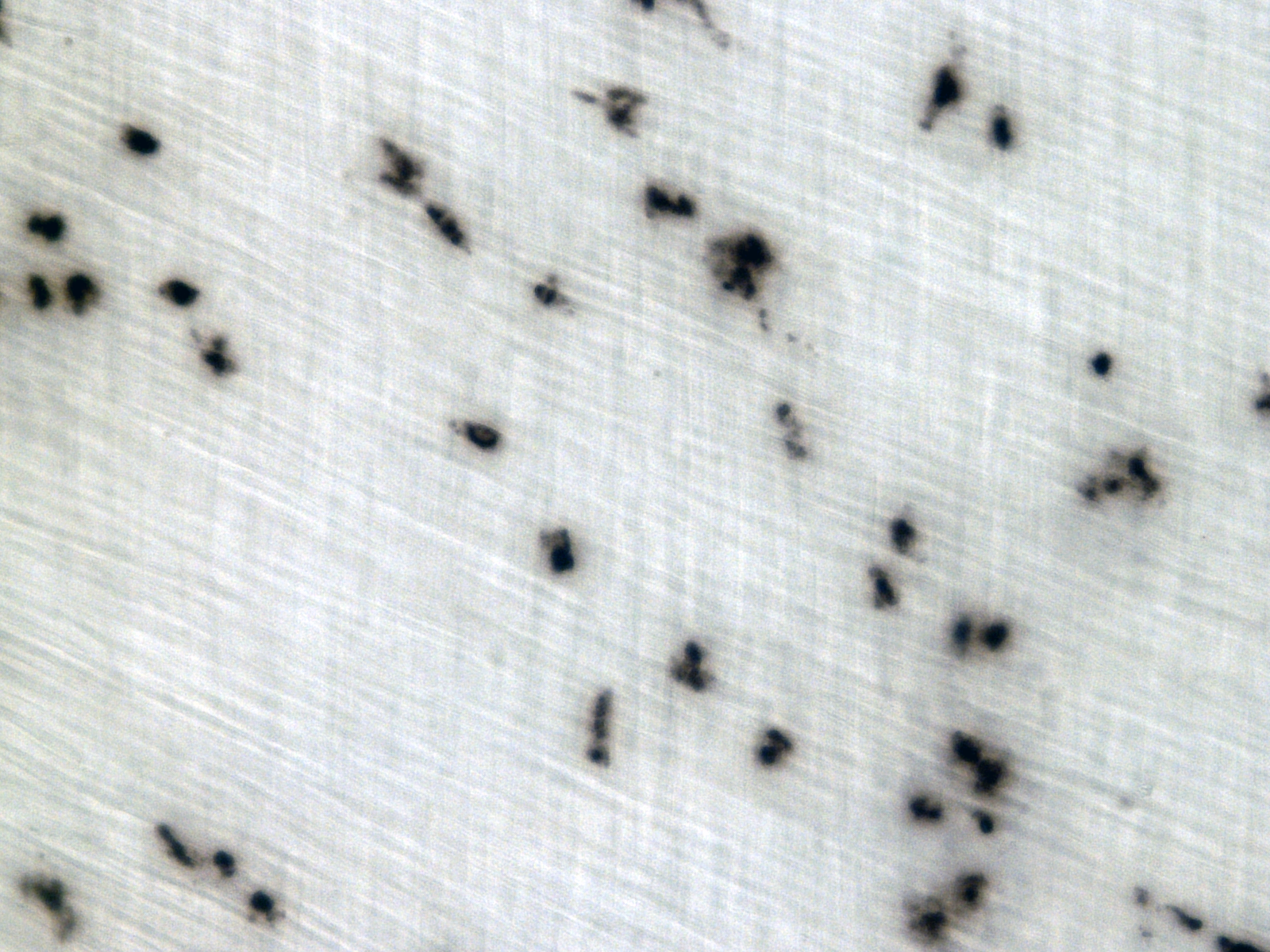

Supplement: Supplementary file 7 — Source data Fig. 5 [file 44321_2024_189_MOESM7_ESM.zip › 5/5d/rat IgG-IM24h_2.tif]

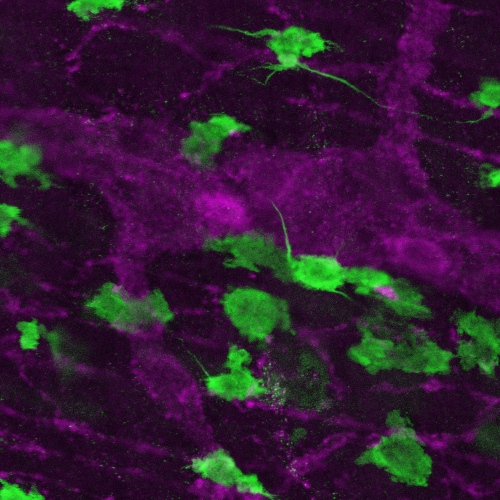

Supplement: Supplementary file 7 — Source data Fig. 5 [file 44321_2024_189_MOESM7_ESM.zip › 5/5b/IgG.tif]

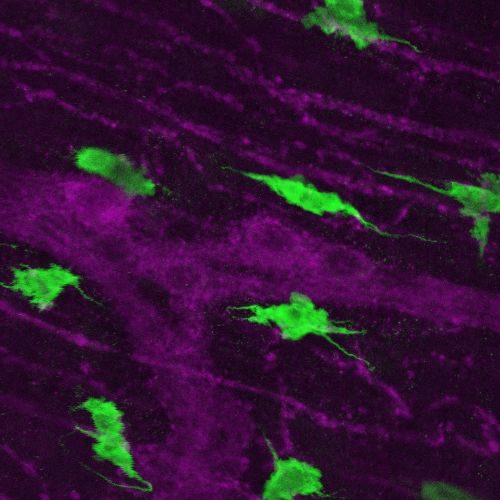

Supplement: Supplementary file 7 — Source data Fig. 5 [file 44321_2024_189_MOESM7_ESM.zip › 5/5b/CD115.tif]

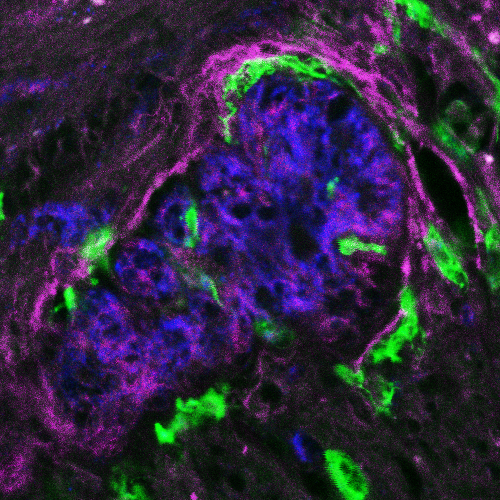

Supplement: Supplementary file 8 — Source data Fig. 6 [file 44321_2024_189_MOESM8_ESM.zip › 6/6b/cut1_all.tif]

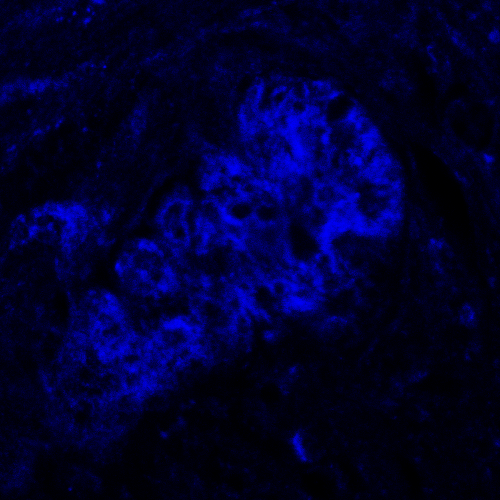

Supplement: Supplementary file 8 — Source data Fig. 6 [file 44321_2024_189_MOESM8_ESM.zip › 6/6b/cut1_cas3_blue.tif]

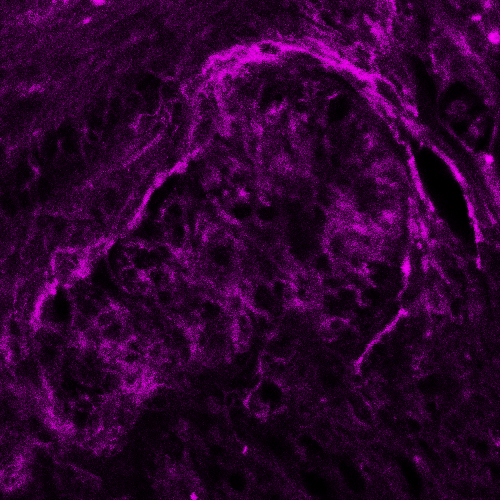

Supplement: Supplementary file 8 — Source data Fig. 6 [file 44321_2024_189_MOESM8_ESM.zip › 6/6b/cut1_chat.tif]

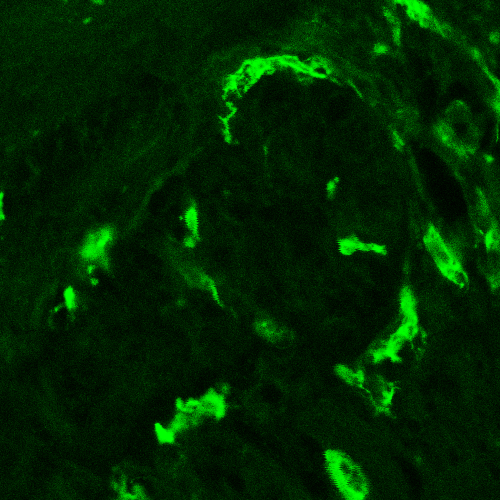

Supplement: Supplementary file 8 — Source data Fig. 6 [file 44321_2024_189_MOESM8_ESM.zip › 6/6b/cut1_green_iba1.tif]

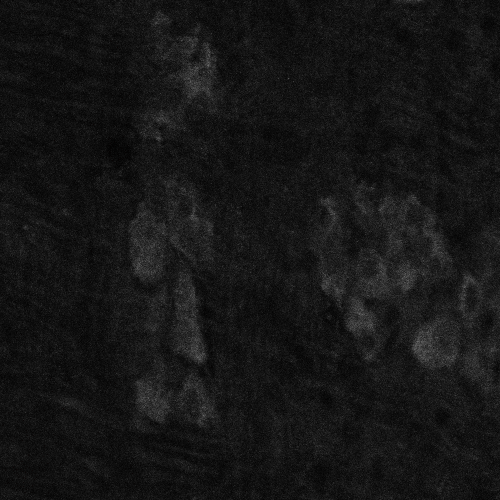

Supplement: Supplementary file 9 — Figure Source Data for EV and Appendix Figures [file 44321_2024_189_MOESM9_ESM.zip › EV and Appendix Figures/1/EVa/PSD95_FOS_Anna1.lif_Series024_ch02_overlay_Im24h_Suppl_CUT.tif]

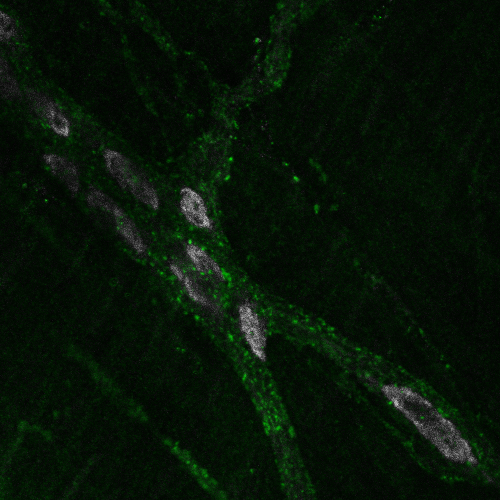

Supplement: Supplementary file 9 — Figure Source Data for EV and Appendix Figures [file 44321_2024_189_MOESM9_ESM.zip › EV and Appendix Figures/2/EVa/all_IM24h_2_Syn1_Anna1.tif]

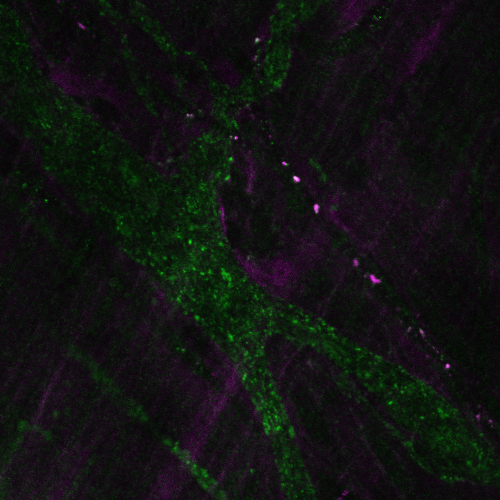

Supplement: Supplementary file 9 — Figure Source Data for EV and Appendix Figures [file 44321_2024_189_MOESM9_ESM.zip › EV and Appendix Figures/2/EVa/all_IM24h_2_Syn1_PSD95.tif]

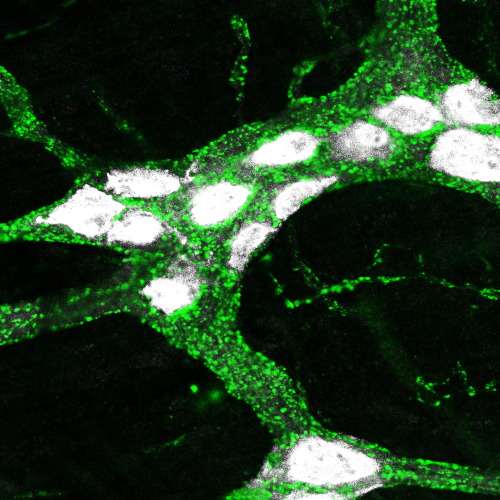

Supplement: Supplementary file 9 — Figure Source Data for EV and Appendix Figures [file 44321_2024_189_MOESM9_ESM.zip › EV and Appendix Figures/2/EVa/all_IM3h_syn1_Anna1.tif]

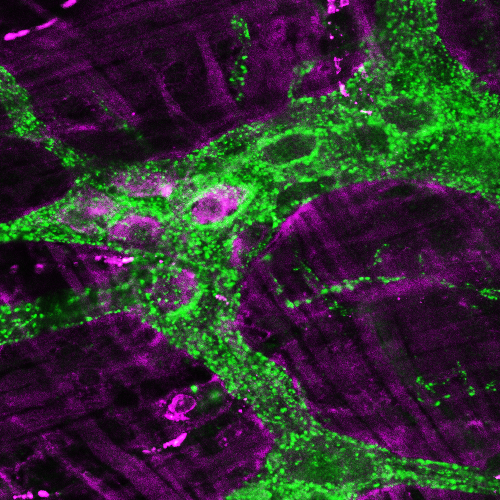

Supplement: Supplementary file 9 — Figure Source Data for EV and Appendix Figures [file 44321_2024_189_MOESM9_ESM.zip › EV and Appendix Figures/2/EVa/all_IM3h_syn1_PSD95.tif]

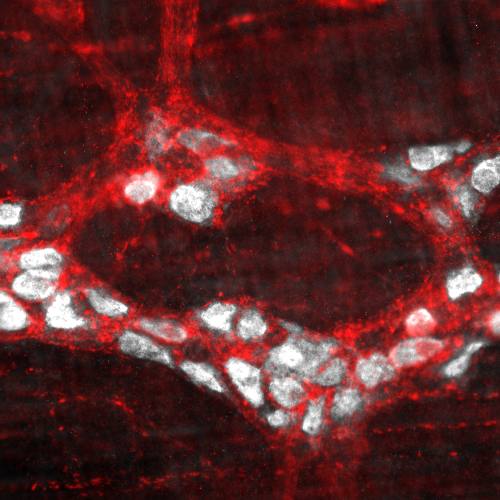

Supplement: Supplementary file 9 — Figure Source Data for EV and Appendix Figures [file 44321_2024_189_MOESM9_ESM.zip › EV and Appendix Figures/2/EVa/Chat-Control.tif]

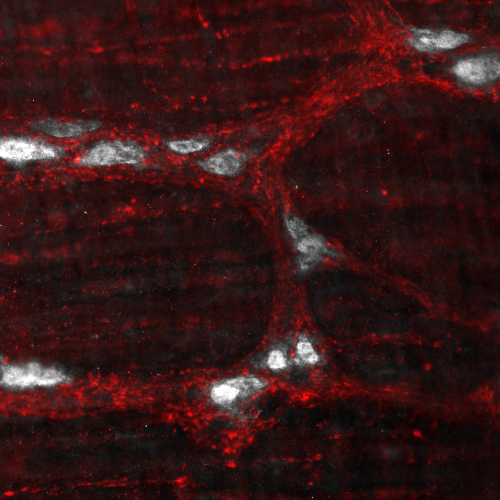

Supplement: Supplementary file 9 — Figure Source Data for EV and Appendix Figures [file 44321_2024_189_MOESM9_ESM.zip › EV and Appendix Figures/2/EVa/Chat-IM24h.tif]

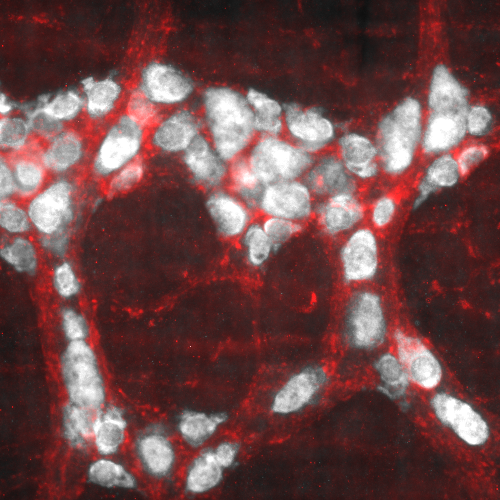

Supplement: Supplementary file 9 — Figure Source Data for EV and Appendix Figures [file 44321_2024_189_MOESM9_ESM.zip › EV and Appendix Figures/2/EVa/Chat-IM3h.tif]

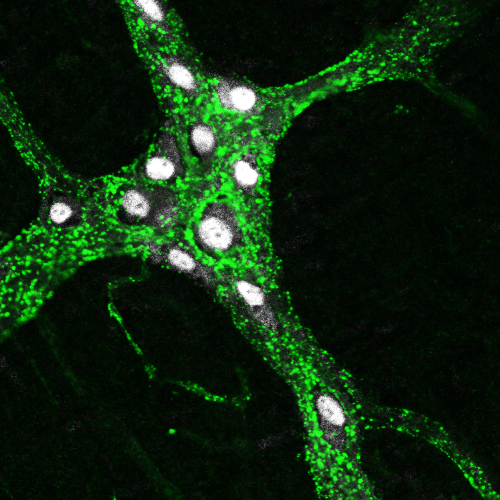

Supplement: Supplementary file 9 — Figure Source Data for EV and Appendix Figures [file 44321_2024_189_MOESM9_ESM.zip › EV and Appendix Figures/2/EVa/Control_cut2_syn1_Anna1.tif]

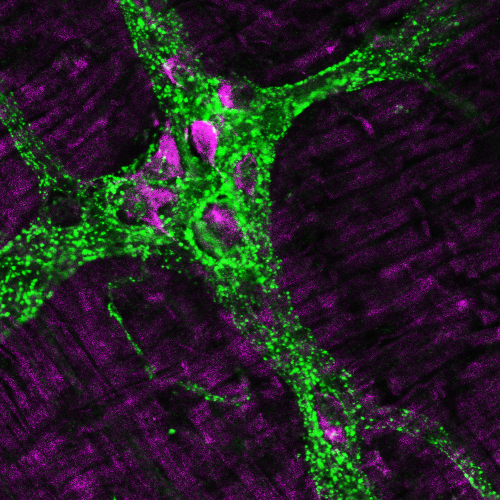

Supplement: Supplementary file 9 — Figure Source Data for EV and Appendix Figures [file 44321_2024_189_MOESM9_ESM.zip › EV and Appendix Figures/2/EVa/Control_cut2_syn1_PSD95.tif]

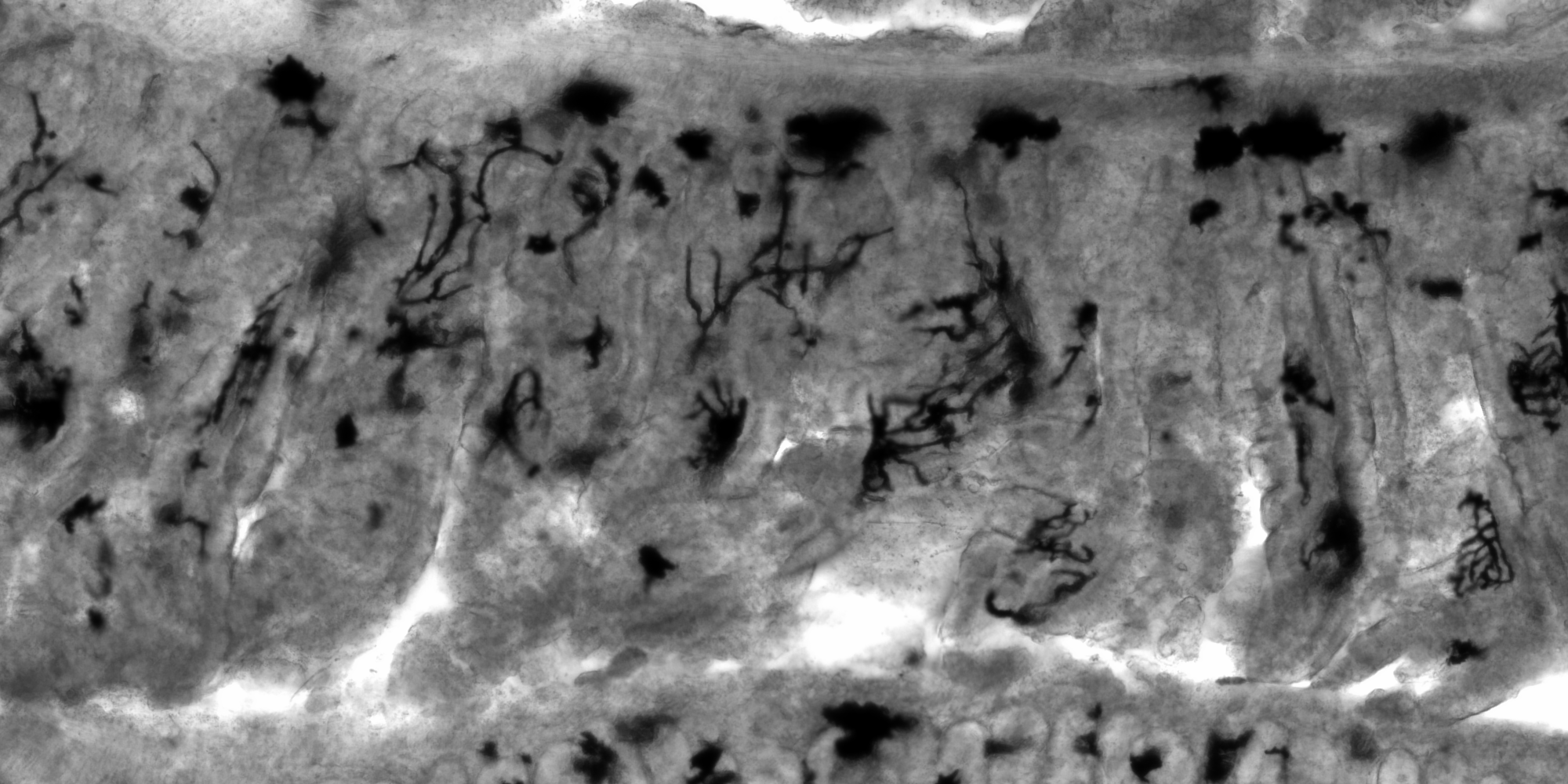

Supplement: Supplementary file 9 — Figure Source Data for EV and Appendix Figures [file 44321_2024_189_MOESM9_ESM.zip › EV and Appendix Figures/2/EVb/Control.tif]

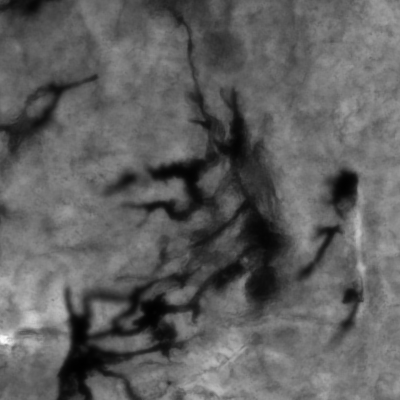

Supplement: Supplementary file 9 — Figure Source Data for EV and Appendix Figures [file 44321_2024_189_MOESM9_ESM.zip › EV and Appendix Figures/2/EVb/Control_CUT.tif]

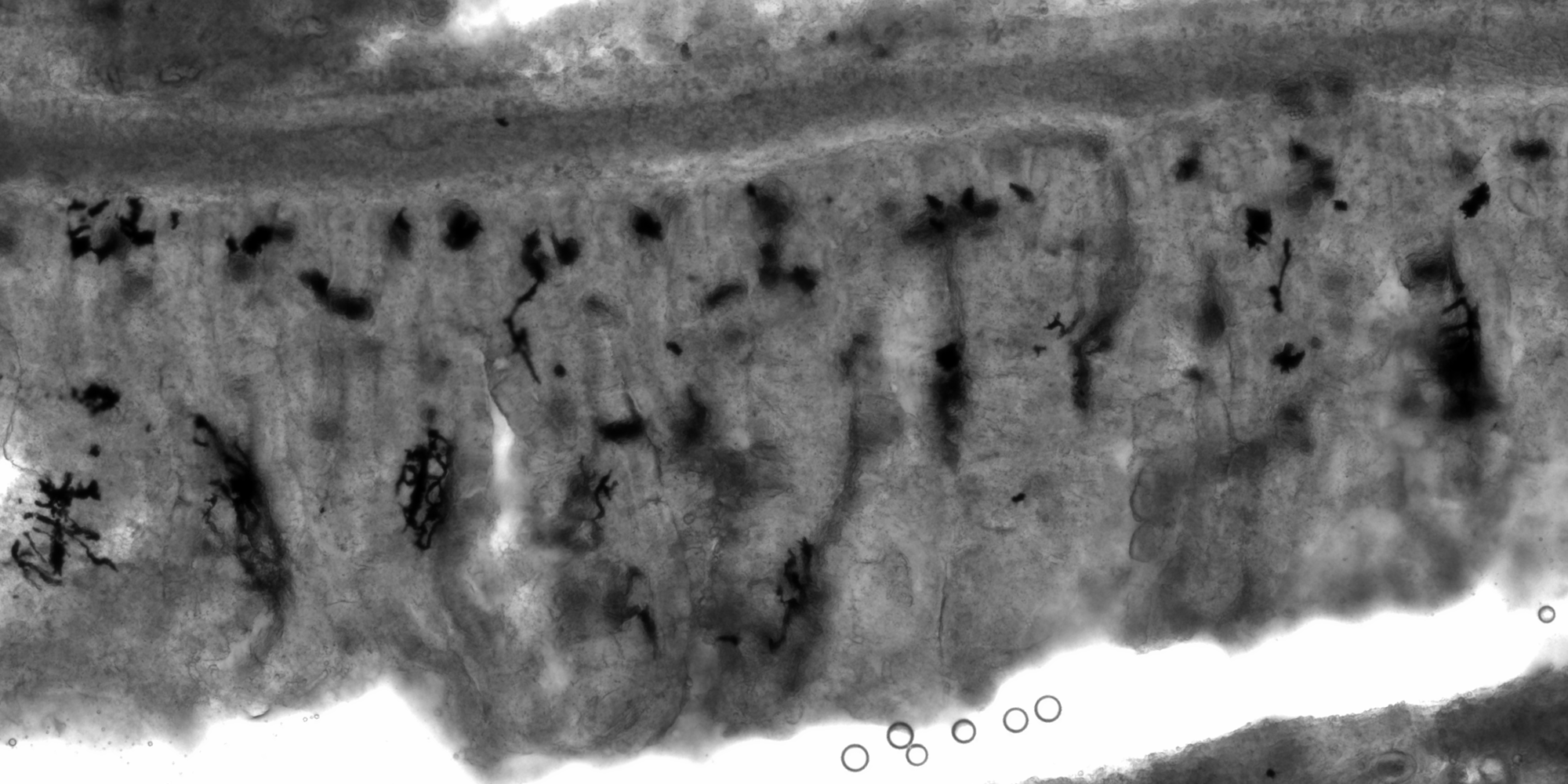

Supplement: Supplementary file 9 — Figure Source Data for EV and Appendix Figures [file 44321_2024_189_MOESM9_ESM.zip › EV and Appendix Figures/2/EVb/IM24h.tif]

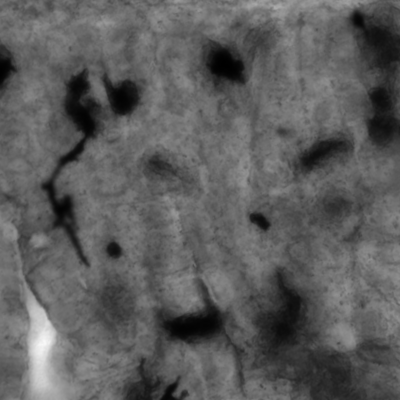

Supplement: Supplementary file 9 — Figure Source Data for EV and Appendix Figures [file 44321_2024_189_MOESM9_ESM.zip › EV and Appendix Figures/2/EVb/IM24h_CUT.tif]

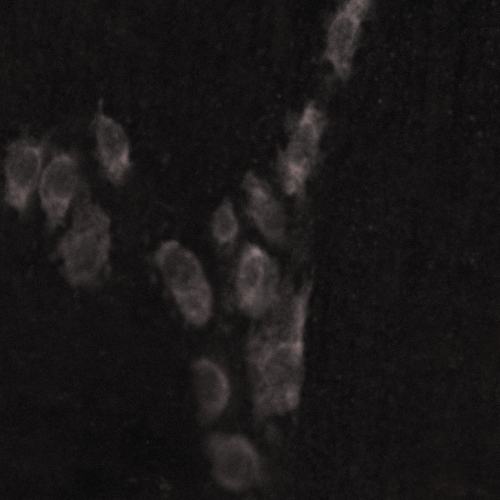

Supplement: Supplementary file 9 — Figure Source Data for EV and Appendix Figures [file 44321_2024_189_MOESM9_ESM.zip › EV and Appendix Figures/2/EVe/control.tif]

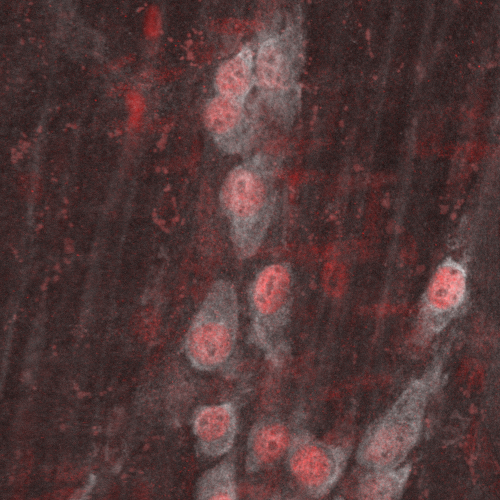

Supplement: Supplementary file 9 — Figure Source Data for EV and Appendix Figures [file 44321_2024_189_MOESM9_ESM.zip › EV and Appendix Figures/2/EVe/Ki67_Anna1_IM24h.tif]

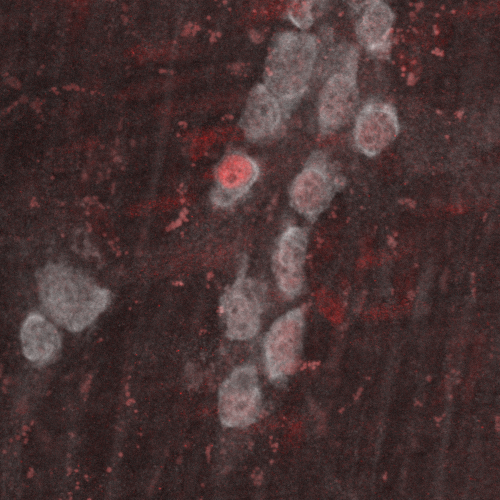

Supplement: Supplementary file 9 — Figure Source Data for EV and Appendix Figures [file 44321_2024_189_MOESM9_ESM.zip › EV and Appendix Figures/2/EVe/Ki67_Anna1_IM3h.tif]

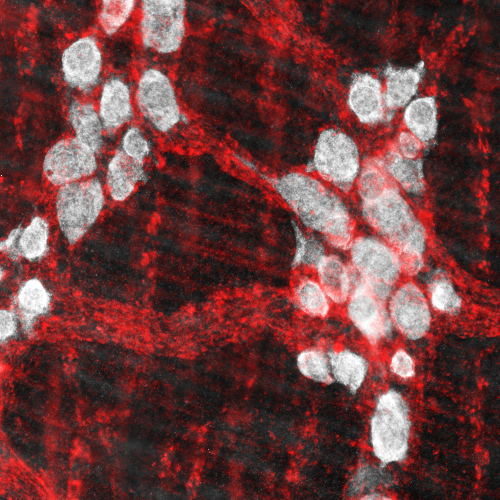

Supplement: Supplementary file 9 — Figure Source Data for EV and Appendix Figures [file 44321_2024_189_MOESM9_ESM.zip › EV and Appendix Figures/3/EVa/Chat_anna1_.tif]

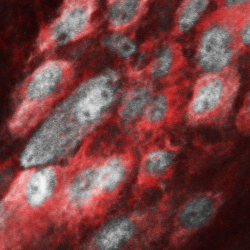

Supplement: Supplementary file 9 — Figure Source Data for EV and Appendix Figures [file 44321_2024_189_MOESM9_ESM.zip › EV and Appendix Figures/3/EVa/Control.tif]

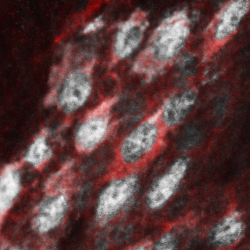

Supplement: Supplementary file 9 — Figure Source Data for EV and Appendix Figures [file 44321_2024_189_MOESM9_ESM.zip › EV and Appendix Figures/3/EVa/IM24h.tif]

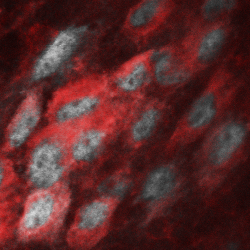

Supplement: Supplementary file 9 — Figure Source Data for EV and Appendix Figures [file 44321_2024_189_MOESM9_ESM.zip › EV and Appendix Figures/3/EVa/IM3h.tif]

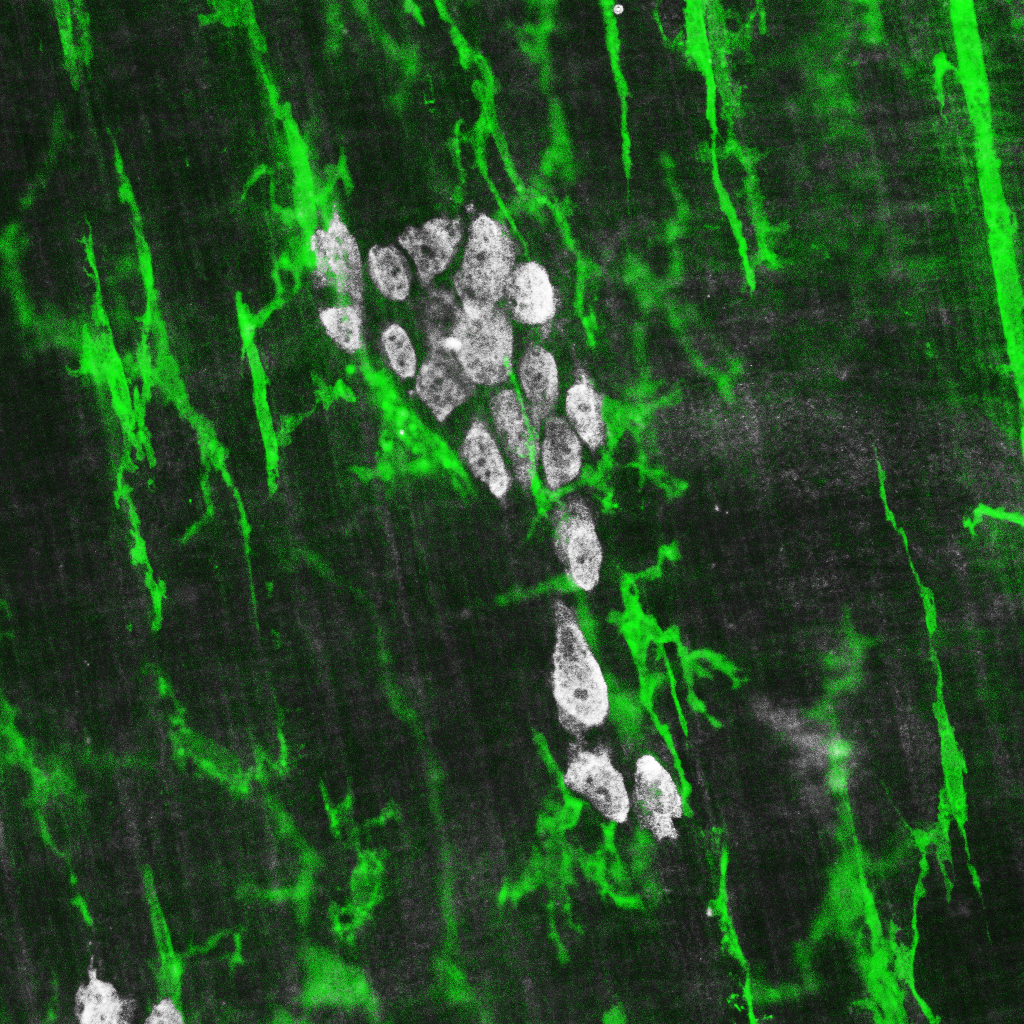

Supplement: Supplementary file 9 — Figure Source Data for EV and Appendix Figures [file 44321_2024_189_MOESM9_ESM.zip › EV and Appendix Figures/4/EVa/CX3CR1-GFP ANNA1.tif]

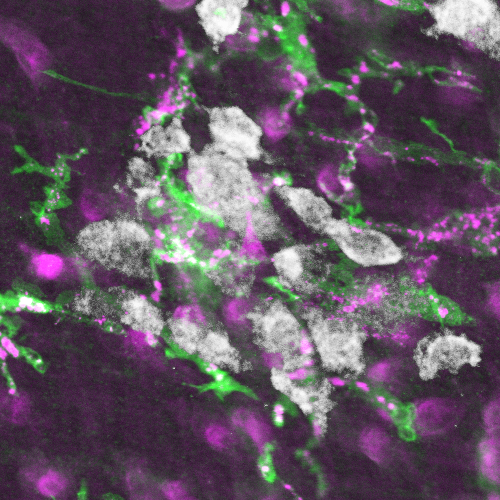

Supplement: Supplementary file 9 — Figure Source Data for EV and Appendix Figures [file 44321_2024_189_MOESM9_ESM.zip › EV and Appendix Figures/4/EVb/IM24h.tif]

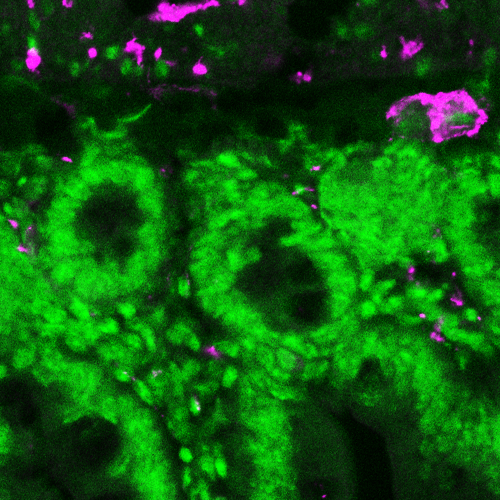

Supplement: Supplementary file 9 — Figure Source Data for EV and Appendix Figures [file 44321_2024_189_MOESM9_ESM.zip › EV and Appendix Figures/4/EVl/CCR2-KO.tif]

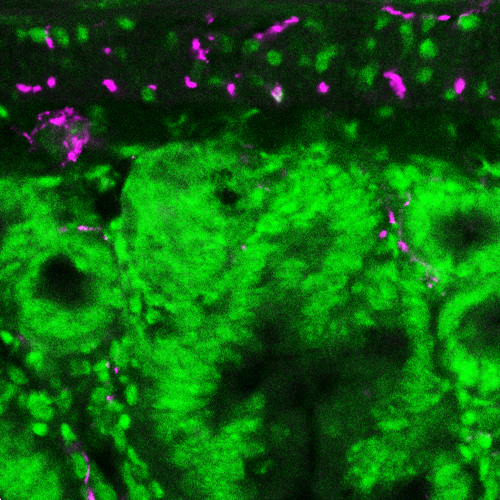

Supplement: Supplementary file 9 — Figure Source Data for EV and Appendix Figures [file 44321_2024_189_MOESM9_ESM.zip › EV and Appendix Figures/4/EVl/WT.tif]

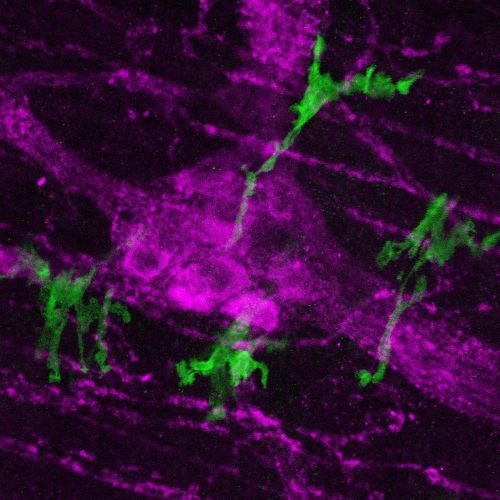

Supplement: Supplementary file 9 — Figure Source Data for EV and Appendix Figures [file 44321_2024_189_MOESM9_ESM.zip › EV and Appendix Figures/5/EVa/CD115.tif]

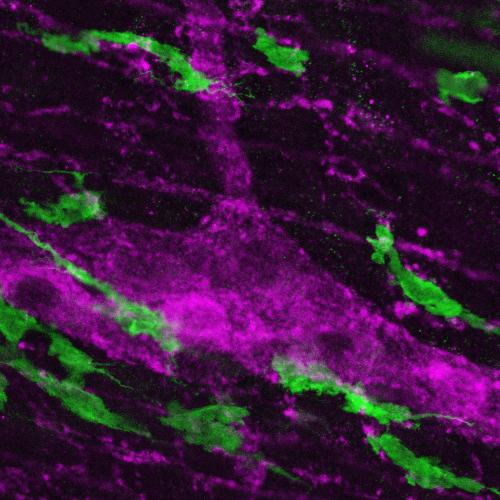

Supplement: Supplementary file 9 — Figure Source Data for EV and Appendix Figures [file 44321_2024_189_MOESM9_ESM.zip › EV and Appendix Figures/5/EVa/IgG.tif]

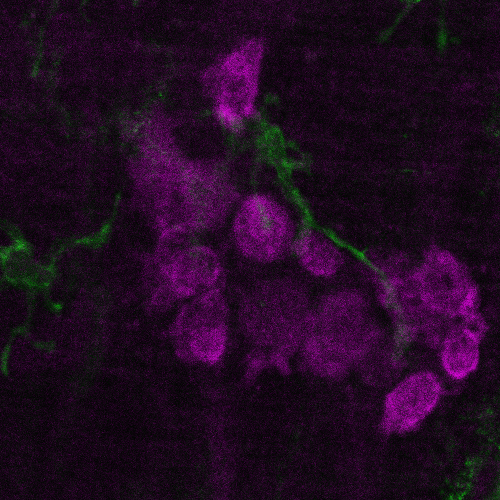

Supplement: Supplementary file 9 — Figure Source Data for EV and Appendix Figures [file 44321_2024_189_MOESM9_ESM.zip › EV and Appendix Figures/5/EVe/CD115_IM24h.tif]

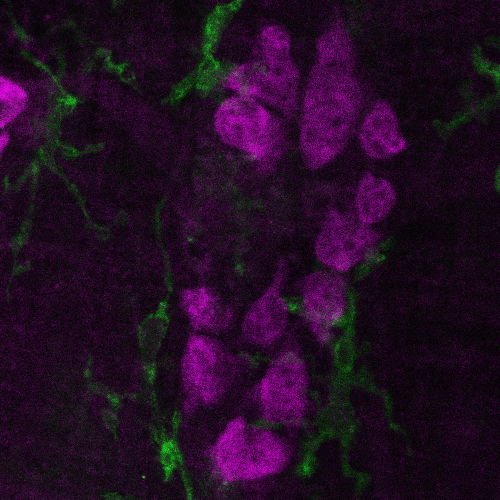

Supplement: Supplementary file 9 — Figure Source Data for EV and Appendix Figures [file 44321_2024_189_MOESM9_ESM.zip › EV and Appendix Figures/5/EVe/IgG_IM24h.tif]

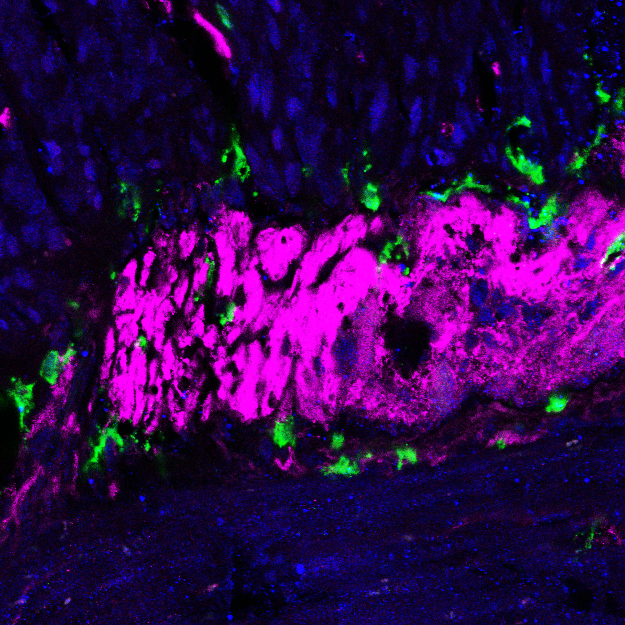

Supplement: Supplementary file 9 — Figure Source Data for EV and Appendix Figures [file 44321_2024_189_MOESM9_ESM.zip › EV and Appendix Figures/6/EVa/Cut_Merge.tif]

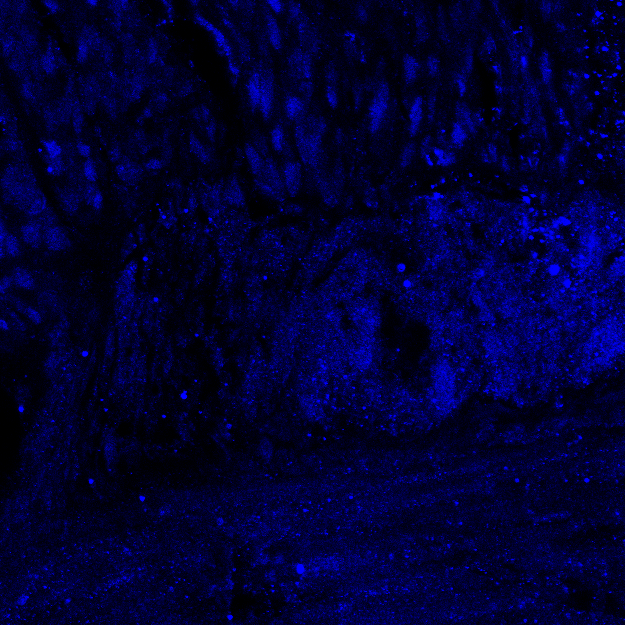

Supplement: Supplementary file 9 — Figure Source Data for EV and Appendix Figures [file 44321_2024_189_MOESM9_ESM.zip › EV and Appendix Figures/6/EVa/Cut_Merge_cFOS.tif]

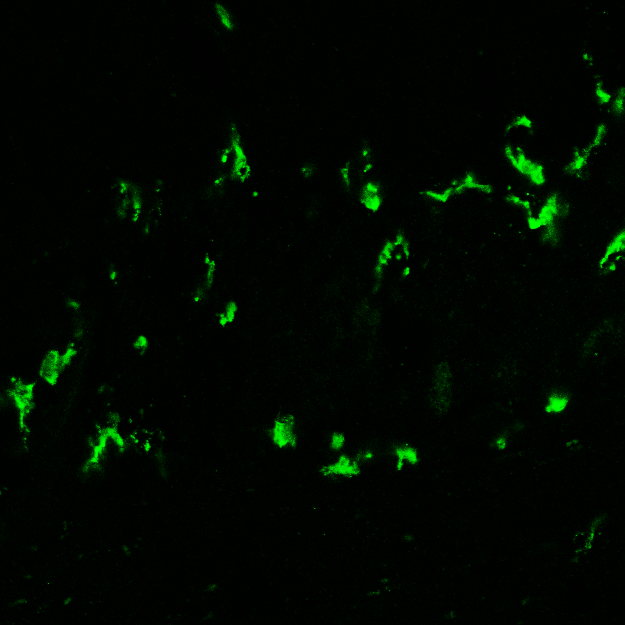

Supplement: Supplementary file 9 — Figure Source Data for EV and Appendix Figures [file 44321_2024_189_MOESM9_ESM.zip › EV and Appendix Figures/6/EVa/Cut_Merge_IBA1.tif]

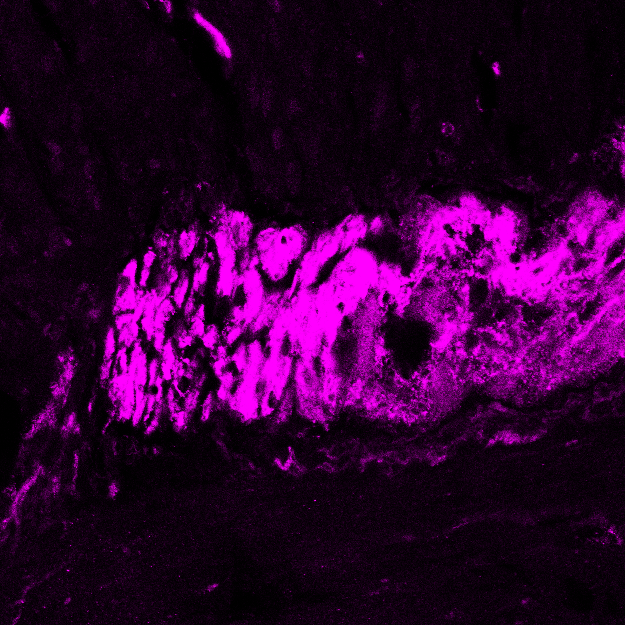

Supplement: Supplementary file 9 — Figure Source Data for EV and Appendix Figures [file 44321_2024_189_MOESM9_ESM.zip › EV and Appendix Figures/6/EVa/Cut_Merge_tubb3.tif]

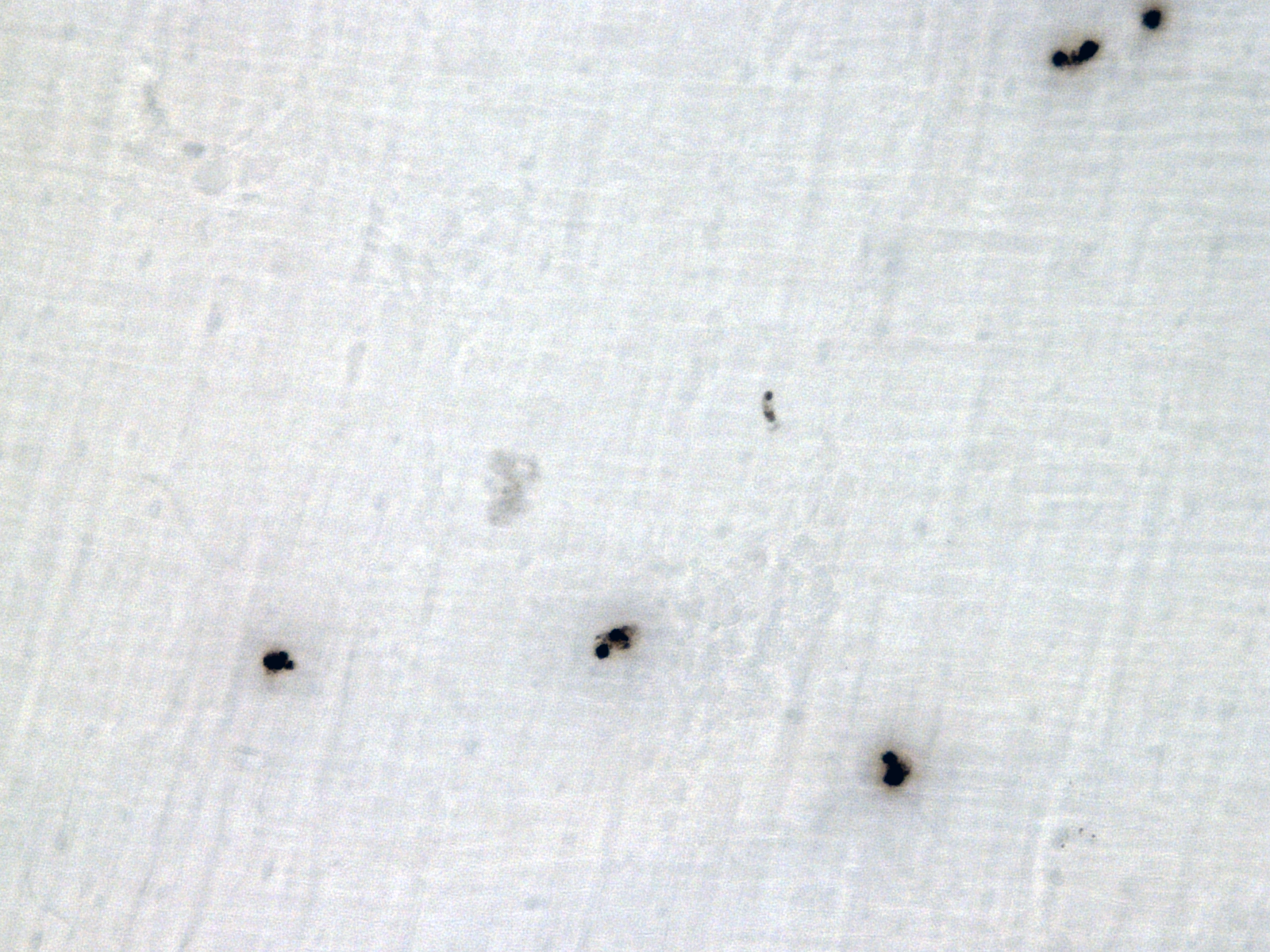

Supplement: Supplementary file 9 — Figure Source Data for EV and Appendix Figures [file 44321_2024_189_MOESM9_ESM.zip › EV and Appendix Figures/Appendix Figures/1/b/Control_2.tif]

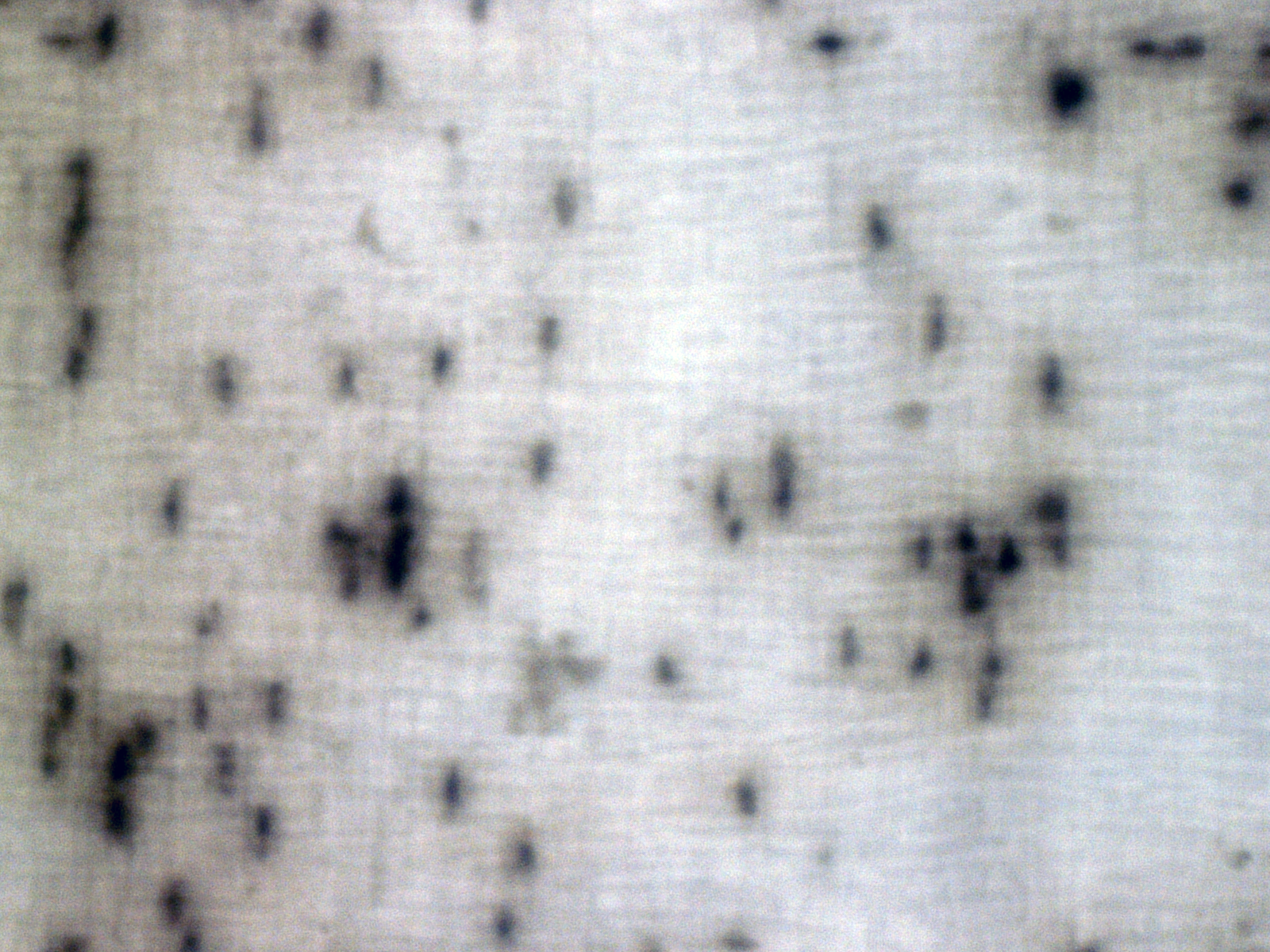

Supplement: Supplementary file 9 — Figure Source Data for EV and Appendix Figures [file 44321_2024_189_MOESM9_ESM.zip › EV and Appendix Figures/Appendix Figures/1/b/IM24h_2.tif]

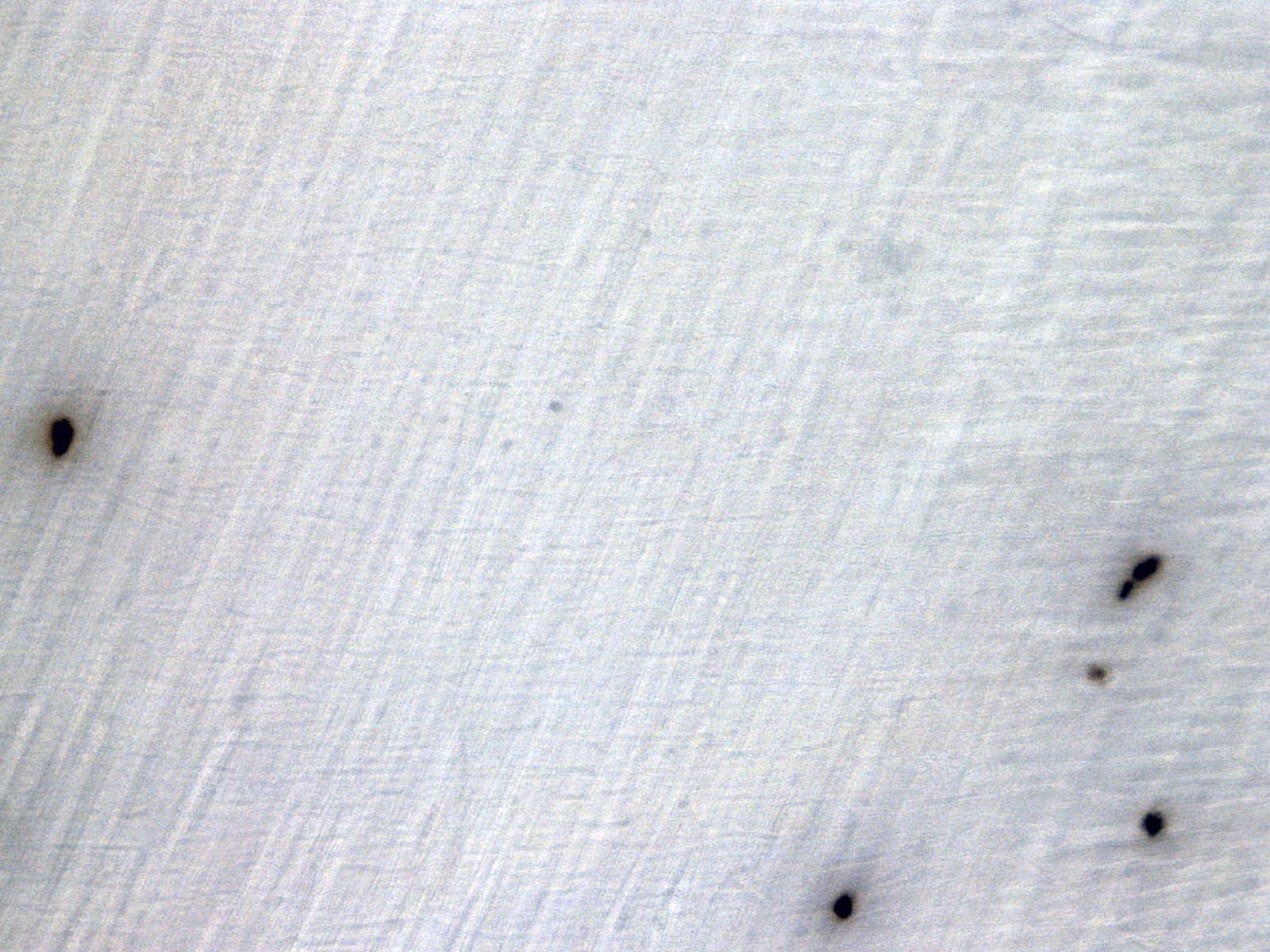

Supplement: Supplementary file 9 — Figure Source Data for EV and Appendix Figures [file 44321_2024_189_MOESM9_ESM.zip › EV and Appendix Figures/Appendix Figures/1/b/IM3h.tif]

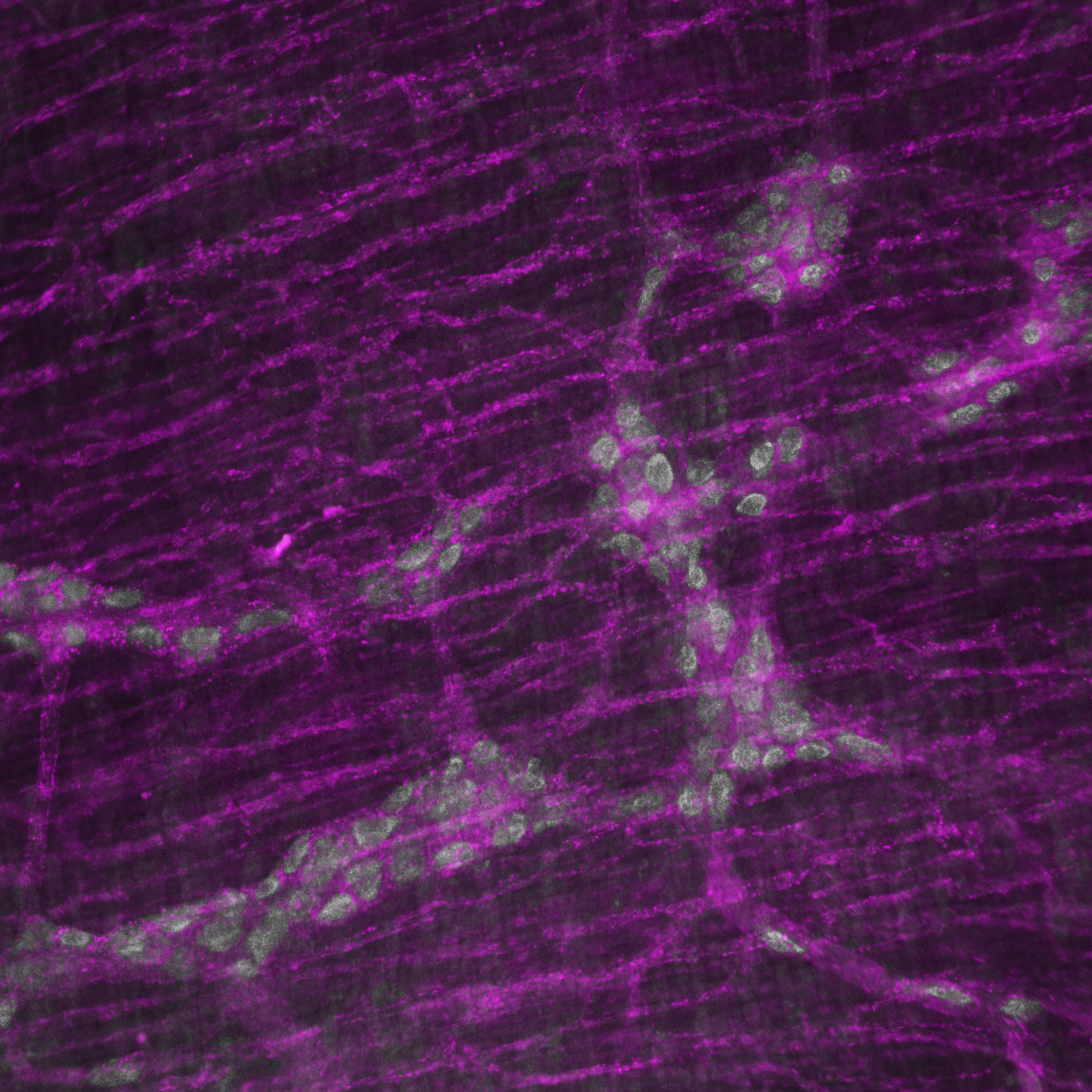

Supplement: Supplementary file 9 — Figure Source Data for EV and Appendix Figures [file 44321_2024_189_MOESM9_ESM.zip › EV and Appendix Figures/Appendix Figures/2/a/Control.tif]

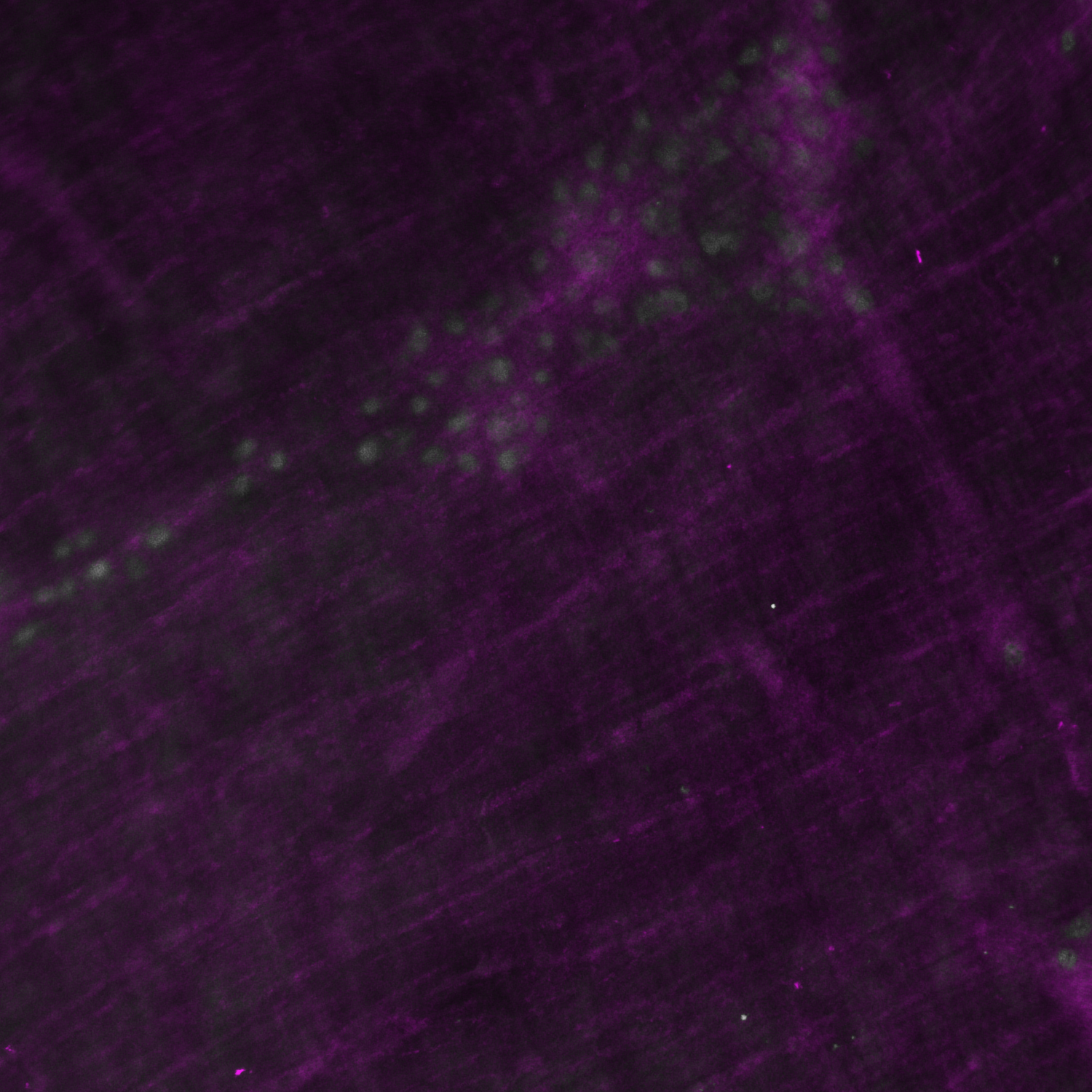

Supplement: Supplementary file 9 — Figure Source Data for EV and Appendix Figures [file 44321_2024_189_MOESM9_ESM.zip › EV and Appendix Figures/Appendix Figures/2/a/IM24h.tif]

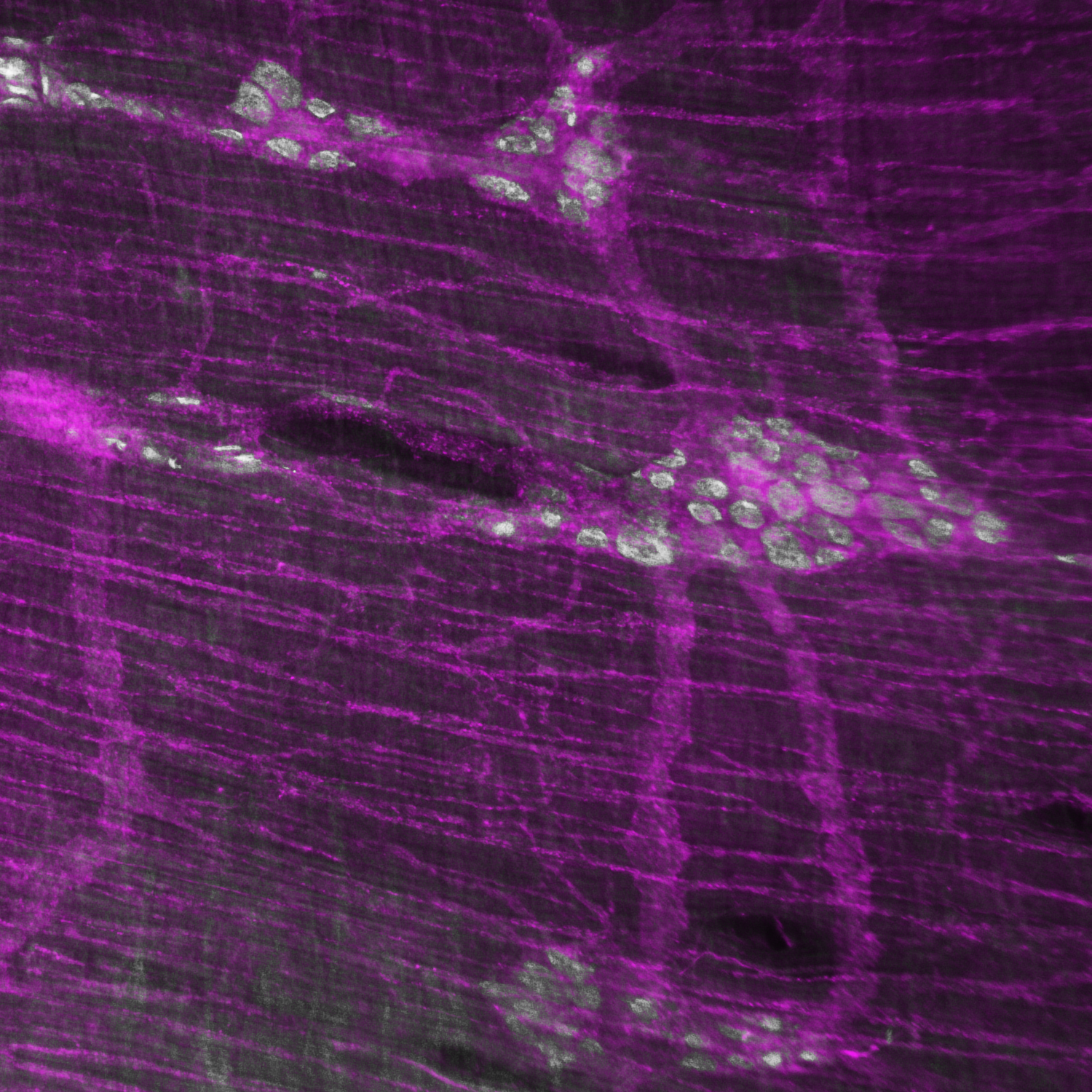

Supplement: Supplementary file 9 — Figure Source Data for EV and Appendix Figures [file 44321_2024_189_MOESM9_ESM.zip › EV and Appendix Figures/Appendix Figures/2/a/IM3h.tif]

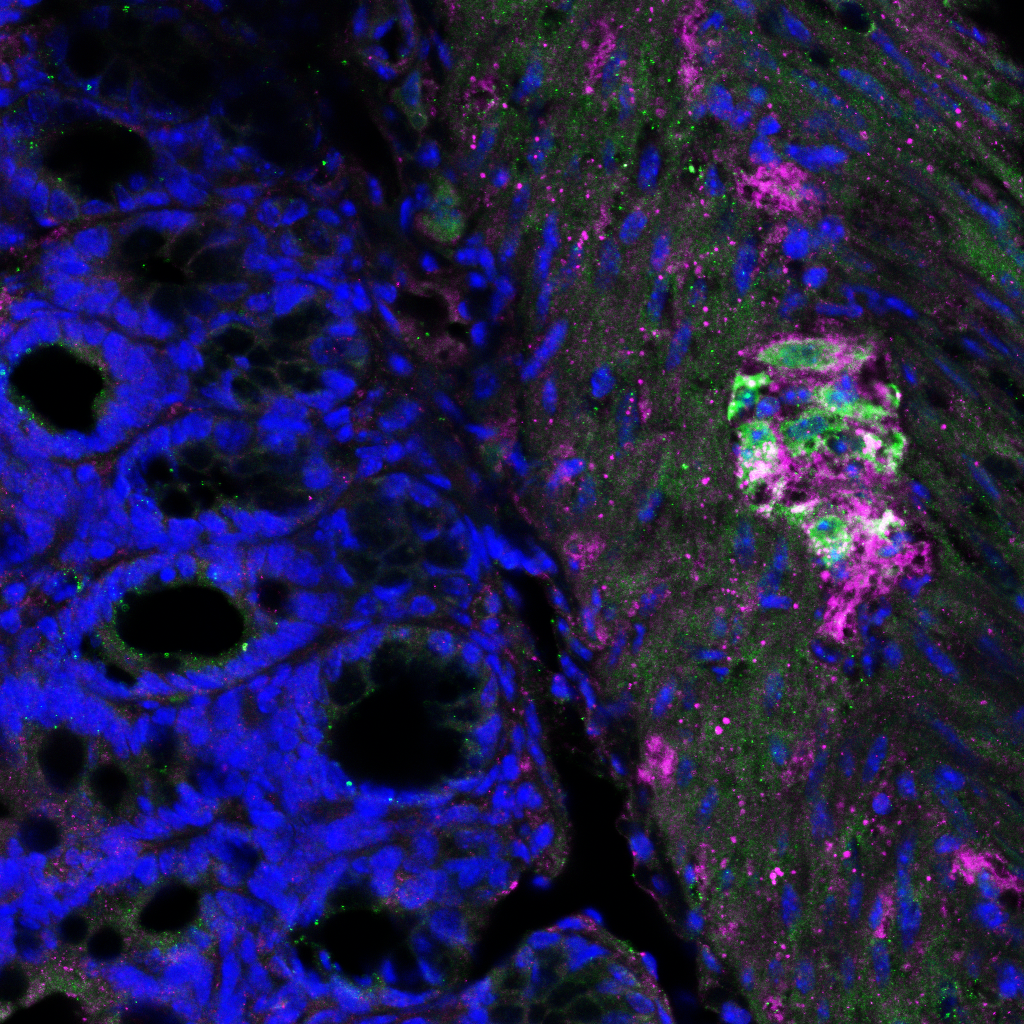

Supplement: Supplementary file 9 — Figure Source Data for EV and Appendix Figures [file 44321_2024_189_MOESM9_ESM.zip › EV and Appendix Figures/Appendix Figures/2/b/Cryo_IM24h.tif]

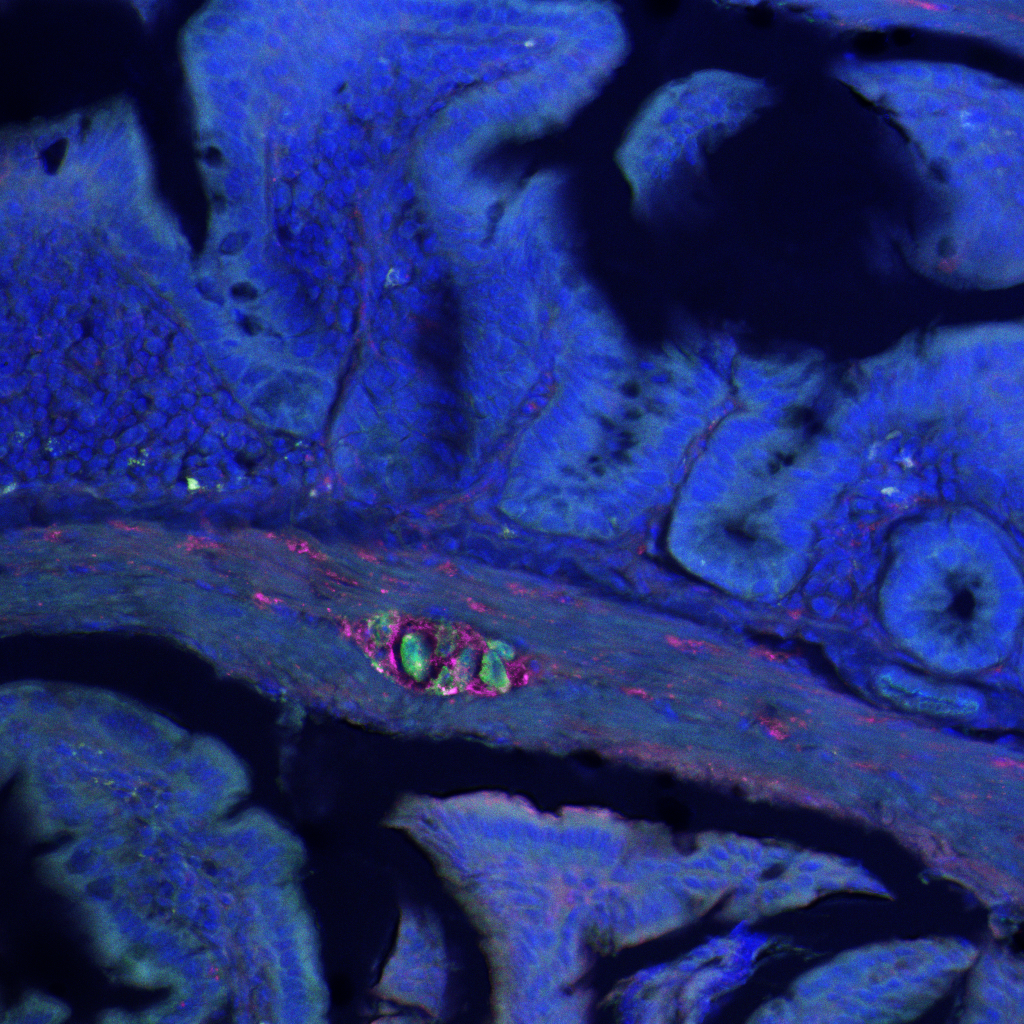

Supplement: Supplementary file 9 — Figure Source Data for EV and Appendix Figures [file 44321_2024_189_MOESM9_ESM.zip › EV and Appendix Figures/Appendix Figures/2/b/Parrafin_IM24h.tif]

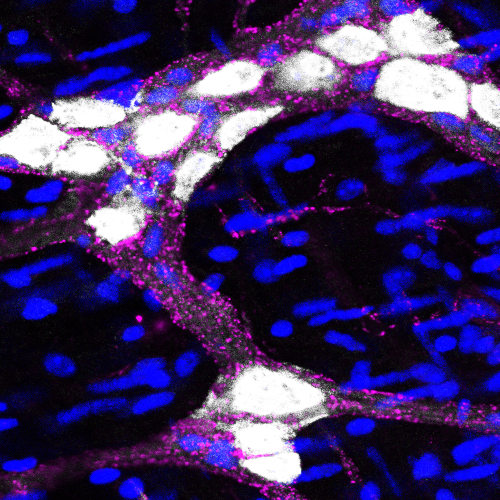

Supplement: Supplementary file 9 — Figure Source Data for EV and Appendix Figures [file 44321_2024_189_MOESM9_ESM.zip › EV and Appendix Figures/Appendix Figures/2/c/Control.tif]

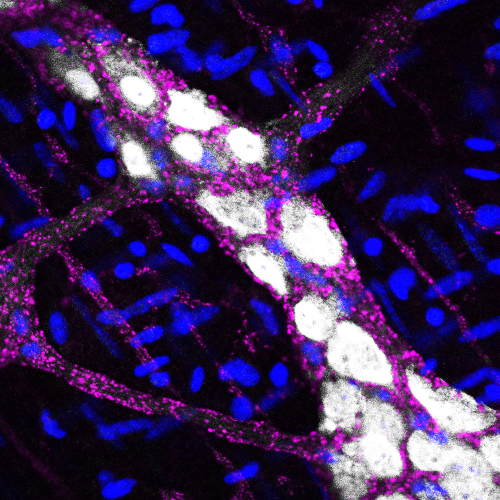

Supplement: Supplementary file 9 — Figure Source Data for EV and Appendix Figures [file 44321_2024_189_MOESM9_ESM.zip › EV and Appendix Figures/Appendix Figures/2/c/IM21days.tif]

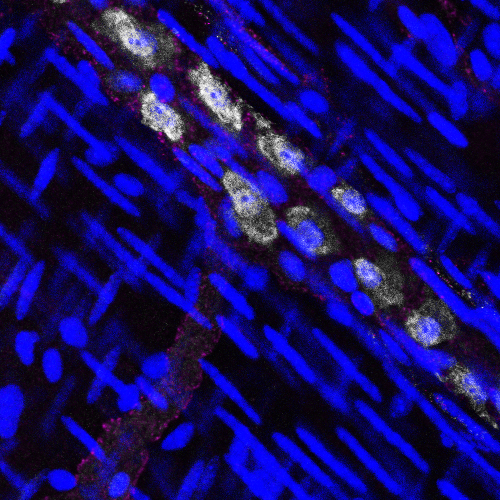

Supplement: Supplementary file 9 — Figure Source Data for EV and Appendix Figures [file 44321_2024_189_MOESM9_ESM.zip › EV and Appendix Figures/Appendix Figures/2/c/IM24h.tif]

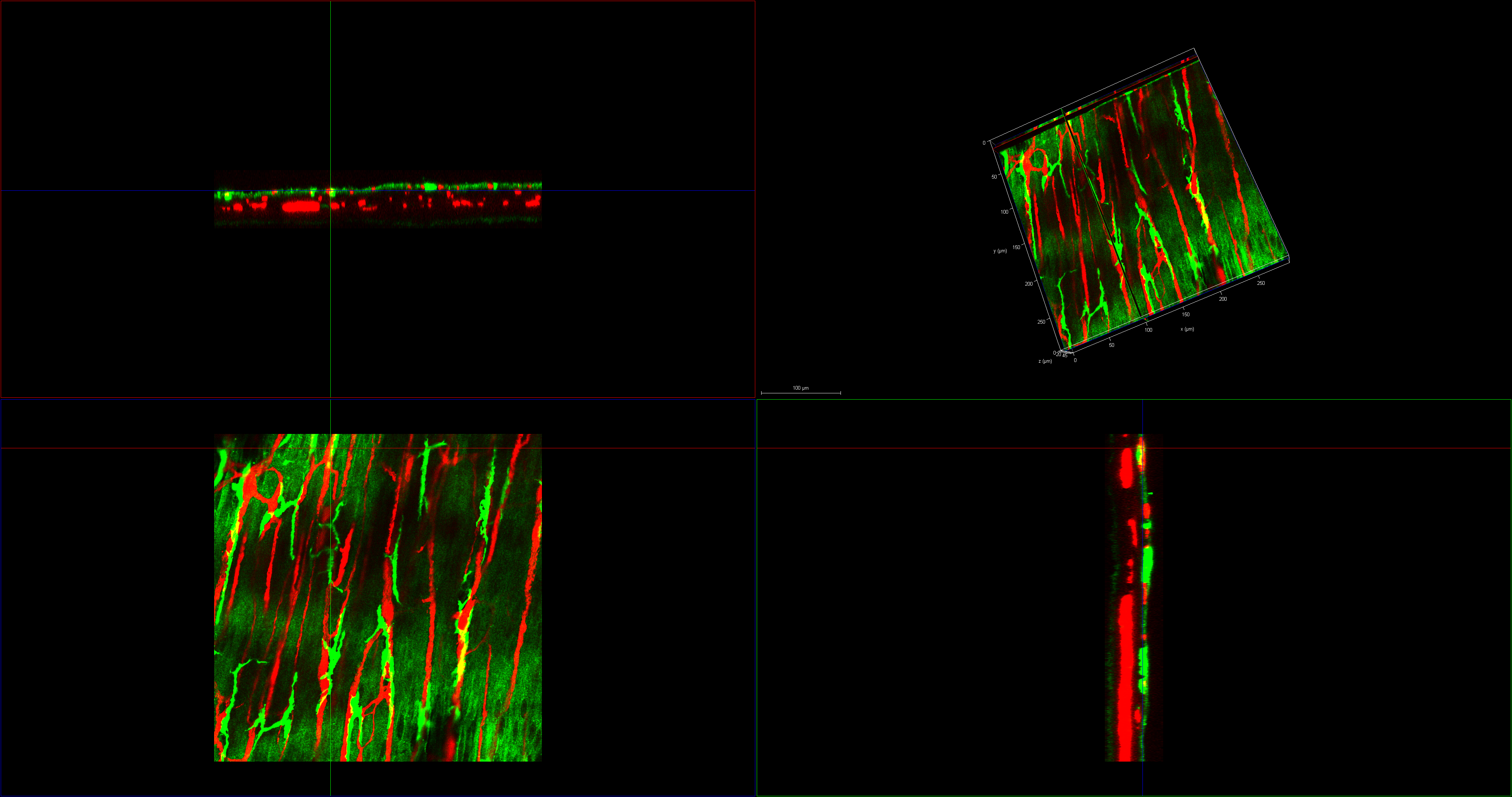

Supplement: Supplementary file 9 — Figure Source Data for EV and Appendix Figures [file 44321_2024_189_MOESM9_ESM.zip › EV and Appendix Figures/Appendix Figures/3/e/Phago of CX3CR1-GFP-tdTomato.tif]
