# Supplementary material for: Macrophage-induced enteric neurodegeneration leads to motility impairment during gut inflammation
Source: EMBO Mol Med. 2025 Jan 6;17(2):301–35. doi: 10.1038/s44321-024-00189-w (PMC11822118; doi:10.1038/s44321-024-00189-w)
Supplement: Supplementary file 10 — Expanded View Figures [file 44321_2024_189_MOESM10_ESM.pdf]

Expanded View Figures

Figure EV1. Intestinal manipulation and inflammation activate enteric neurons.

(**A**) Immunohistochemistry analysis of enteric neurons (myenteric plexus, ANNA1$^+$, gray) in an activated (cFOS$^+$, red) state 24 h post intestinal manipulation (IM). Scale bar 50 µm. (**B**, **C**) RNA-Seq analysis of *ME* samples isolated from IM and control mice at the indicated time points. (**B**) Principal component analysis (PCA) of *ME* RNA samples from POI mice shows a separation of the three groups. (**C**) Heatmaps of genes connected to inflammatory response and migration in IM and control animals. (**D**–**F**) Gene expression analysis of factors involved in synapses (**D**) synaptic transmission (**E**) and synaptic signaling (**F**) in IM and control animals. Bar graphs show the fold gene induction normalized to control (naive) mice. $n = 10$ (control), 9 (IM3h), 10 (IM24h). (**G**, **H**) Mass spectrometry analysis of control and POI mice. (**G**) PCA of *ME* protein samples from POI and control mice shows a clear separation of the three groups. (**H**) Pathway analysis of significantly changed proteins in POI ($P < 0.05$) shows induction of pathways connected to inflammation and migration. $n = 6$. Statistical analysis is based on Fisher's exact *t* test (**H**) and one-way ANOVA (**D**–**F**). Standard deviations are presented as SEM. Source data are available online for this figure.

 © The Author(s)

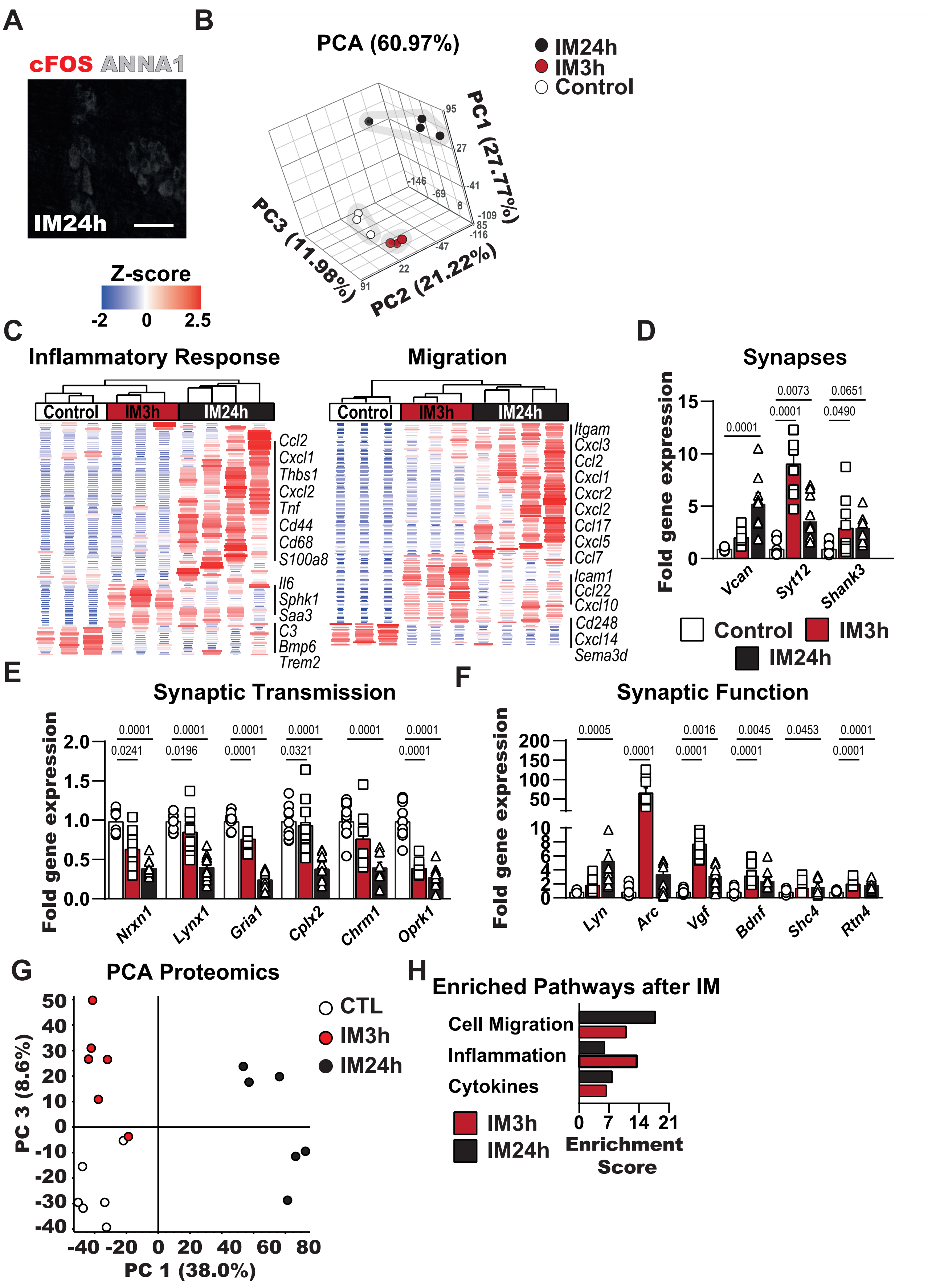
A
cFOS ANNA1
IM24h
B
PCA (60.97%)
IM24h
IM3h
Control
PC1 (27.77%)
PC2 (21.22%)
PC3 (11.98%)
Z-score
-2 0 2.5
C
Inflammatory Response
Control IM3h IM24h
Ccl2
Cxcl1
Thbs1
Cxcl2
Tnf
Cd44
Cd68
S100a8
Il6
Sphk1
Saa3
C3
Bmp6
Trem2
Migration
Control IM3h IM24h
Itgam
Cxcl3
Ccl2
Cxcl1
Cxcr2
Cxcl2
Ccl17
Cxcl5
Ccl7
Icam1
Ccl22
Cxcl10
Cd248
Cxcl14
Sema3d
D
Synapses
Fold gene expression
0.0001
0.0073
0.0001
0.0651
0.0490
Vcan
Syt12
Shank3
Control IM3h
IM24h
E
Synaptic Transmission
Fold gene expression
0.0001 0.0241
0.0001 0.0196
0.0001 0.0001
0.0001 0.0321
0.0001
0.0001 0.0001
Nrxn1
Lynx1
Gria1
Cplx2
Chrm1
Oprk1
F
Synaptic Function
Fold gene expression
0.0005
0.0001
0.0016 0.0001
0.0045 0.0001
0.0453
0.0001 0.0001
Lyn
Arc
Vgf
Bdnf
Shc4
Rtn4
G
PCA Proteomics
CTL
IM3h
IM24h
PC 3 (8.6%)
PC 1 (38.0%)
H
Enriched Pathways after IM
Cell Migration
Inflammation
Cytokines
IM3h
IM24h
0 7 14 21
Enrichment Score

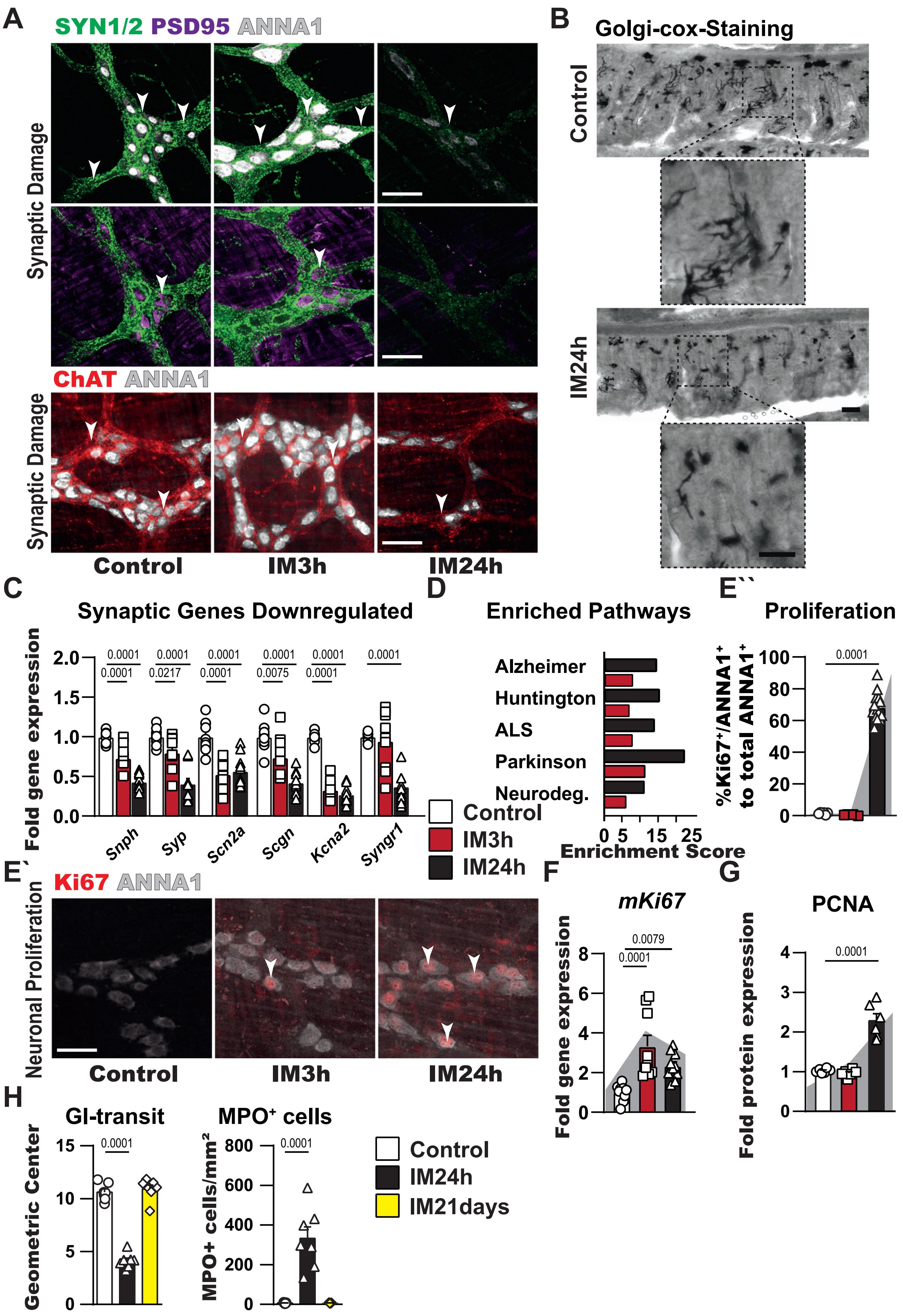
A
SYN1/2 PSD95 ANNA1
Synaptic Damage
ChAT ANNA1
Synaptic Damage
Control
IM3h
IM24h
B
Golgi-cox-Staining
Control
IM24h
C
Synaptic Genes Downregulated
Fold gene expression
Snph
Syp
Scn2a
Scgn
Kcna2
Syngr1
Control
IM3h
IM24h
D
Enriched Pathways
Alzheimer
Huntington
ALS
Parkinson
Neurodeg.
Enrichment Score
E``
Proliferation
%Ki67+/ANNA1+ to total ANNA1+
E`
Ki67 ANNA1
Neuronal Proliferation
Control
IM3h
IM24h
F
mKi67
Fold gene expression
G
PCNA
Fold protein expression
H
GI-transit
Geometric Center
MPO+ cells
MPO+ cells/mm2
Control
IM24h
IM21days

 © The Author(s)

Figure EV2. Intestinal manipulation and inflammation induce enteric neurodegeneration.

(**A**) Immunohistochemistry analysis of myenteric neurons (ANNA1 + , gray), synaptic structures (SYNAPSIN 1/2, green), postsynaptic density protein 95 (PSD95, violet), and choline acetyltransferase (ChAT, red) 3 and 24 h post IM and in control. Scale bar 50 µm. (**B**) Golgi-vox staining of swiss roles from POI and control animals. Scale bar 25 µm. (**C**) Gene expression analysis of factors involved in synaptic transmission in IM and control animals. Bar graphs show the fold gene induction normalized to control mice. $n = 10$ (control), 9 (IM3h), 10 (IM24h). (**D**) Mass spectrometry analysis of control and POI mice. Pathway analysis of significantly changed proteins in POI ($P < 0.05$) shows induction of pathways connected to neurodegenerative diseases. (**E´**) Immunohistochemistry of enteric neurons myenteric plexus, ANNA1$^+$, gray) in a proliferative state (Ki67$^+$, red) 3 and 24 h post IM and in control. A distinct population of double-positive cells (white arrowheads) was detected in POI. Scale bar 50 µm. (**E´´**) Quantification of ANNA1$^+$/Ki67$^+$ cells after IM. Control mice showed almost no double-positive cells, whereas, at IM3h, a low, and at IM24h, many proliferating myenteric neurons were detected. Bar graphs show the mean % of double-positive cells normalized to the total number of ANNA1$^+$ cells. n = 6 (control), 3 (IM3h), 14 (IM24h). (**F**) Gene expression analysis of the proliferation marker *mKi67* in IM and control animals. At IM3h and IM24h, a significant upregulation of *mKi67* gene expression was detected. Bar graphs show the fold gene induction normalized to control mice. $n = 10$ (control), 9 (IM3h), 10 (IM24h). (**G**) Protein expression analysis of the proliferation marker PCNA in IM and control animals. At IM24h, a significant upregulation of PCNA protein was detected by mass spectrometry. Bar graphs show the fold protein induction normalized to control mice. $n = 6$. (**H**) Analysis of POI hallmarks in IM24h, IM21 days and control mice. Gastrointestinal transit and leukocyte (MPO$^+$ cells) infiltration peaks at IM24h. $n = 7$. Statistical analysis is based on Fisher's exact *t* test (**D**) and one-way ANOVA (**E–H**). Standard deviations are presented as SEM. Source data are available online for this figure.

© The Author(s)

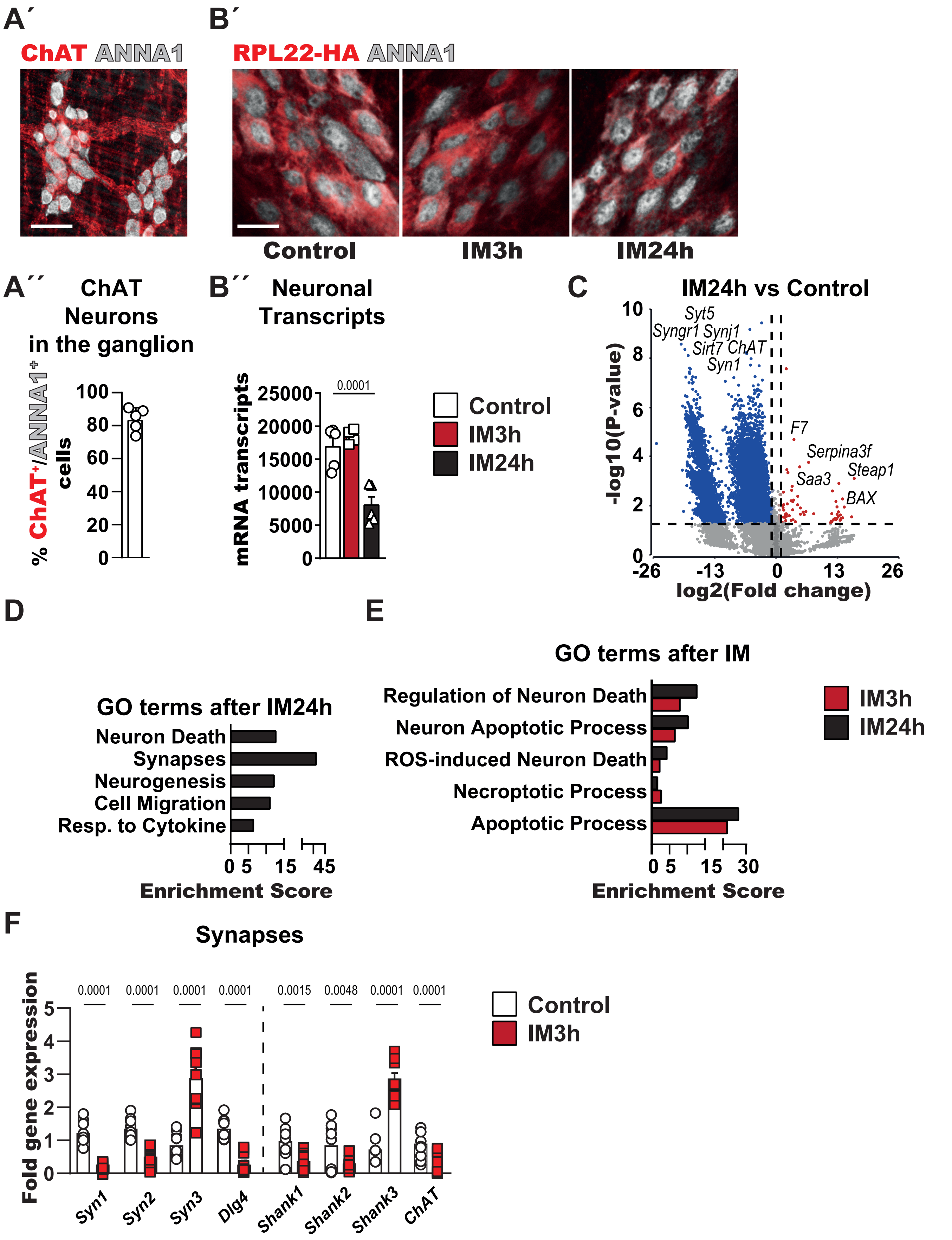
A´
ChAT ANNA1
B´
RPL22-HA ANNA1
Control
IM3h
IM24h
A´´
ChAT Neurons in the ganglion
% ChAT+/ANNA1+ cells
B´´
Neuronal Transcripts
mRNA transcripts
0.0001
Control
IM3h
IM24h
C
IM24h vs Control
-log10(P-value)
log2(Fold change)
Syt5
Syngr1 Synj1
Sirt7 ChAT
Syn1
F7
Serpina3f
Steap1
Saa3
BAX
D
GO terms after IM24h
Neuron Death
Synapses
Neurogenesis
Cell Migration
Resp. to Cytokine
Enrichment Score
E
GO terms after IM
Regulation of Neuron Death
Neuron Apoptotic Process
ROS-induced Neuron Death
Necroptotic Process
Apoptotic Process
Enrichment Score
IM3h
IM24h
F
Synapses
Fold gene expression
0.0001 0.0001 0.0001 0.0001 0.0015 0.0048 0.0001 0.0001
Syn1 Syn2 Syn3 Dlg4 Shank1 Shank2 Shank3 ChAT
Control
IM3h

 © The Author(s)

Figure EV3. Inflammation-triggered neuronal activation leads to enteric neurodegeneration.

(**A´**) Immunohistochemistry analysis of myenteric neurons (ANNA1$^+$, gray) and a neuronal subtype characterized by choline acetyltransferase expression (ChAT$^+$, red) in jejunum *ME* whole-mount specimens. Scale bar 50 µm. (**A´´**) Quantification of ganglionic ANNA1$^+$/ChAT$^+$ cells. Around 80% of all ganglionic myenteric neurons in the healthy small bowel are ChAT$^+$-neurons. Bar graphs show the mean % of double-positive cells normalized to the total number of ANNA1$^+$ cells. $n = 5$. (**B–E**) ChAT^{Cre}/Rpl22$^{HA/+}$ mice were subjected to IM, and *ME* samples collected from IM3h, IM24h, and control animals for immunohistochemistry and RNA-Seq analysis. (**B´**) Immunohistochemistry analysis of myenteric neurons (ANNA1$^+$, gray) and their expression of HA-tagged ribosomes (RPL22-HA$^+$, red). At all disease stages, HA-tagged ribosomes are expressed by ANNA1$^+$ cells. Scale bar 50 µm. (**B´´**) Quantification of neuronal mRNA numbers detected by RNA-Seq analysis after IM. Bar graphs show the mean number of neuronal mRNA transcripts at the analyzed stages of POI development. $n = 5$. (**C**) Volcano plot shows significantly changed genes between the IM24h and control. The plot depicts 78 up- and 13238 downregulated genes with a fold change ≥1.5. $n = 5$ per group. (**D**) Gene ontology (GO) analysis of significantly changed genes ($P < 0.05$) shows strong induction of GO terms connected to "neuronal functions", "inflammation", and "proliferation" in IM24h mice. (**E**) GO analysis of significantly changed genes ($P < 0.05$) showing strong induction of GO terms connected to "neuronal death" in myenteric neurons during POI. (**F**) Gene expression analysis of factors involved in synaptic structures in IM3h and control animals. Bar graphs show the fold gene induction normalized to control mice. $n = 8$ (control), 9 (IM3h). Statistical analysis is based on Fisher's exact *t* test (**C–E**), one-way ANOVA (**B**), and Student´s *t* test (**F**). Standard deviations are presented as SEM. Source data are available online for this figure.

© The Author(s)

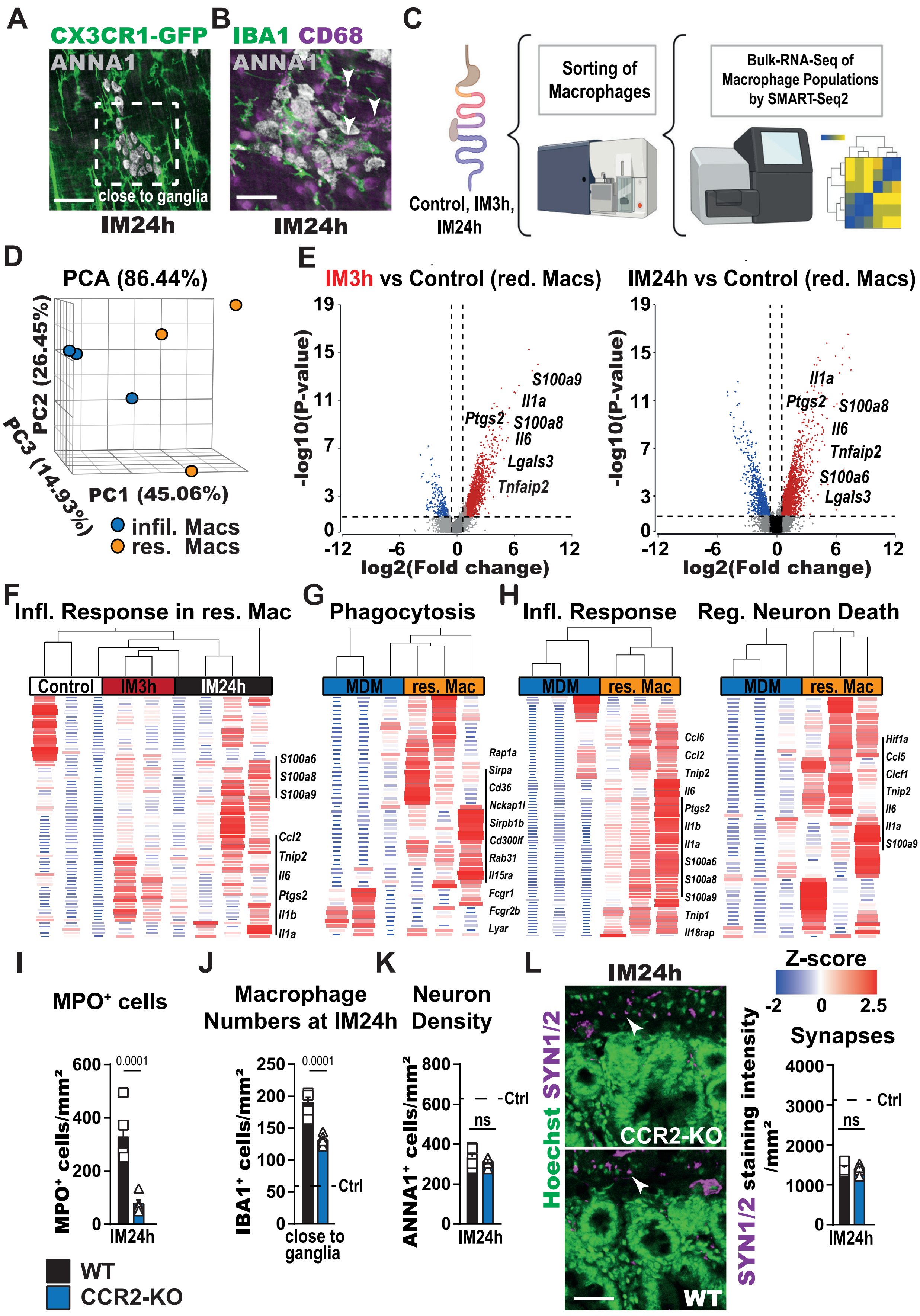
A
CX3CR1-GFP
ANNA1
close to ganglia
IM24h
B
IBA1 CD68
ANNA1
IM24h
C
Control, IM3h, IM24h
Sorting of Macrophages
Bulk-RNA-Seq of Macrophage Populations by SMART-Seq2
D
PCA (86.44%)
PC2 (26.45%)
PC3 (14.93%)
PC1 (45.06%)
infil. Macs
res. Macs
E
IM3h vs Control (red. Macs)
IM24h vs Control (red. Macs)
-log10(P-value)
log2(Fold change)
S100a9
Il1a
Ptgs2
S100a8
Il6
Lgals3
Tnfaip2
S100a6
F
Infl. Response in res. Mac
Control
IM3h
IM24h
S100a6
S100a8
S100a9
Ccl2
Tnip2
Il6
Ptgs2
Il1b
Il1a
G
Phagocytosis
MDM
res. Mac
Rap1a
Sirpa
Cd36
Nckap1l
Sirpb1b
Cd300lf
Rab31
Il15ra
Fcgr1
Fcgr2b
Lyar
H
Infl. Response
Reg. Neuron Death
Ccl6
Ccl2
Tnip2
Il6
Ptgs2
Il1b
Il1a
S100a6
S100a8
S100a9
Tnip1
Il18rap
Hif1a
Ccl5
Clcf1
Tnip2
Il6
Il1a
S100a9
Z-score
-2
0
2.5
I
MPO⁺ cells
MPO⁺ cells/mm²
0.0001
IM24h
WT
CCR2-KO
J
Macrophage Numbers at IM24h
IBA1⁺ cells/mm²
0.0001
Ctrl
close to ganglia
K
Neuron Density
ANNA1⁺ cells/mm²
Ctrl
ns
IM24h
L
IM24h
Hoechst SYN1/2
CCR2-KO
WT
Synapses
SYN1/2 staining intensity /mm²
Ctrl
ns
IM24h

Figure EV4. Inflammatory macrophages are involved in enteric neurodegeneration.

(**A**) Immunohistochemistry analysis of myenteric neurons (ANNA1$^+$, gray) and resident macrophages (CX3CR1-GFP$^+$, green) 24 h post IM. The white dashed square illustrates the definition of macrophages close to ENS ganglia. Scale bar 50 µm. (**B**) Immunohistochemistry analysis of myenteric neurons (ANNA1$^+$, gray) and activated (CD68$^+$, violet, white arrowheads) resident macrophages (Iba1$^+$, green) 24 h post IM and in control. Scale bar 50 µm. (**C**) Schematic overview of the experimental setup to generate a transcriptome analysis in macrophages from POI mice. CX3CR1GFP/+ mice were subjected to IM, and *ME* samples were collected for FACS and subsequent RNA isolation from macrophages for SMART-Seq2 analysis. (**D**) PCA of samples from infiltrating monocyte-derived monocytes and resident macrophages at IM24h shows a separation of both cell types. (**E**) Volcano plots generated from the samples of resident macrophages show regulations (fold change >1.5) at IM3h with 2524 up- and 244 downregulated genes and at IM24h with 2010 up- and 856 downregulated genes. $n = 3$ per group. (**F**) The heatmap from resident macrophages presents significantly changed genes connected to the inflammatory response during POI. (**G**, **H**) The heatmaps from resident and infiltrating monocyte-derived macrophages show phagocyte genes (**G**), inflammatory response genes (**H**), and genes regulating neuron death (**H**) in resident compared to infiltrating cells. (**I**, **L**) Usage of CCR2$^{-/-}$ mice in the POI animal model. At IM24h, CCR2$^{-/-}$ mice have less infiltration of MPO$^+$ cells (**I**) and fewer IBA1$^+$ cells around ganglia (**J**). The reduced infiltration had no effect on neuronal numbers (**K**) and synaptic damage (**L**) in the gut. $n = 5$ (**L**) Immunohistochemistry analysis and quantification of synaptic structures (SYN1/2$^+$, violet, white arrowheads) and Hoechst as counterstaining (green) 24 h post IM in Swiss roles from the jejunum of WT and CCR2$^{-/-}$ mice. Bar graphs show the mean of staining intensity measurements in the indicated groups. $n = 5$. Scale bar 50 µm. Statistical analysis is based on Fisher's exact *t* test (**E**) and Student´s *t* test (**I**–**L**). Standard deviations are presented as SEM. Source data are available online for this figure.

© The Author(s)

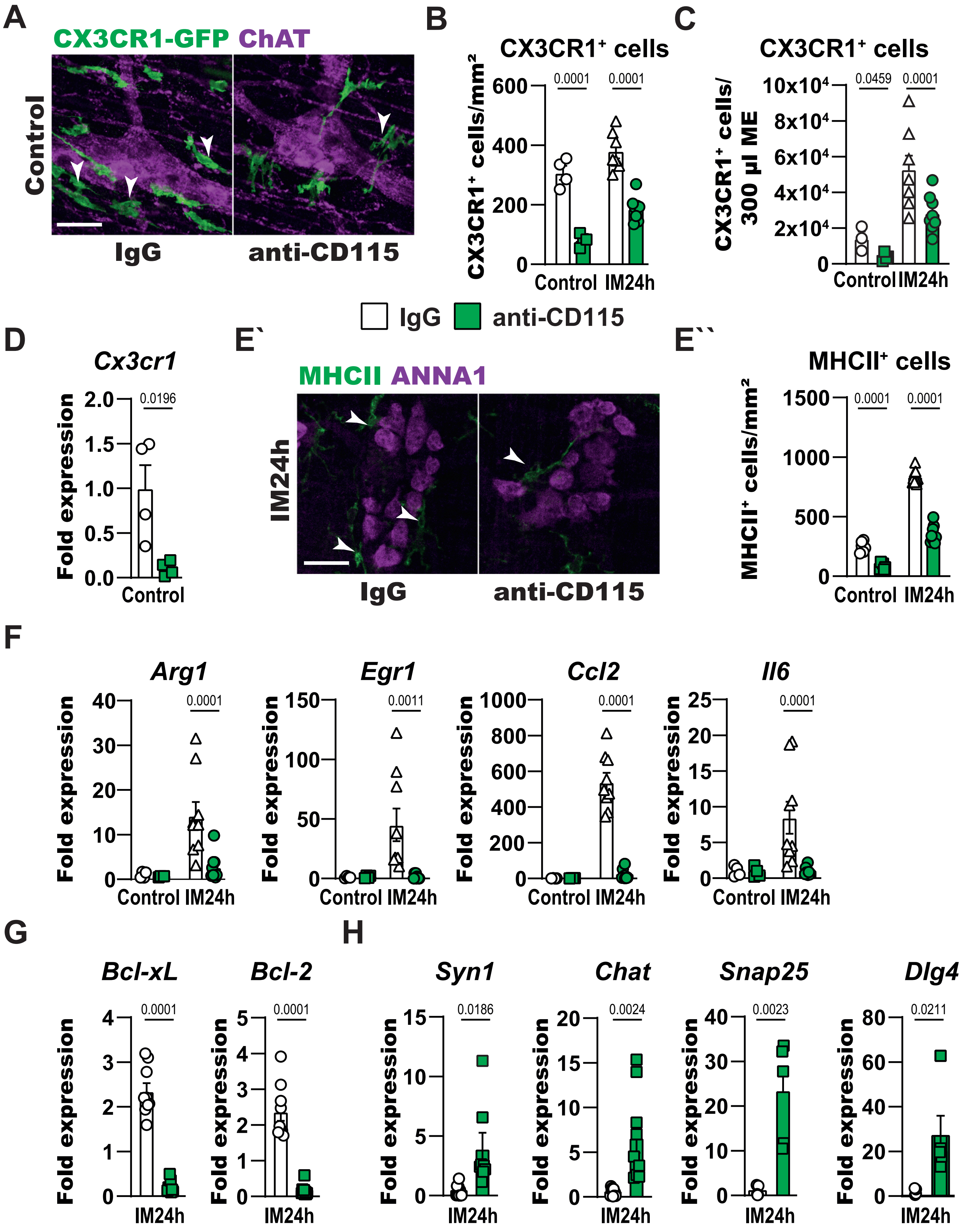
A
CX3CR1-GFP ChAT
Control
IgG
anti-CD115
B
CX3CR1+ cells
CX3CR1+ cells/mm2
0.0001
0.0001
600
400
200
0
Control IM24h
C
CX3CR1+ cells
CX3CR1+ cells/ 300 µl ME
0.0459
0.0001
10x104
8x104
6x104
4x104
2x104
0
Control IM24h
IgG anti-CD115
D
Cx3cr1
Fold expression
0.0196
2.0
1.5
1.0
0.5
0.0
Control
E`
MHCII ANNA1
IM24h
IgG
anti-CD115
E``
MHCII+ cells
MHCII+ cells/mm2
0.0001
0.0001
1500
1000
500
0
Control IM24h
F
Arg1
Fold expression
0.0001
40
30
20
10
0
Control IM24h
Egr1
Fold expression
0.0011
150
100
50
0
Control IM24h
Ccl2
Fold expression
0.0001
1000
800
600
400
200
0
Control IM24h
Il6
Fold expression
0.0001
25
20
15
10
5
0
Control IM24h
G
Bcl-xL
Fold expression
0.0001
4
3
2
1
0
IM24h
Bcl-2
Fold expression
0.0001
5
4
3
2
1
0
IM24h
H
Syn1
Fold expression
0.0186
15
10
5
0
IM24h
Chat
Fold expression
0.0024
20
15
10
5
0
IM24h
Snap25
Fold expression
0.0023
40
30
20
10
0
IM24h
Dlg4
Fold expression
0.0211
80
60
40
20
0
IM24h

 © The Author(s)

Figure EV5. Depleting macrophages in the inflamed gut reduces enteric neurodegeneration.

(**A**) Immunohistochemistry analysis of myenteric neurons (ChAT$^+$, violet) and resident macrophages (CX3CR1-GFP$^+$, green, white arrowheads) in control mice after depletion treatment. Scale bar 50 µm. (**B**) Quantification of CX3CR1-GFP$^+$ cells per mm² jejunum *ME* with and without depletion treatment in control and IM24h animals. Bar graphs show the mean CX3CR1-GFP$^+$ cell number normalized to the *ME* area. $n = 4$ (both controls), 7 (IM24h_IgG), 8 (IM24h_CD115). (**C**) FACS analysis of CX3CR1-GFP$^+$ cells per colon *ME* tissue weight in control and IM24h animals with and without depletion treatment. Bar graphs show the mean CX3CR1-GFP$^+$ cell number normalized to the weight of colon *ME*. $n = 4$ (both controls), 7 (IM24h_IgG), 8 (IM24h_CD115). (**D**) Gene expression analysis of *Cx3cr1* in control animals undergoing anti-CD115 or IgG treatment. Bar graphs show the fold gene induction normalized to controls without depletion of macrophages. n = 4 (**E´**) Immunohistochemistry analysis of macrophages (MHCII$^+$, green, white arrowheads) and myenteric neurons (ANNA1$^+$, violet) in animals 24 post IM undergoing anti-CD115 or IgG treatment. Scale bar 50 µm. (**E´´**) Quantification of MHCII$^+$ cells per mm² *ME* with and without CD115-depletion in control and IM24h animals. Bar graphs show the mean MHCII$^+$ cell number normalized to the jejunum *ME* area. $n = 6$ (both controls), 9 (IM24h_IgG), 9 (IM24h_CD115). (**F–H**) Gene expression analysis of prominent factors involved in POI development (**F**), cell death marker genes (**G**), and synapses (**H**) in IM and control animals. Bar graphs show the fold gene induction normalized to their respective IgG-treated counterparts. $n = 4$ (both controls), 9 (IM24h_IgG), 9 (IM24h_CD115). Statistical analysis is based on one-way ANOVA (**B**, **C**, **E**, **F**) and Student´s *t* test (**D**, **G**, **H**). Standard deviations are presented as SEM. Source data are available online for this figure.

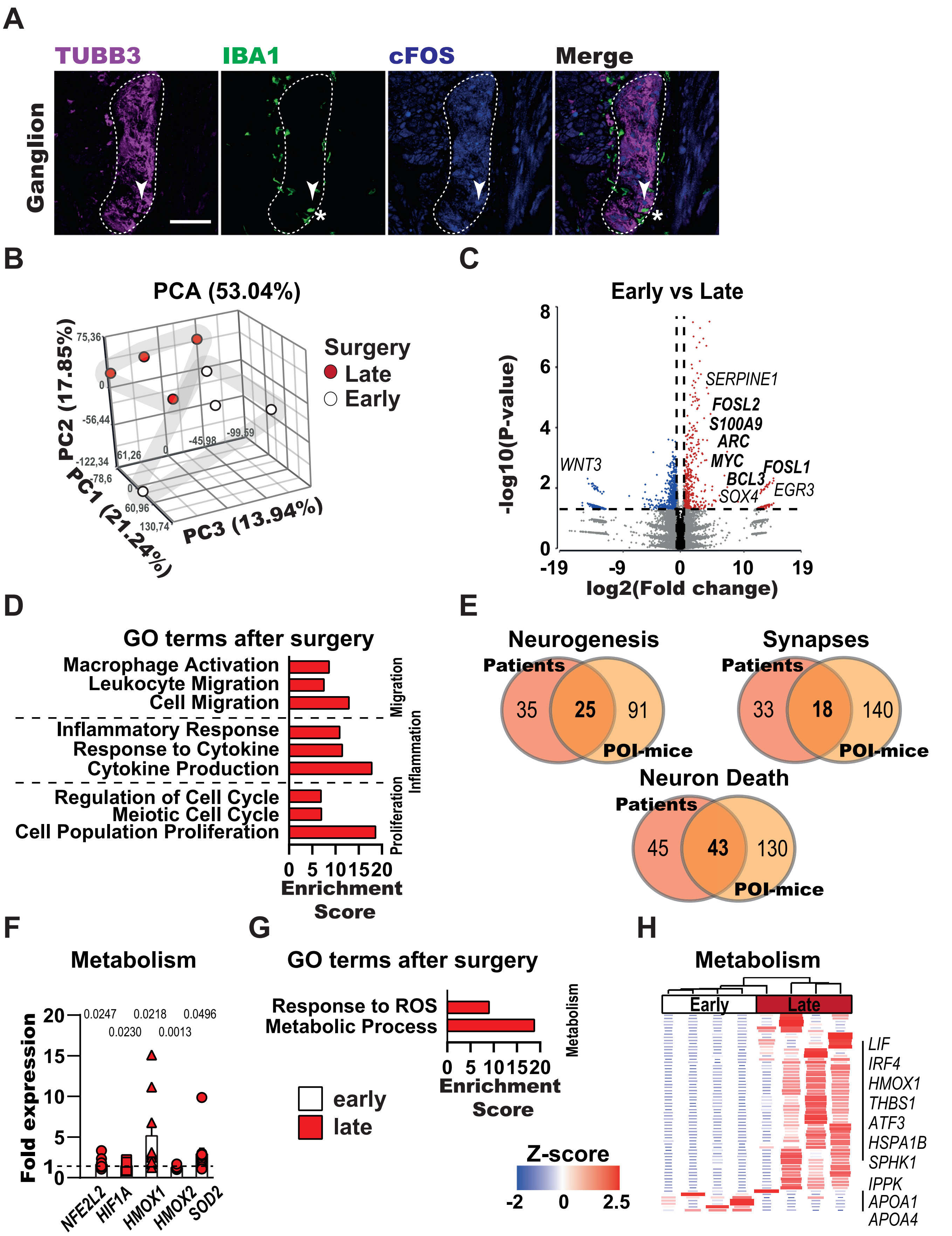
A
TUBB3
IBA1
cFOS
Merge
Ganglion
B
PCA (53.04%)
Surgery
Late
Early
PC2 (17.85%)
PC1 (21.24%)
PC3 (13.94%)
C
Early vs Late
-log10(P-value)
log2(Fold change)
WNT3
SERPINE1
FOSL2
S100A9
ARC
MYC
FOSL1
BCL3
SOX4
EGR3
D
GO terms after surgery
Macrophage Activation
Leukocyte Migration
Cell Migration
Inflammatory Response
Response to Cytokine
Cytokine Production
Regulation of Cell Cycle
Meiotic Cell Cycle
Cell Population Proliferation
Migration
Inflammation
Proliferation
Enrichment Score
E
Neurogenesis
Patients
POI-mice
35 25 91
Synapses
33 18 140
Neuron Death
45 43 130
F
Metabolism
Fold expression
0.0247 0.0218 0.0496
0.0230 0.0013
NFE2L2
HIF1A
HMOX1
HMOX2
SOD2
G
GO terms after surgery
Response to ROS
Metabolic Process
Metabolism
Enrichment Score
early
late
Z-score
-2 0 2.5
H
Metabolism
Early
Late
LIF
IRF4
HMOX1
THBS1
ATF3
HSPA1B
SPHK1
IPPK
APOA1
APOA4

 © The Author(s)

Figure EV6. Gut surgical trauma causes enteric neurodegeneration in patients.

(**A**) Immunohistochemistry analysis of activated (cFOS^{+}, blue) myenteric neurons (TUBB3^{+}, violet) and surrounding resident macrophages (IBA1^{+}, green, white asterisks) in the *ME* of early human jejunum specimens. After surgery, double-positive neurons (white arrowheads) are visible in the enteric ganglia (white dashed line). Scale bar 50 µm. (**B**–**D**) 3′ Bulk RNA-Seq of human jejunal samples collected early and late during a pancreatectomy. (**B**) Principal component analysis (PCA) of the samples from early and late jejunum *ME* samples, representing a separation of both groups. (**C**) Volcano plot showing significantly changed genes between the early and late jejunal specimens. The plot depicts 488 up- and 891 downregulated genes with a fold change ≥1.5. $n = 4$ per group. (**D**) GO analysis of significantly changed genes ($P < 0.05$) shows induction of GO terms connected to POI hallmarks “migration”, “inflammation”, and “proliferation”. (**E**) Venn diagrams show overlapping genes between surgical samples and POI mice connected to “neurogenesis”, “synapses”, and „neuron death“. (**F**) Gene expression analysis shows genes associated with metabolic processes in human jejunum *ME* samples. Bar graphs show the fold gene induction normalized to early jejunum *ME* samples. $n = 9$. (**G**) GO analysis of significantly changed genes ($P < 0.05$) shows a strong induction of GO terms connected to metabolism. Bar graphs present the fold gene induction normalized to early jejunum *ME* samples. (**H**) Heatmap of genes connected to metabolic changes in early and late jejunal specimens. Statistical analysis is based on Fisher’s exact *t* test (**C**, **D**, **G**) and Student´s *t* test (**F**). Standard deviations are presented as SEM. Source data are available online for this figure.

© The Author(s)
